# Supplementary material for: Tuning singlet oxygen generation with caged organic photosensitizers
Source: Nat Commun. 2024 Sep 3;15:7689. doi: 10.1038/s41467-024-51872-y (PMC11372191; doi:10.1038/s41467-024-51872-y)
Supplement: Supplementary file 1 — Supplementary Information [file 41467_2024_51872_MOESM1_ESM.pdf]

Supplementary Information

Tuning singlet oxygen generation with caged organic photosensitizers

Table of Contents

1. Supplementary Methods
   - Materials and Methods
   - Chemical Synthesis
   - Experimental Procedures
2. Supplementary Figures
3. Supplementary Notes
   - Supplementary Notes
   - NMR Spectra
   - Computational Modelling
4. Supplementary References

1. Supplementary Methods

Materials and Methods

Chemicals and solvents were purchased as AR grade. Column chromatography (FC): silica gel Merck 60 (particle size 0.040-0.063 mm) or via Teledyne Isco, eluent systems as indicated. Solvents for all chromatographic purifications were purchased from Fisher Scientific as HPLC grade. Anhydrous solvents and molecular sieves (4 Å beads 8-12 mesh) were purchased from Sigma Aldrich. Chloroformates and carbonates were purchased from Sigma Aldrich. Bases (ethylamine, K_2CO_3, Cs_2CO_3, NaH, propylamine) were purchased from Sigma Aldrich. Uncaged precursors (4-bromo-1,8-naphtalic anhydride, 7-amino-4-methylcoumarin, Nile Blue and Azure B) were purchased from Sigma Aldrich or Fluorochem. TentaGel® HL NH2 (HL12902) resins were purchased from Rapp Polymere GmbH. Gold(III) chloride hydrate, palladium(II) chloride, L-ascorbic acid, pluronic P123 were purchased from Sigma-Aldrich. Microwave assisted chemical reactions were performed using a Biotage® Initiator+. Spectroscopic data was measured on a Synergy HT spectrophotometer (Biotek) and the data analysis was performed using GraphPad Prism 5.0. Preparative HPLC was performed using an Agilent Technologies 1260 system with a Kinetex C_{18} 150 × 21.2 mm column. Eluents: H_2O (0.1% CF_3CO_2H) and ACN (0.1% CF_3CO_2H). Flow: 20.0 mL min^{-1}. Cell imaging was performed with a Leica SP8 fluorescence confocal microscope equipped with a live-cell imaging stage. Images were acquired and processed with the corresponding microscope software, Leica Application Suite X (LAS X) V1.4.6. Synthetic reactions and products were monitored by HPLC-MS using a HPLC Agilent Technologies 1200 with a Kinetex C_{18} 50 × 4.6 mm column and a diode array detector. Eluents: H_2O (0.1% HCOOH) and ACN (0.1% HCOOH). Flow: 1.0 mL min^{-1}. The MS detector was configured with an electrospray ionization source and nitrogen was used as the nebulizer gas. Analytical HPLC was monitored using a HPLC Agilent Technologies system consisting of a 1220 Infinity II autosampler and a 1260 Infinity II detector with a Kinetex 150 x 4.6 mm (5 μm) Phenyl-Hexyl 100 Å column. Flow: 1.5 mL min^{-1}. ^{1}H-NMR spectra were recorded using Bruker Avance III spectrometer operated at 500 MHz. ^{13}C-NMR spectra were recorded using Bruker Avance operated at 126 MHz. Chemical shifts were reported in ppm on the δ scale relative to a residual solvent ($CDCl_3$: δ = 7.26 for ^{1}H-NMR and 77.2 for ^{13}C-NMR, CD_3OD: δ = 3.31 for ^{1}H-NMR and 49.0 for ^{13}C-NMR, DMSO-d6: δ = 2.50 for ^{1}H-NMR and 39.5 for ^{13}C-NMR). Multiplicities are referred by the following abbreviations: s = singlet, d = doublet, t = triplet, dd = doublet

of doublets, ddd = doublet of doublet of doublets, dt = doublet of triplets, q = quartet and m = multiplet.

Computational modelling was performed using Gaussian 16 Rev. B.01.

Chemical Synthesis

General procedures for the synthesis of caged nitrobenzoselenadiazoles

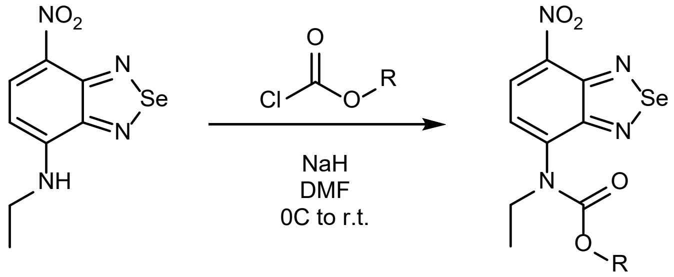

Procedure A for carbamate formation using chloroformates. Compound **1** (1 eq) and NaH (60% suspension in mineral oil, 3 eq) were dissolved in anhydrous DMF under inert atmosphere (N_2). The mixture was stirred at r.t. for 30 min after which it was cooled to 0 °C on ice. A solution of the corresponding chloroformate (1.5 eq) in anhydrous DMF was then added to the cooled mixture dropwise. The reaction mixture was left to stir at 0°C to r.t. (20 min–3 h). Solvent was removed in vacuo and the crude product was purified either by column chromatography (EtOAc in hexanes) or preparative HPLC to render the expected product.

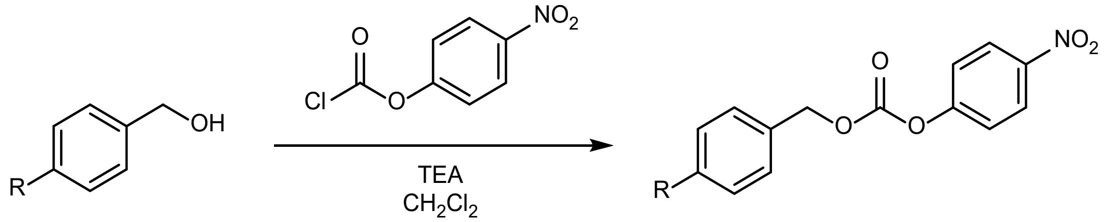

Procedure B for carbonate formation. The appropriate benzyl alcohol (1 eq) and triethylamine (3 eq) were dissolved in DCM (1 mL) and stirred at r.t. for 10 min before the addition of 4-nitrophenylchloroformate (1-1.5 eq). The reaction was stirred at r.t. until completion (0.5-1 hr) and the crude product was purified by column chromatography (DCM or DCM:MeOH, 9:1) to render the expected product.

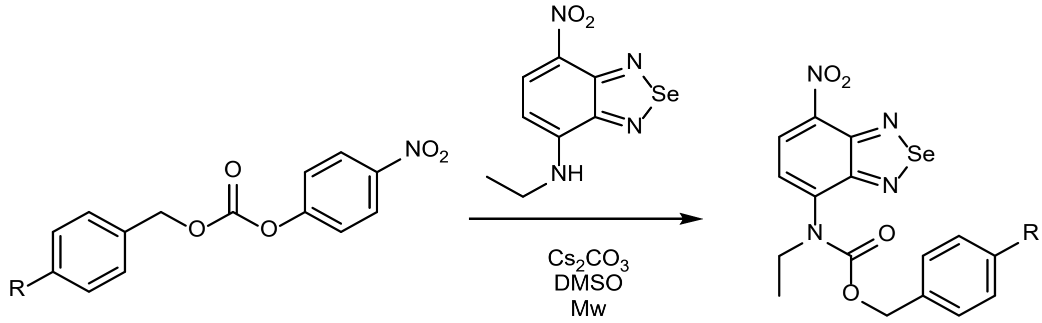

Procedure C for carbamate formation using carbonates. Compound **1** (1 eq) and Cs_2CO_3 (3-5 eq) were dissolved in anhydrous DMSO under N_2 atmosphere in the presence of molecular sieves. The reaction was stirred at r.t. for 30 min before being transferred to a microwave vessel containing molecular sieves and the appropriate nitrophenyl carbonate (1.2-1.5 eq). Reaction was heated at 50 °C for 30 min in a microwave vessel, then the reaction mixture was diluted with H_2O and freeze dried to remove all solvents prior to purification. The crude product was purified either by column chromatography (EtOAc in hexanes) or preparative HPLC to render the expected product.

Compound 1

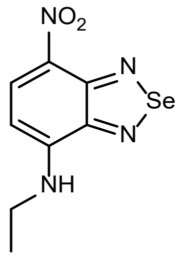

Nitrobenzoselenadiazole fluoride (201 mg, 0.81 mmol, 1 eq), ethylamine hydrochloride (106 mg, 1.22 mmol, 1.5 eq) and triethylamine (227 µl, 1.63 mmol, 2 eq.) were dissolved in MeCN (5 mL) and stirred at 40 °C for 1 h. Solvent was removed in vacuo and the crude product was purified by column chromatography (EtOAc: DCM, 1:9 to 3:7) to give compound **1** as an orange powder (197 mg, 89%).

^{1}H NMR (500 MHz, DMSO-d_6): δ 9.52 (s, 1H), 8.53 (d, J = 9.0 Hz, 1H), 6.41 (d, J = 9.0 Hz, 1H), 3.52 (s, 2H), 1.29 (t, J = 7.2 Hz, 3H); ^{13}C NMR (125 MHz, DMSO-d6) δ 152.6, 152.5, 148.8, 135.9, 128.4, 97.6, 38.1, 14.1; HRMS (m/z, ESI): calcd. for $C_8H_9N_4O_2Se$ $[M+H]^+$: 272.9885, found: 272.9887.

Compound 2

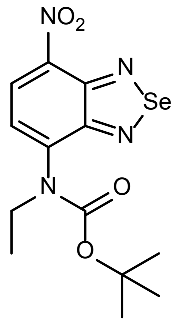

Compound **1** (25 mg, 0.09 mmol, 1 eq) and NaH (60% suspension in mineral oil, 12 mg, 0.30 mmol, 3 eq) were dissolved in anhydrous DMF (250 µL) under N_2. The mixture was stirred at r.t. for 1 h before cooling to 0 °C on ice. A solution of Boc_2O (37 mg, 0.17 mmol, 1.8 eq) in DMF (250 µL) was added to the reaction mixture dropwise. A further portion of DMF (500 µL) was added and the reaction was allowed to stir at r.t. overnight. Solvent was removed in vacuo and the crude product was purified by column chromatography (Hex:EtOAc, 1:1) to give compound **2** as a yellow solid (12 mg, 35%).

^{1}H NMR (500 MHz, DMSO-d_6): δ 8.53 (d, J = 7.9 Hz, 1H), 7.60 (d, J = 8.0 Hz, 1H), 3.80 (q, J = 7.1 Hz, 2H), 1.27 (s, 9H), 1.09 (t, J = 7.1 Hz, 3H); ^{13}C NMR (125 MHz, DMSO-d_6): δ 157.6, 153.6, 151.4, 141.5, 139.5, 128.5, 124.3, 80.6, 45.0, 28.2, 14.3; HRMS (m/z, ESI): calcd. for $C_{13}H_{16}N_4O_4SeNa$ $[M+Na]^+$: 395.0229, found: 395.0233.

Compound 3

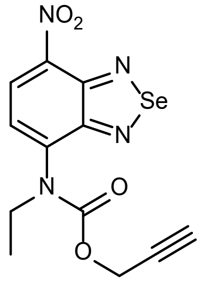

Compound **3** was synthesized according to the procedure A. Compound **1** (25 mg, 0.09 mmol, 1 eq), NaH (11 mg, 0.28 mmol, 3 eq) and propargyl chloroformate (14 µL, 0.14 mmol, 1.5 eq) were used. Upon addition of the chloroformate, the reaction was complete after 20 min at r.t. Solvent was removed in vacuo and the crude product was purified by column chromatography (Hex:EtOAc, 7:3) to give compound **3** as a pale yellow solid (15 mg, 47%).

^1H NMR (500 MHz, DMSO-d_6): δ 8.54 (d, J = 7.9 Hz, 1H), 7.68 (d, J = 7.9 Hz, 1H), 4.66 (d, J = 2.5 Hz, 2H), 3.83 (q, J = 7.2 Hz, 2H), 3.46 (t, J = 2.4 Hz, 1H), 1.11 (t, J = 7.1 Hz, 3H); ^{13}C NMR (125 MHz, DMSO-d_6): δ 157.4, 154.0, 151.3, 140.1, 128.1, 125.3, 79.0, 78.2, 53.6, 45.6, 14.1; HRMS (m/z, ESI): calcd. for $C_{12}H_{11}N_4O_4Se$ $[M+H]^+$: 354.9941, found: 354.9933.

Compound 4

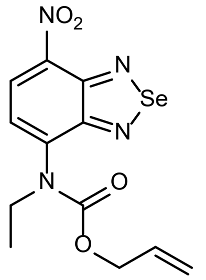

Compound **4** was synthesized according to the procedure A. Compound **1** (53 mg, 0.20 mmol, 1 eq), NaH (2 mg, 0.63 mmol, 3 eq) and allyl chloroformate (31 µL, 0.29 mmol, 1.5 eq) were used. Upon addition of chloroformate, the reaction was complete after 3 h at r.t. Solvent was removed in vacuo and the crude product was purified by column chromatography (Hex:EtOAc, 4:1 to 3:1) to give compound **4** as a yellow solid (38 mg, 55%).

^1H NMR (500 MHz, $CDCl_3$): δ 8.50 (d, J=7.9, 1H), 7.48 (d, J = 7.9 Hz, 1H), 5.84 – 5.73 (m, 1H), 5.13 (dq, J = 6.0 Hz, 1.4, 1H), 5.10 (t, J = 1.4 Hz, 1H), 4.59 (dt, J = 5.6 Hz, 1.4, 2H), 3.95 (q, J = 7.2 Hz, 2H), 1.20 (t, J = 7.1 Hz, 3H); ^{13}C NMR (125 MHz, $CDCl_3$): δ 158.1, 154.7, 152.1, 141.3, 139.7, 132.3, 128.4, 125.0, 118.1, 66.9, 45.7, 14.1; HRMS (m/z, ESI): calcd. for $C_{12}H_{13}N_4O_4Se$ $[M+H]^+$: 357.0097, found: 357.0093.

Compound 5

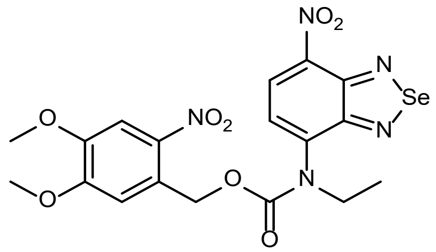

Compound **5** was synthesized using the procedure A. Compound **1**, (30 mg, 0.11 mmol, 1 eq), NaH (13 mg, 0.33 mmol, 3 eq) and 4,5-dimethoxy-1-nitrobenzyl chloroformate (48 mg, 0.17 mmol, 1.5 eq) were used. Upon addition of chloroformate, the reaction was complete after 2 h at r.t. The crude mixture was diluted with H_2O, freeze dried, and purified by preparative HPLC (MeCN:H_2O, 5:95 to 95:5 (+0.1% CF_3CO_2H)) to give compound **5** as a yellow solid (25 mg, 44%).

^{1}H NMR (500 MHz, $CDCl_3$): δ 8.51 (d, J = 7.9 Hz, 1H), 7.62 (s, 1H), 7.51 (d, J = 7.9 Hz, 1H), 6.76 (s, 1H), 5.51 (s, 2H), 3.98 (q, J = 7.1 Hz, 2H), 3.93 (s, 3H), 3.85 (s, 3H), 1.24 (t, J = 7.1 Hz, 3H); ^{13}C NMR (126 MHz, $CDCl_3$): δ 158.1, 154.5, 153.4, 152.1, 148.5, 140.7, 140.2, 140.0, 128.3, 126.7, 125.5, 110.9, 108.4, 65.4, 56.6, 56.5, 45.9, 14.2; HRMS (m/z, ESI): calcd. for $C_{18}H_{17}N_5O_8SeNa$ $[M+Na]^+$: 534.0136, found: 534.0126.

Compound 6

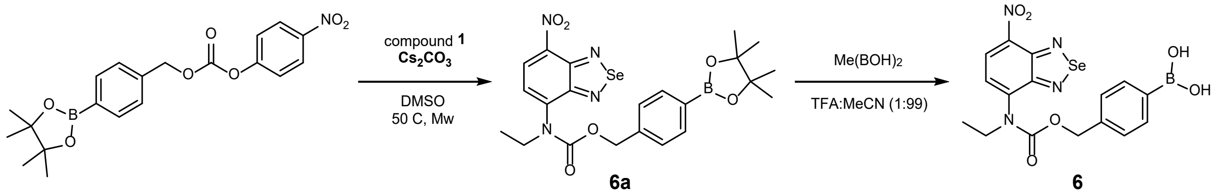

6a **6**

Compound **6a** was synthesized according to the procedure C. Compound **1** (25 mg, 0.09 mmol, 1 eq), Cs_2CO_3 (93 mg, 0.28 mmol, 3 eq) and 9 (55 mg, 0.14 mmol, 1.5 eq) were mixed in 2 mL anhydrous DMSO. After microwave irradiation and freeze drying, the intermediate crude was isolated as a mixture of compounds **6a** and **6**. This mixture was subsequently dissolved in 0.1% TFA in MeCN (2 mL) at r.t. Additional 0.5 mL TFA was then added, followed by methylboronic acid (18 mg, 0.09 mmol, 9 eq) in two portions over 2.5 h, after which the reaction mixture was purified by preparative HPLC (MeCN:H_2O 25:75 to 95:5 (+0.1% CF_3COOH)) to give compound **6** as a yellow solid (7 mg, 17% over two steps).

^{1}H NMR (500 MHz, MeOD): δ 8.51 (d, J = 7.9 Hz, 1H), 7.65 (d, J = 7.5 Hz, 2H), 7.62 (d, J = 7.9 Hz, 1H), 7.10 (d, J = 7.7 Hz, 2H), 5.12 (s, 2H), 3.95 (q, J = 7.1 Hz, 2H), 1.22 (t, J = 7.2 Hz, 3H); ^{13}C NMR (125 MHz, MeOD): δ 157.7, 155.3, 151.5, 140.3, 140.0, 133.6, 127.45, 126.5, 124.8, 67.4, 45.4, 12.8; MS (m/z, ESI): calcd for $C_{16}H_{16}BN_4O_6Se$ $[M+H]^+$: 451.0, found: 451.0.

Compound 7

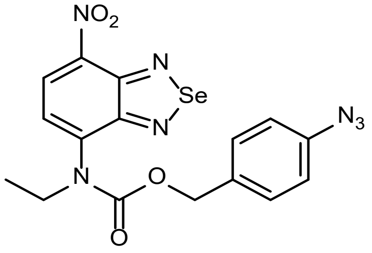

Compound **7** was synthesized according to the procedure C. Compound **1** (25 mg, 0.09 mmol, 1 eq), Cs_2CO_3 (93 mg, 0.28 mmol, 3 eq) and compound **10** (41 mg, 0.13 mmol, 1.5 eq) were mixed in 2 mL anhydrous DMSO. After microwave irradiation and freeze drying, the crude mixture was purified by column chromatography (EtOAc:Hex, 2:8 to 4:6) to give compound **7** as a yellow solid (23 mg, 39%).

^{1}H NMR (500 MHz, DMSO-d_6): δ 8.52 (d, J = 7.9 Hz, 1H), 7.66 (d, J = 7.9 Hz, 1H), 7.21 – 7.16 (m, 2H), 7.05 – 6.99 (m, 2H), 5.03 (s, 2H), 3.83 (q, J = 7.1 Hz, 2H), 1.10 (t, J = 7.1 Hz, 3H); ^{13}C NMR (125 MHz, DMSO-d_6): δ 157.0, 154.2, 150.8, 140.1, 139.5, 139.0, 133.1, 129.4, 127.7, 124.5, 119.0, 66.3, 45.1, 13.7; HRMS (m/z, ESI): calcd. for $C_{16}H_{13}N_7O_4SeNa$ $[M+Na]^+$: 470.0086, found: 470.0077.

Compound 8

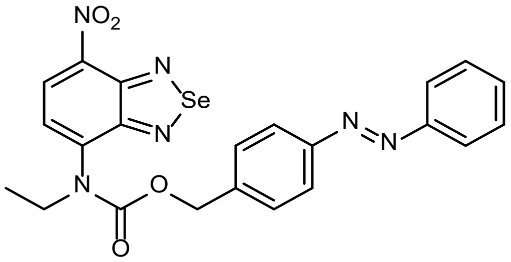

Compound **8** was synthesized following the procedure C. Compound **1** (25 mg, 0.09 mmol, 1 eq) and Cs_2CO_3 (153 mg, 0.47 mmol, 5 eq) and compound **11** (53 mg, 0.14 mmol, 1.5 eq) were mixed in 2 mL anhydrous DMSO. After microwave irradiation and freeze drying, the crude mixture was purified by preparative HPLC (MeCN:H_2O 5:95 to 95:5 (+0.1% HCOOH)) to give compound **8** as a yellow solid (7 mg, 16%).

^{1}H NMR (500 MHz, $CDCl_3$): δ 8.50 (d, J = 7.9 Hz, 1H), 7.94 – 7.88 (m, 2H), 7.87 – 7.80 (m, 2H), 7.56 – 7.45 (m, 4H), 7.34 – 7.28 (m, 2H), 5.21 (s, 2H), 3.98 (q, J = 7.2 Hz, 2H), 1.22 (t, J = 7.1 Hz, 3H); ^{13}C NMR (125 MHz, $CDCl_3$): δ 158.1, 154.9, 152.7, 152.6, 152.2, 141.2, 139.9, 138.8, 131.4, 129.3, 128.8, 128.4, 125.0, 123.1, 123.0, 67.5, 46.0, 14.2; HRMS (m/z, ESI): [M+H]+ calcd. for $C_{22}H_{19}N_6O_4Se$: 511.0627, found: 511.0628.

Compound 9

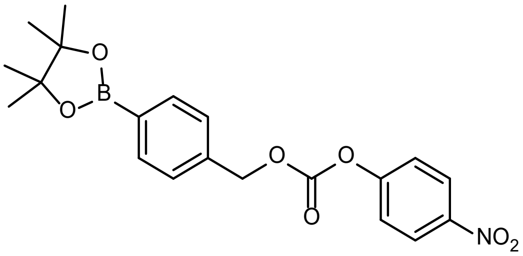

Compound **9** was synthesized according to the procedure B. 4-(hydroxymethyl)phenylboronic acid pinacol ester (50 mg, 0.21 mmol, 1 eq) and 4-nitrophenyl chloroformate (43 mg, 0.21 mmol, 1 eq) were stirred in DCM at r.t. for 30 min until completion. The crude product was purified by column chromatography (DCM:MeOH, 100:0 to 90:10) to render compound **9** as a white solid (33 mg, 38%).

^{1}H NMR (500 MHz, $CDCl_3$): δ 8.30 – 8.24 (m, 2H), 7.88 – 7.83 (m, 2H), 7.46 – 7.41 (m, 2H), 7.41 – 7.34 (m, 2H), 5.31 (s, 2H), 1.35 (s, 12H); ^{13}C NMR (125 MHz, $CDCl_3$): δ 155.5, 152.4, 145.4, 137.1, 135.2, 127.8, 127.6, 125.3, 121.8, 84.0, 70.8, 24.9; HRMS (m/z, ESI): calcd. for $C_{20}H_{22}BNO_7Na$ $[M+Na]^+$: 422.1385, found: 422.1375.

Precursor of compound 10

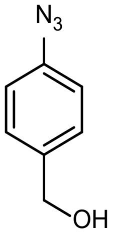

4-Aminobenzyl alcohol (100 mg, 0.81 mmol, 1 eq) was dissolved in 5N HCl solution (0.5 mL) and stirred at r.t. for 20 min before being cooled to 0 °C. An aqueous solution of $NaNO_2$ (84 mg in 2 mL H_2O, 1.22 mmol, 1.5 eq) was added dropwise to the reaction, which was subsequently stirred at 0 °C for further 20 min. NaN_3 (210 mg, 3.23 mmol, 4 eq) was then added portion wise. Next, the reaction was warm to r.t. and react until completion (2.5 h). The crude mixture was then poured into saturated $NaHCO_3$ (50 mLl) and extracted with EtOAc (3×50 mL). The organic layers were combined, washed with brine (3×10 mL), dried over anhydrous $MgSO_4$, and filtered. Solvent was removed under reduced pressure and the crude was purified by column chromatography (Hex:EtOAc, 4:1) to give the expected product as a pale brown solid (91 mg, 75%).

^{1}H NMR (500 MHz, DMSO-d_6): δ 7.40 – 7.32 (m, 2H), 7.11 – 7.04 (m, 2H), 5.21 (t, J = 5.7 Hz, 1H), 4.48 (d, J = 5.6 Hz, 2H); ^{13}C NMR (125 MHz, DMSO-d_6): δ 140.1, 138.1, 128.6, 119.2, 62.8.

Compound 10

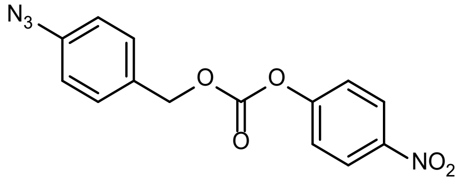

Compound **10** was synthesized according to the procedure B. 4-Azidobenzyl alcohol (19 mg, 0.13 mmol. 1 eq), TEA (53 µL, 0.38 mmol, 3 eq) and 4-nitrophenylchloroformate (31 mg, 0.16 mmol, 1.2 eq) were stirred in DCM at r.t. until completion (1 h). The crude product was purified by column chromatography (DCM) to give compound **10** as a white solid (26 mg, 65%).

^{1}H NMR (500 MHz, $CDCl_3$): δ 8.31 – 8.24 (m, 2H), 7.47 – 7.41 (m, 2H), 7.41 – 7.34 (m, 2H), 7.10 – 7.04 (m, 2H), 5.26 (s, 2H); ^{13}C NMR (125 MHz, $CDCl_3$): δ 155.6, 152.6, 145.6, 141.2, 130.9, 130.6, 125.5, 121.9, 119.5, 70.5; HRMS (m/z, ESI): calcd. for $C_{14}H_{10}N_4O_5Na$ $[M+Na]^+$: 337.0543, found: 337.0542.

Precursor of compound 11

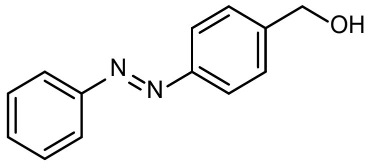

4-Phenylbenzoic acid (50 mg, 0.22 mmol, 1 eq) was dissolved in anhydrous THF (3 mL) under an inert atmosphere (N_2). The solution was then cooled to 0°C before the dropwise addition of $LiAlH_4$ (1.0 M in THF, 440 µL, 0.44 mmol, 2 eq). The reaction was stirred on ice and allowed to warm to r.t. After 1 h, the reaction was quenched with NaOH (aq. 2N, 4 mL) and extracted with EtOAc (3 × 50 mL). The combined organic layers were dried over $MgSO_4$, filtered, and concentrated in vacuo. The crude product was purified by column chromatography (DCM:EtOAc, 8:2) to give expected product as an orange solid (mixture of diastereoisomers, 26 mg, 56%).

^{1}H NMR (500 MHz, DMSO-d_6): δ 7.92 – 7.86 (m, 4H), 7.64 – 7.52 (m, 5H), 5.37 (td, J = 5.7 Hz, 0.8 Hz, 1H), 4.62 (d, J = 5.7 Hz, 2H); ^{13}C NMR (125 MHz, DMSO-d_6): δ 152.4, 151.3, 147.0, 131.8, 129.9, 129.4, 127.6, 127.1, 122.9, 122.9, 120.5, 120.2, 62.9; HRMS (m/z, ESI): calcd. for $C_{13}H_{13}N_2O$ $[M+H]^+$: 213.1022, found: 213.1026.

Compound 11

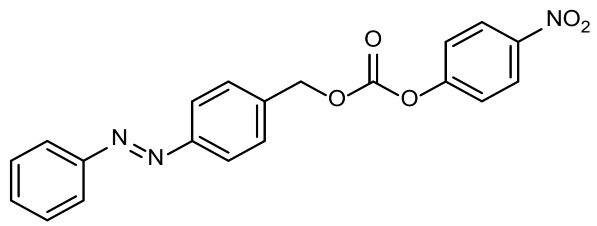

Compound **11** was synthesized according to procedure B. 4-Phenylazobenzyl alcohol (20 mg, 0.09 mmol, 1 eq) and 4-nitrophenylchloroformate (28 mg, 0.14 mmol, 1.5 eq) were stirred in DCM at r.t. until completion (1 h). The crude product was purified by column chromatography (DCM) to give compound 11 as a yellow solid (26 mg, 73%).

^{1}H NMR (500 MHz, $CDCl_3$): δ 8.33 – 8.29 (m, 2H), 8.01 – 7.98 (m, 2H), 7.98 – 7.94 (m, 2H), 7.64 – 7.60 (m, 2H), 7.59 – 7.50 (m, 3H), 7.45 – 7.41 (m, 2H), 5.40 (s, 2H); ^{13}C NMR (125 MHz, $CDCl_3$): δ 155.5, 152.9, 152.6, 152.5, 145.5, 136.8, 131.4, 129.3, 129.2, 125.4, 123.2, 123.0, 121.8, 70.4; HRMS: (m/z, ESI): calcd. for $C_{20}H_{16}N_3O_5$ $[M+H]^+$: 378.1085, found: 378.1091.

Compound 12

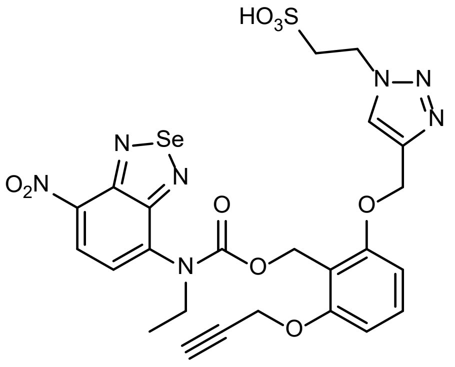

Compound **13** (40 mg, 0.08 mmol, 1 eq) and 2-azidoethanesulfonate sodium salt (20 mg, 0.12 mmol, 1.5 eq) were dissolved in DMF (2 mL). A mixture of L-histidine (36 mg, 0.24 mmol, 3 eq) and $CuSO_4$ (37 mg, 0.24 mmol, 3 eq) in H_2O (1 mL) was added, followed by sodium ascorbate (92 mg, 0.48 mmol, 6 eq) in H_2O (1 mL) and the reaction was allowed to stir at r.t. for 20 min. The reaction mixture was then diluted with DMF:H_2O (1:1, 10 mL), centrifuged and filtered to remove insoluble precipitate and purified by preparative HPLC (MeCN:H_2O, 5:95 to 95:5 (+0.1% CF_3COOH)) to render compound **12** as a yellow solid (7 mg, 13%).

^{1}H NMR (500 MHz, DMSO-d_6) δ 8.47 (d, J = 7.9 Hz, 1H), 8.26 (s, 1H), 7.57 (d, J = 8.0 Hz, 1H), 7.27 (t, J = 8.4 Hz, 1H), 6.85 (d, J = 8.4 Hz, 1H), 6.68 (d, J = 8.4 Hz, 1H), 5.09 (s, 2H), 5.08 (s, 2H), 4.73 (d, J = 2.3 Hz, 2H), 4.62 – 4.59 (m, 2H), 3.78 (d, J = 7.1 Hz, 2H), 3.51 (t, J = 2.3 Hz, 1H), 3.00 (dd, J = 8.6 Hz, 7.0 Hz, 2H), 1.05 (t, J = 7.1 Hz, 3H); ^{13}C NMR (125 MHz, DMSO-d_6) δ 158.1, 157.2, 155.0, 151.3, 143.0, 140.8, 139.6, 130.8, 128.2, 125.1, 124.8, 118.8, 116.8, 112.5, 106.6, 106.3, 79.6, 78.7, 62.5, 57.3, 56.6, 51.6, 47.1, 45.3, 14.1; HRMS (m/z, ESI): calcd. for $C_{24}H_{23}N_7O_9SSeNa$ $[M+Na]^+$: 688.0335; found: 688.0337.

Compound 13

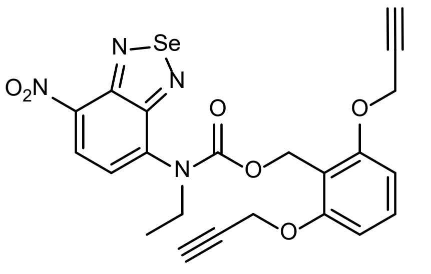

Compound **1** (60 mg, 0.22 mmol, 1 eq) was dissolved in anhydrous DMF (1 mL) under N_2 atmosphere in the presence of Cs_2CO_3 (144 mg, 0.44 mmol, 2 eq) and molecular sieves, and the mixture was allowed to stir at r.t. for 1 h. It was then cooled to 0°C and a solution of dialkyne carbonate (100 mg, 0.26 mmol, 1.2 eq) in anhydrous DMF (1 mL) was added via a syringe. Reaction was heated at 50 °C for 30 min in a microwave vessel. Solvents were removed under reduced pressure and the crude was purified by column chromatography (Hex:EtOAc, 6:4) to give compound 13 as an orange solid (38 mg, 36%).

^1H NMR (500 MHz, $CDCl_3$, mixture of rotamers) δ (major rotamer) 8.48 (d, J = 8.0 Hz, H), 7.49 (d, J = 8.0 Hz, 1H), 7.26 (dd, J = 8.4, 0.9 Hz, 1H), 6.75 (d, J = 8.6 Hz, 1H), 6.67 (d, J = 8.4 Hz, 1H), 5.33 (s, 1H), 4.84 (s, 1H), 4.77 (d, J = 2.4 Hz, 2H), 4.67 (d, J = 2.4 Hz, 2H), 4.00 (q, J = 7.1 Hz, 2H), 2.54 (t, J = 2.4 Hz, 1H), 2.52 (t, J = 2.4 Hz, 1H), 1.22 (t, J = 7.1 Hz, 3H); ^{13}C NMR (125 MHz, $CDCl_3$, mixture of rotamers) δ 161.9, 157.7, 157.4, 156.6, 155.3, 152.0, 141.5, 139.1, 130.3, 130.2, 129.1, 128.6, 126.2, 124.7, 118.7, 115.6, 113.3, 106.3, 106.2, 106.0, 78.4, 75.8, 57.5, 56.5, 56.4, 54.6, 45.3, 34.0; HRMS (m/z, ESI): calcd. for $C_{22}H_{19}N_4O_6Se$ $[M+H]^+$: 515.0464; found: 515.0455.

Precursor of compound 14a

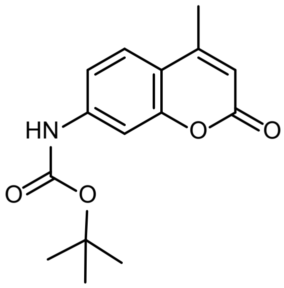

7-Amino-4-methylcoumarin (100 mg, 0.57 mmol, 1 eq) was dissolved in dioxane (3 mL) and Boc_2O (249 mg, 1.14 mmol, 2 eq) was added. Reaction was then heated to 90°C overnight. Solvents were removed under reduced pressure and the crude was purified by column chromatography (Hex:EtOAc, 1:1) to give the expected compound as a white solid (146 mg, 93%).

^1H NMR (500 MHz, $CDCl_3$) δ 7.52 (d, J = 9.1 Hz, 1H), 7.45 – 7.35 (m, 2H), 6.80 (s, 1H), 6.19 (d, J = 1.2 Hz, 1H), 2.42 (d, J = 1.2 Hz, 3H), 1.56 (s, 9H); ^{13}C NMR (125 MHz, $CDCl_3$) δ 161.2, 154.5, 152.2, 152.1, 142.0, 125.2, 115.2, 114.3, 112.9, 105.7, 81.5, 28.3, 18.5; HRMS (m/z, ESI): calcd. for $C_{15}H_{18}NO_4$ $[M+H]^+$: 276.1230; found: 276.1231.

Compound 14a

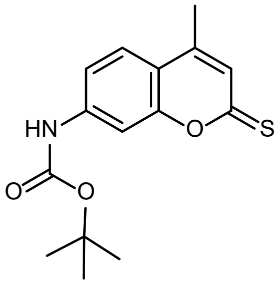

Boc-7-amino-4-methylcoumarin (146 mg, 0.53 mmol, 1 eq) was dissolved in toluene (4 mL) and Lawesson's reagent (215 mg, 0.53 mmol, 1 eq), was added. Reaction was then refluxed at 110 °C for 90 min until completion. Solvents were removed under reduced pressure and the crude was purified by column chromatography (DCM) to give compound 14 as a yellow solid (91 mg, 59%).

^1H NMR (500 MHz, $CDCl_3$) δ 7.56 (d, J = 8.7 Hz, 1H), 7.53 (d, J = 2.1 Hz, 1H), 7.48 (dd, J = 8.7 Hz, 2.0 Hz, 1H), 7.10 (d, J = 1.1 Hz, 1H), 6.80 (s, 1H), 2.36 (s, 3H), 1.56 (s, 9H); ^{13}C NMR (125 MHz, $CDCl_3$) δ 197.5, 157.1, 151.9, 144.4, 142.4, 127.3, 125.3, 116.8, 115.5, 105.3, 81.8, 28.3, 17.9; HRMS (m/z, ESI): calcd. for $C_{15}H_{17}NO_3SNa$ $[M+Na]^+$: 314.0821, found: 314.0819.

Precursor of compound 14b

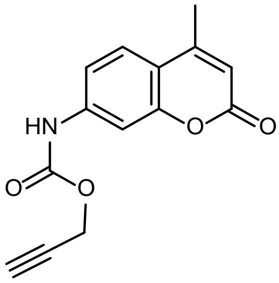

7-Amino-4-methylcoumarin (200 mg, 1.14 mmol, 1 eq) was dissolved in DCM (6 mL) and then cooled to 0 °C. Pyridine (101 mL, 1.25 mmol, 1.1 eq) was then added, followed by propargyl chloroformate (222 mL, 2.28 mmol, 2 eq). Reaction was then allowed to warm to r.t. and stirred overnight under N2 atmosphere. The crude was then poured into an aqueous 0.5 M HCl solution (40 mL) to give a precipitate that was collected by filtration and dried under vacuum to render the expected product as a white solid (264 mg, 90%).

^1H NMR (500 MHz, DMSO-d_6) 10.37 (s, 1H), 7.71 (d, J = 8.7 Hz, 1H), 7.54 (d, J = 2.0 Hz, 1H), 7.42 (dd, J = 8.7, 2.0 Hz, 1H), 6.25 (s, 1H), 4.82 (s, 2H), 3.61 (t, J = 2.4 Hz, 1H), 2.40 (s, 3H); ^{13}C NMR (125 MHz, DMSO-d_6) δ 160.5, 154.3, 153.6, 152.9, 142.9, 126.6, 115.1, 114.8, 112.5, 105.1, 79.1, 78.4, 52.9, 18.5; HRMS (m/z, ESI): calcd. for $C_{14}H_{12}NO_4^+$ $[M+H]^+$: 258.0761; found: 258.0763.

Compound 14b

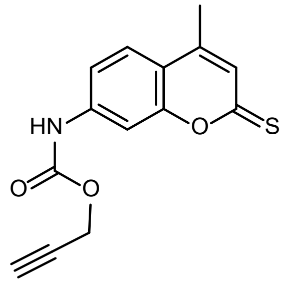

Poc-7-amino-4-methylcoumarin (90 mg, 0.35 mmol, 1 eq) was dissolved in toluene (3 mL) and Lawesson's reagent (142 mg, 0.35 mmol, 1 eq), was added. Reaction was then refluxed to 110°C for 3 h. Solvents were removed under reduced pressure and the crude was purified by column chromatography (DCM:EtOAc, 98:2) to give compound **14b** as a yellow solid (33 mg, 35%).

^{1}H NMR (500 MHz, $CDCl_3$) δ 7.61 (dd, J = 6.4, 5.5 Hz, 2H), 7.45 (dd, J = 8.6, 1.8 Hz, 1H), 7.12 (d, J = 1.1 Hz, 1H), 6.97 (s, 1H), 4.85 (d, J = 2.5 Hz, 2H), 2.57 (t, J = 2.5 Hz, 1H), 2.37 (d, J = 1.1 Hz, 3H); ^{13}C NMR (125 MHz, $CDCl_3$) δ 197.4, 157.0, 151.8, 144.0, 141.2, 127.7, 125.4, 124.8, 117.4, 115.6, 105.8, 75.5, 53.3, 17.9; HRMS (m/z, ESI): calcd. for $C_{14}H_{12}NO_3S$ $[M+H]^+$: 274.0532; found: 274.0530.

Precursor of compound 15

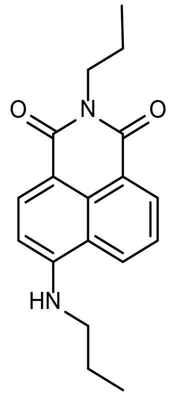

4-Bromo-1,8-naphthalic anhydride (139 mg, 0.5 mmol, 1 eq) was dissolved in DMSO (5 mL) and propylamine (412 mL, 5 mmol, 10 eq) was added. The reaction was heated at 160 °C for 90 min in a microwave vessel, and the reaction mixture was diluted with H_2O and freeze dried to give the crude product that was purified by column chromatography (DCM:MeOH, 98:2) to render the expected product as an orange solid (98 mg, 66%).

^{1}H NMR (500 MHz, $CDCl_3$) δ 8.61 (dd, J = 7.3, 1.0 Hz, 1H), 8.49 (d, J = 8.4 Hz, 1H), 8.10 (dd, J = 8.5 Hz, 0.9 Hz, 1H), 7.64 (dd, J = 8.4 Hz, 7.3 Hz, 1H), 6.75 (d, J = 8.4 Hz, 1H), 5.26 (s, 1H), 4.17 – 4.14 (m, 2H), 3.41 (td, J = 7.2 Hz, 5.3 Hz, 2H), 1.87 (dd, J = 14.5 Hz, 7.3 Hz, 2H), 1.78 (dt, J = 9.0 Hz, 7.5 Hz, 2H), 1.14 (t, J = 7.4 Hz, 3H), 1.03 (t, J = 7.4 Hz, 3H); ^{13}C NMR (125 MHz, $CDCl_3$) δ 164.7, 164.2, 149.3, 134.4, 131.1, 129.8, 125.6, 124.7, 123.3, 120.2, 110.4, 104.4, 45.5, 41.7, 22.3, 21.5, 11.7, 11.6; HRMS (m/z, ESI): calcd. for $C_{18}H_{21}N_2O_2$ $[M+H]^+$: 297.1526, found: 297.1529.

Compound 15

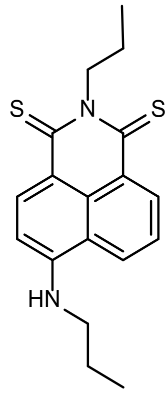

Propylnaphthalimide (90 mg, 0.3 mmol, 1 eq) was dissolved in toluene (15 mL) and Lawesson's reagent (614 mg, 1.5 mmol, 5 eq), was added. Reaction was then refluxed to 110°C for 16 h until completion. Solvents were removed under reduced pressure and the crude was purified by column chromatography (DCM:Hex, 6:4) to give compound **15** as a blue solid (99 mg, quantitative yield).

^{1}H NMR (500 MHz, $CDCl_3$) δ 8.99 (d, J = 7.6 Hz, 1H), 8.90 (d, J = 8.9 Hz, 1H), 8.01 (d, J = 8.2 Hz, 1H), 7.49 (t, J = 8.0 Hz, 1H), 6.69 (d, J = 9.0 Hz, 1H), 5.59 (br, s, 1H), 5.36 (br, s, 2H), 3.41 (t, J = 7.2 Hz, 2H), 2.03 – 1.96 (m, 2H), 1.88 (dd, J = 14.6 Hz, 7.3 Hz, 2H), 1.14 (t, J = 7.4 Hz, 3H), 1.05 (t, J = 7.4 Hz, 3H); ^{13}C NMR (125 MHz, $CDCl_3$) δ 190.5, 150.2, 142.7, 138.6, 130.0, 125.6, 125.4, 125.1, 120.0, 119.2, 106.9, 56.3, 45.5, 22.3, 18.9, 11.6, 11.0; HRMS (m/z, ESI): calcd. for $C_{18}H_{21}N_2S_2$ $[M+H]^+$: 329.1141, found: 329.1144.

Compound 15a

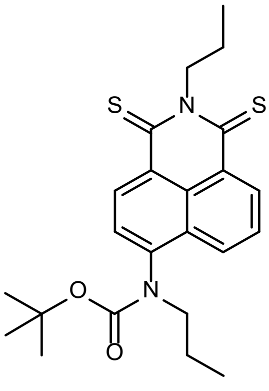

Compound **15** (20 mg, 0.06 mmol, 1 eq) and NaH 60% in mineral oil (10 mg, 0.18 mmol, 3 eq) were placed under N_2 atmosphere, then dry THF (1.5 mL) was added, and the mixture was allowed to stir at r.t. for 30 min. The reaction mixture was then cooled to 0°C and a solution of Boc_2O (39 mg, 0.18 mmol, 3 eq) in dry THF (0.5 mL) was added via a syringe. Reaction was then allowed to warm to r.t. for 7 h, solvents were removed and the crude was purified by column chromatography (DCM:Hex, 55:45) to give compound **15a** as a brown solid (5 mg, 20%).

^{1}H NMR (500 MHz, $CDCl_3$) δ 8.99 – 8.87 (m, 2H), 8.14 (d, J = 8.2 Hz, 1H), 7.67 (t, J = 8.0 Hz, 1H), 7.46 (d, J = 8.0 Hz, 1H), 5.32 – 5.25 (m, 2H), 3.87 – 3.73 (m, 1H), 3.61 (d, J = 5.0 Hz, 1H), 2.00 – 1.92 (m, 2H), 1.67 – 1.58 (m, 2H), 1.28 (s, 9H), 1.05 (t, J = 7.4 Hz, 3H), 0.90 (t, J = 7.4 Hz, 3H); ^{13}C NMR (125 MHz, $CDCl_3$) δ 191.0, 190.4, 154.2, 144.9, 138.1, 138.0, 129.9, 129.2, 128.9, 128.3, 127.9, 127.8, 127.4, 124.4, 121.8, 81.0, 56.4, 29.7, 28.1, 18.9, 11.2, 10.9; HRMS (m/z, ESI): calcd. for $C_{23}H_{29}N_2O_2S_2$ $[M+H]^+$: 429.1665, found: 429.1669.

Compound 15b

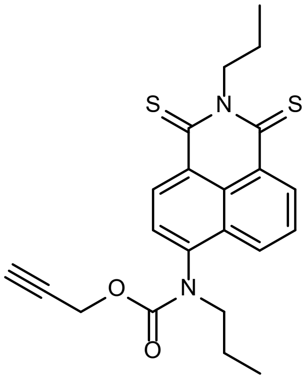

Compound **15** (43 mg, 0.14 mmol, 1 eq) and NaH 60% in mineral oil (24 mg, 0.43 mmol, 3 eq) were placed under N_2 atmosphere, then dry THF (2 mL) was added, and the mixture was allowed to stir at r.t. for 30 min. The reaction mixture was cooled to 0°C and a solution of propargyl chloroformate (43 mL, 0.43 mmol, 3 eq) in dry THF (2 mL) was added via a syringe. The reaction was allowed to warm to r.t. for 1 h, solvents were removed and the crude was purified by column chromatography (Hex:EtOAc, 7:3) to give compound **15b** as an orange solid (5 mg, 20%).

^{1}H NMR (500 MHz, $CDCl_3$) δ 8.72 – 8.62 (m, 2H), 8.19 (dd, J = 8.5, 1.0 Hz, 1H), 7.81 (dd, J = 8.4 Hz, 7.3 Hz, 1H), 7.71 – 7.56 (m, 1H), 4.91 – 4.47 (m, 2H), 4.24 – 4.12 (m, 2H), 4.01 – 3.88 (m, 1H), 3.72 – 3.58 (m, 1H), 2.52 – 2.24 (m, 1H), 1.86 – 1.75 (m, 2H), 1.70 – 1.60 (m, 2H), 1.04 (t, J = 7.4 Hz, 3H), 0.94 (t, J = 7.4 Hz, 3H); ^{13}C NMR (125 MHz, $CDCl_3$) δ 164.1, 163.6, 154.6, 131.5, 131.3, 129.3, 129.2, 127.5, 126.7, 123.4, 122.3, 78.1, 74.6, 53.4, 53.1, 42.0, 21.4, 11.5, 11.1; HRMS (m/z, ESI): calcd. for $C_{22}H_{23}N_2O_2S_2$ $[M+H]^+$: 411.1125; found: 411.1120.

Compound 16a

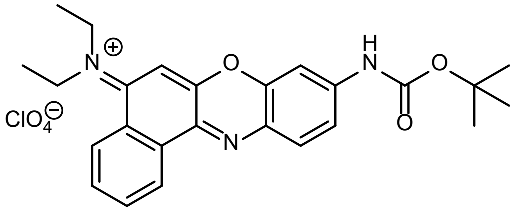

Nile Blue perchlorate (50 mg, 0.12 mmol, 1 eq) and NaH 60% in mineral oil (20 mg, 0.36 mmol, 3 eq) were placed under a N_2 atmosphere, then dry THF (3 mL) was added, and the mixture was allowed to stir at r.t. for 30 min. The reaction mixture was cooled to 0°C and a solution of Boc_2O (78 mg, 0.18 mmol, 3 eq) in dry THF (2 mL) was added via a syringe. The reaction was allowed to warm to r.t. for 30 min, diluted with MeCN:H_2O and freeze dried to give a dark residue that was triturated with Et_2O to give compound **16a** as a blue solid (63 mg, quantitative yield).

^{1}H NMR (500 MHz, DMSO-d_6) δ (dd, J = 8.0, 0.8 Hz, 1H), 8.27 (dd, J = 8.0, 0.9 Hz, 1H), 7.81 – 7.76 (m, 1H), 7.74 – 7.65 (m, 1H), 7.61 – 7.56 (m, 1H), 6.80 (dd, J = 9.1, 2.7 Hz, 1H), 6.66 (d, J = 2.7 Hz, 1H), 6.26 (d, J = 2.7 Hz, 1H), 3.50 (q, J = 7.0 Hz, 4H), 3.18 (s, 1H), 1.56 (s, 9H), 1.17 (t, J = 7.0 Hz, 6H); ^{13}C NMR (125 MHz, DMSO-d6) δ 163.0, 158.0, 151.1, 149.4, 146.8, 139.4, 131.9, 131.6, 131.1, 130.9, 130.5, 125.0, 124.8, 124.0, 110.4, 98.5, 96.7, 81.2, 49.1, 44.9, 28.3, 12.9; HRMS (m/z, ESI): calcd. for $C_{25}H_{28}N_3O_3$ $[M]^+$: 418.2125; found: 418.2141.

Compound 16b

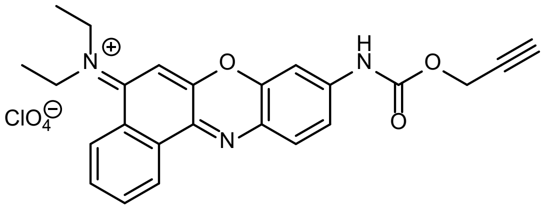

Nile Blue perchlorate (75 mg, 0.18 mmol, 1 eq) and NaH 60% in mineral oil (30 mg, 0.54 mmol, 3 eq) were placed under N_2 atmosphere, then dry THF (3 mL) was added, and the mixture was allowed to stir at r.t. for 30 min. The reaction was cooled to 0°C and a solution of propargyl chloroformate (53 mL, 0.54 mmol, 3 eq) in dry THF (3 mL) was added via a syringe. The reaction was allowed to warm to r.t. for 30 min, diluted with MeCN:H_2O and freeze dried to give a dark residue that was triturated with Et_2O to give compound **16b** as a blue solid (90 mg, quantitative yield).

^1H NMR (500 MHz, DMSO-d_6) δ 8.58 (dd, J = 8.0, 0.6 Hz, 1H), 8.41 – 8.29 (m, 1H), 7.88 – 7.77 (m, 1H), 7.76 – 7.70 (m, 1H), 7.62 (d, J = 9.1 Hz, 1H), 6.86 (dd, J = 9.1, 2.7 Hz, 1H), 6.65 (d, J = 2.6 Hz, 1H), 6.56 (s, 1H), 4.87 (d, J = 2.4 Hz, 2H), 3.63 (t, J = 2.4 Hz, 1H), 3.52 (q, J = 7.0 Hz, 4H), 1.17 (t, J = 7.1 Hz, 6H); ^{13}C NMR (125 MHz, DMSO-d_6) δ 162.9, 160.9, 151.4, 150, 146.9, 138.8, 131.9, 131.8, 131.4, 130.7, 130.4, 125.4, 125.3, 123.9, 111.1, 99.3, 96.5, 79.4, 78.2, 53.7, 45.0, 12.9; HRMS (m/z, ESI): calcd. for $C_{24}H_{22}N_3O_3$ $[M]^+$: 400.1656; found 400.1644.

Compound 17a

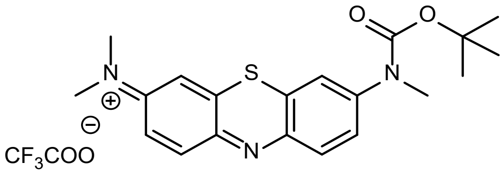

Azure B (150 mg, 0.49 mmol, 1 eq) and Cs_2CO_3 (479 mg, 1.47 mmol, 3 eq) were placed under N2 atmosphere, then dry MeCN (4 mL) was added, and the mixture was allowed to stir at r.t. for 1 h. The reaction was cooled to 0°C and a solution of Boc_2O (321 mg, 1.47 mmol, 3 eq) in dry MeCN (3 mL) was added via a syringe. The reaction was heated to 70 °C for 30 min, filtered and purified by preparative HPLC (H_2O:MeCN, 95:5 to 5:95 with 0.1% TFA) to render compound **17a** as a blue solid (6 mg, 4%).

^1H NMR (500 MHz, MeOD) δ 8.35 – 7.65 (m, 6H), 3.92 – 3.34 (m, 9H), 1.56 (s, 9H); HRMS (m/z, ESI): calcd. for $C_{20}H_{24}N_3O_2S$ $[M]^+$: 370.1584; found: 370.1591.

Compound 17b

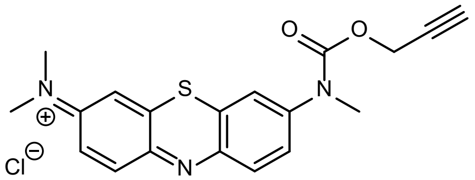

Azure B (150 mg, 0.49 mmol, 1 eq) and Cs_2CO_3 (479 mg, 1.47 mmol, 3 eq) were placed under N_2 atmosphere, then dry MeCN (4 mL) was added, and the mixture was allowed to stir at r.t. for 1 h. The reaction was cooled to 0°C and a solution of propargyl chloroformate (147 mL, 1.47 mmol, 3 eq) in dry MeCN (3 mL) was added via a syringe. Reaction was allowed to warm to r.t. for 1 h, solvents were removed under reduced pressure and the crude was purified by column chromatography (DCM:MeOH, 8:2) to render compound **17b** as a blue solid (31 mg, 18%).

^{1}H NMR (500 MHz, MeOD) δ 8.30 – 7.64 (m, 6H), 4.89 (s, 2H), 3.92 – 3.43 (m, 9H), 3.04 (t, J = 2.4 Hz, 1H); HRMS (m/z, ESI): calcd. for $C_{19}H_{18}N_3O_2S$ $[M]^+$: 352.1115; found: 352.1125.

Experimental Procedures

Synthesis of metal-based resins. Pd resins[1] and Au resins[2] were prepared as previously described.

Synthesis of AuPd resins. Dendritic Au-Pd alloy nanoparticles (AuPd-NPs) were synthesized via the co-reduction of $HAuCl_4$ and H_2PdCl_4 using ascorbic acid in the presence of pluronic P123, employing a manual dropping addition procedure. H_2PdCl_4 solution (0.01 M) was prepared by dissolving 0.089 g of $PdCl_2$ powder in 50 mL of 0.02 M HCl solution at 60°C. 5 mL of an aqueous solution containing 50.6 mg of Pluronic P123 and 88.0 mg of ascorbic acid was added into a 15 mL vial and mixed uniformly under continuous stirring at 25°C. Subsequently, 5 mL of an aqueous solution containing 6.25 µmol of $HAuCl_4$ and 6.25 µmol of H_2PdCl_4 was added into the vial slowly dropwise. Afterwards, the mixture was stirred at 25 °C for another 1 h to yield a dark brown solution. The total concentration of the metal precursors in the final reaction solution was 1.25 mM with an Au:Pd ratio of 1:1, and the molar ratio in the synthesis recipe was metal ion/ascorbic acid/P123/H_2O = 1/40/0.7/44800. The obtained AuPd-NPs were collected by centrifugation at 12,782 × g for 20 min and washed three times with ultrapure H_2O. Then the pellet was redispersed into water for further use. Next, the AuPd resins were prepared by deposition and electrostatic trapping of AuPd-NPs on the surface of a mono-functionalized polyethylene glycol (PEG)-grafted low-cross-linked polystyrene resins (TentaGel®). 60 mg of amino-functionalized TentaGel® HL resins (75 microns) were mixed with 10 mL of the prepared solution of AuPd-NPs. The suspension was stirred at 20°C for 12 h in the dark using an IKA rotary shaker loopster digital. Subsequently, the product was collected by centrifugation (5 min at 10.226 × g), washed several times with H_2O, dried, and stored at 4 °C.

Electron microscopy analyses. Scanning transmission electron microscopy (STEM) and energy dispersive X-ray spectroscopy (EDS) measurements have been performed using a ThermoFisher Scientific Titan Low-Base probe-corrected microscope. The analyses were conducted at 300 kV in the Titan Low-Base using also high-angle annular dark field (HAADF)-STEM imaging at the LMA-INMA-UNIZAR facilities with the assistance of Dr Fernandez-Pacheco. SEM images were obtained using a FEI Inspect F50 microscope equipped with an EDS analytical system and 10-20kV of acceleration voltage. AuPd resins were first infiltrated and embedded in a EMBed812 resin polymerizing at 60 °C for 24 h. Then, before curing the resin block, it was stained with OsO4 (4 wt.% in H_2O) for 1 h to harden the AuPd resins and ease sectioning. Then, semithin and ultrathin sections of the AuPd resins were

obtained with a diamond knife (ultra 35°, Diatome) using an Ultramicrotome (Leica EM UC7). For characterization by SEM, the semithin sections (500-1000 nm in thickness) were placed on a pin stub with carbon tape and coated with 15 nm of carbon. Previously, stubs were glow discharged (30 s, 15 mA) to enable the sections to be mounted as flat as possible. AuPd resins were also mounted on stubs and coated with 15 nm of carbon to be observed by SEM. The ultrathin sections (50-70 nm in thickness) were placed on a carbon film on copper grid (150 mesh) and allowed to dry in air.

Computational modelling. Time dependent density functional theory (TD-DFT) was used to calculate electronic transitions for compound 1 and compound 2. These calculations were performed in Gaussian 16 Rev. B.01 using WB97XD functional with the cc-pVTZ basis set. Geometry optimization was enhanced by the inclusion of solvent effects modelled through the Solvation Model based on Density (SMD) within the Polarizable Continuum Model (PCM) framework for ethanol. The TD-DFT parameters specified the examination of 10 states, focusing on the first excited state (root=1).

2. Supplementary Figures

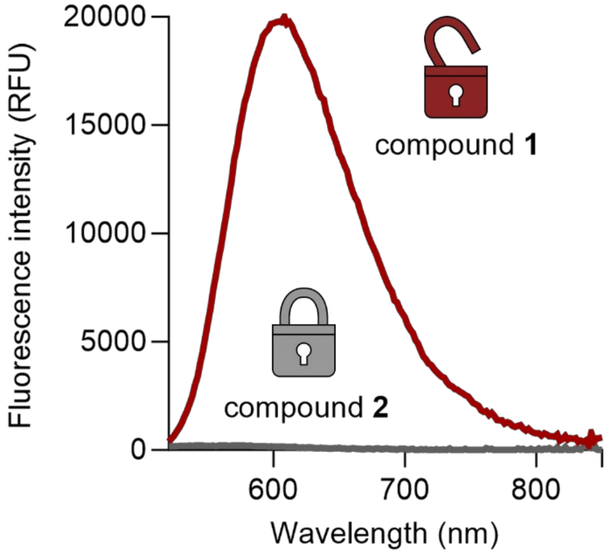

Supplementary Figure 1. **Fluorescence spectra of compounds 1 and 2**. Uncaged nitrobenzoselenadiazole (**1**, 200 μM) and caged nitrobenzoselenadiazole (**2**, 200 μM) were dissolved in EtOH and representative spectra were acquired after excitation at 490 nm.

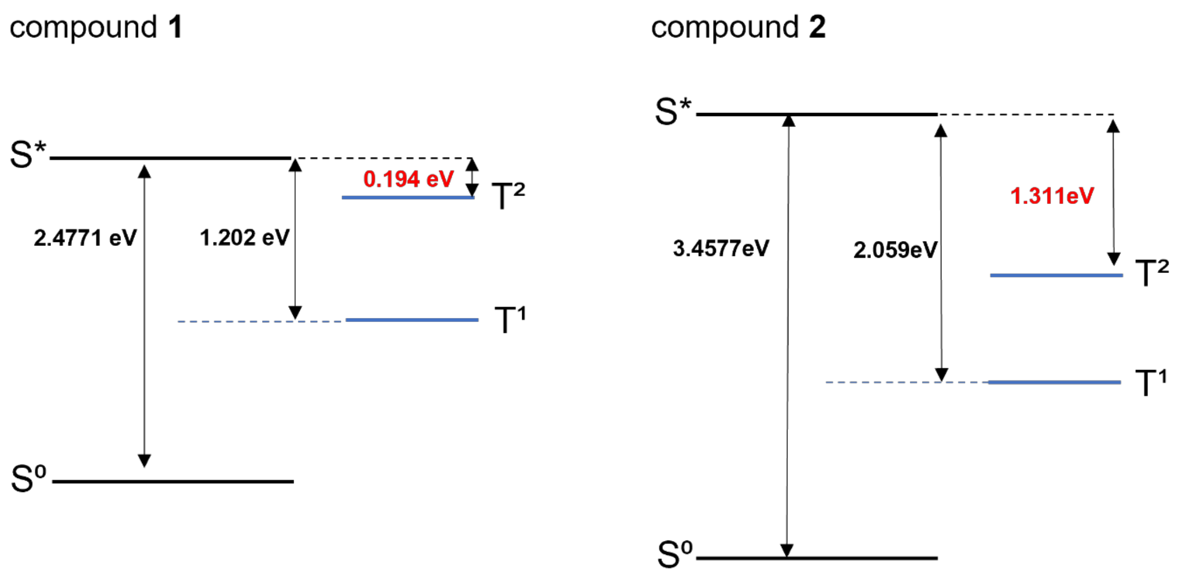

Supplementary Figure 2. Energy levels for the transitions associated with compounds 1 and 2. Jablonski diagrams illustrating the energy levels of ground (S^o), singlet excited (S^*) and triplet excited (T^2) states for each compound.

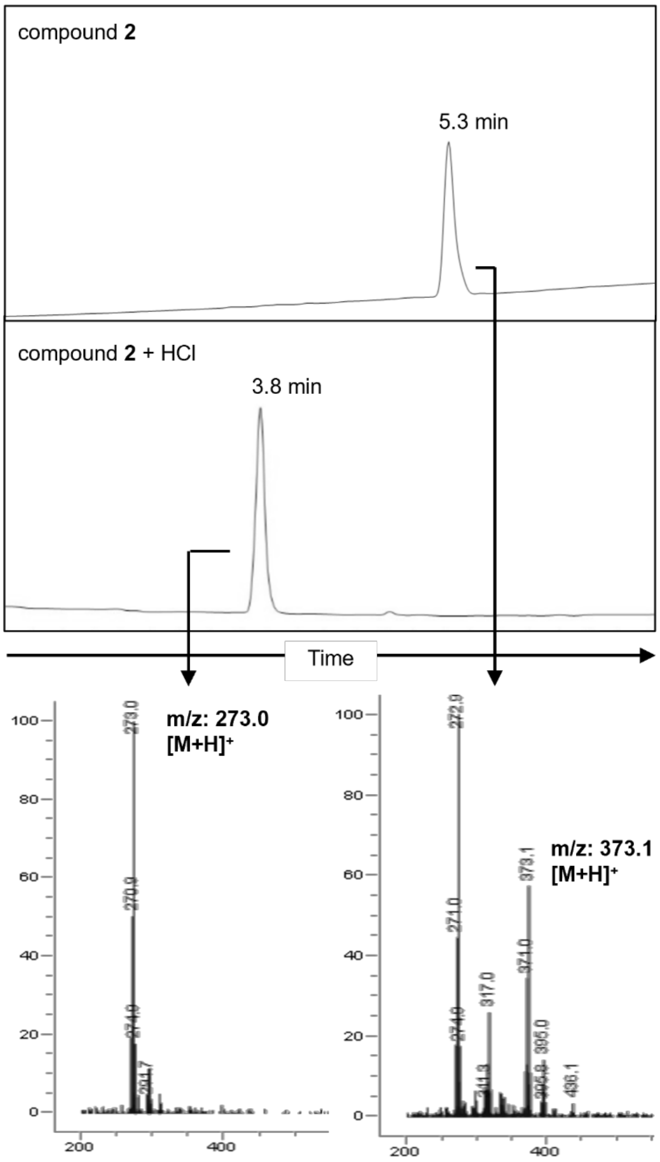

Supplementary Figure 3. HPLC analysis of uncaging of compound 2. Representative HPLC traces (UV detection: 254 nm) and MS spectra of compound **2** (400 μM) before and after reaction with 4 N HCl (10 min). Calculated m/z values: 272.9 (compound **2**), 373.0 (compound **1**).

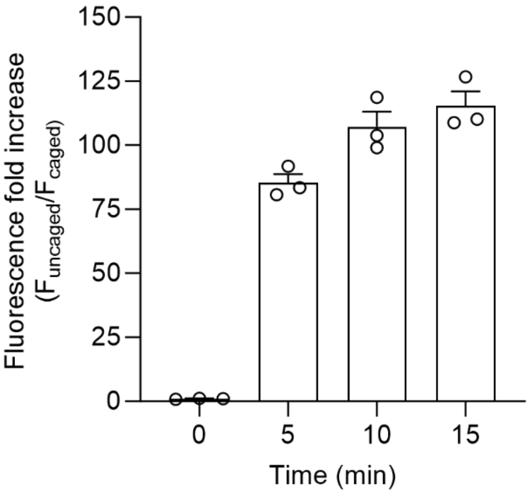

Supplementary Figure 4. Time-course fluorescence analysis of compound 2. Fluorescence fold increase (exc/em: 490/620 nm) of compound **2** (50 μM) in the presence of concentrated HCl. Values presented as means and error bars as SEM (n=3, independent experiments). Source data are provided as a Source Data file.

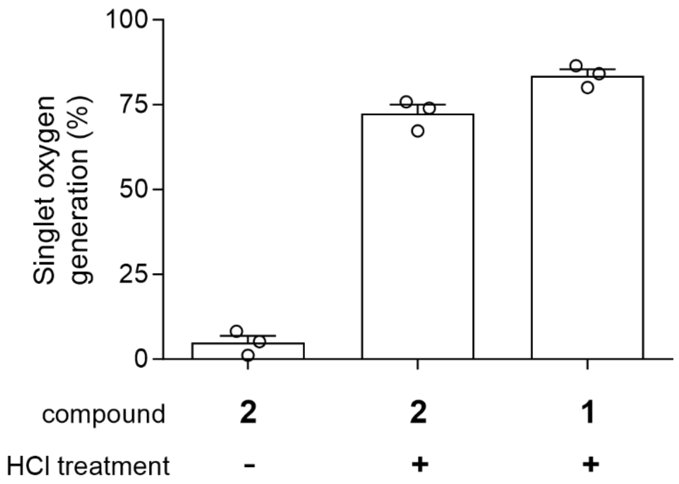

Supplementary Figure 5. Singlet oxygen generation of compound 2. Singlet oxygen generation of caged compound **2** (50 µM) measured as percentages of DPBF (300 µM in EtOH) absorbance decrease before and after reaction with concentrated HCl (4N in dioxane) followed by illumination at 520 nm (0.45-0.5 mW cm^{-2}, 6 min). Uncaged compound **1** under the same experimental conditions was used as a positive control. Values presented as means and error bars as SEM (n=3, independent experiments). Source data are provided as a Source Data file.

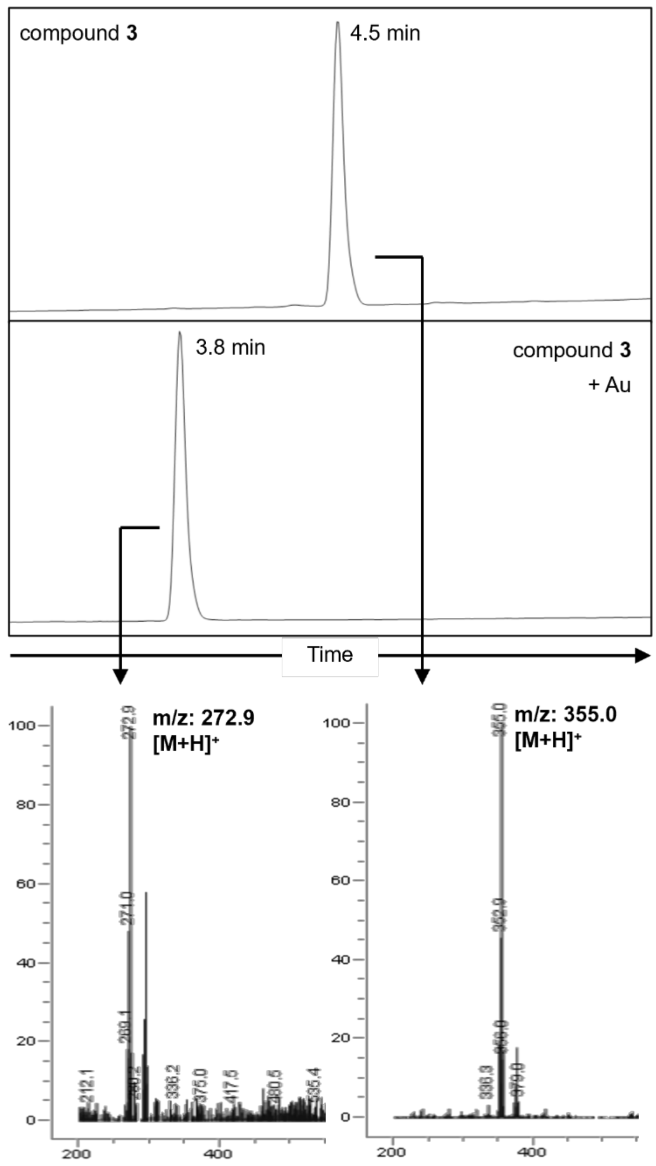

Supplementary Figure 6. HPLC analysis of uncaging of compound 3. Representative HPLC traces (UV detection: 254 nm) and MS spectra of compound **3** (500 μM) before and after reaction with Au beads (1 mg mL^{-1},18 h). Calculated m/z values: 355.0 (compound **3**), 273.0 (compound **1**).

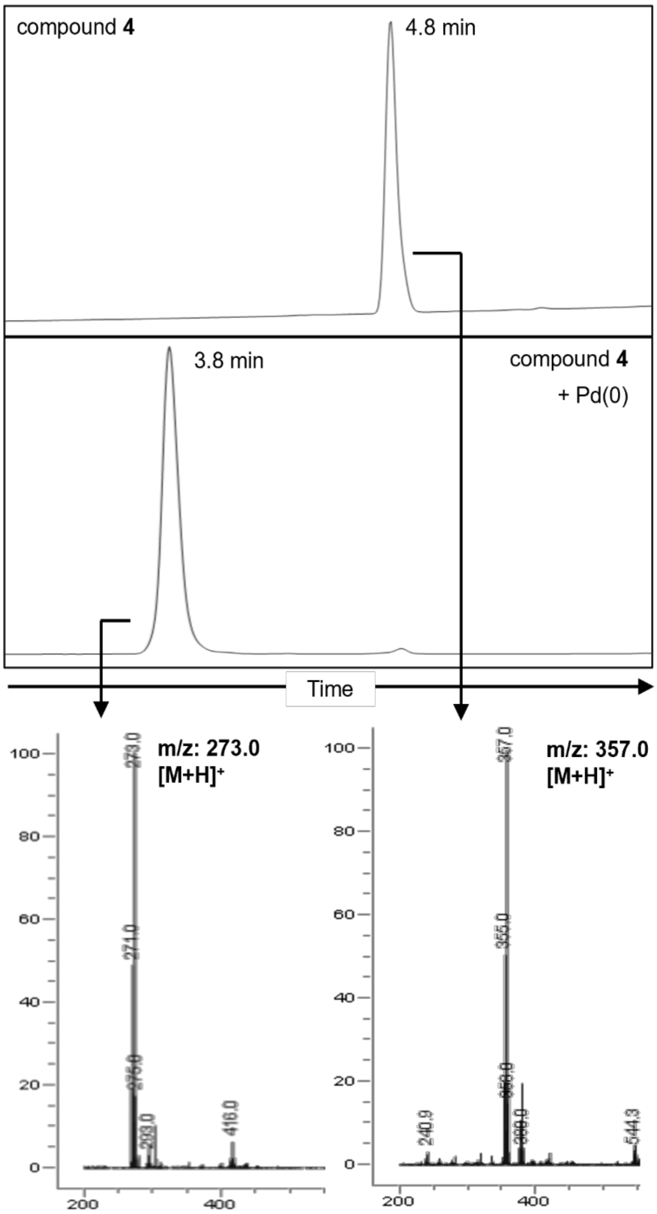

Supplementary Figure 7. HPLC analysis of uncaging of compound 4. Representative HPLC traces (UV detection: 254 nm) and MS spectra of compound **4** (50 µM) before and after reaction with $Pd(PPh_3)_4$ (25 µM, 7 h). Calculated m/z values: 357.0 (compound **4**), 273.0 (compound **1**).

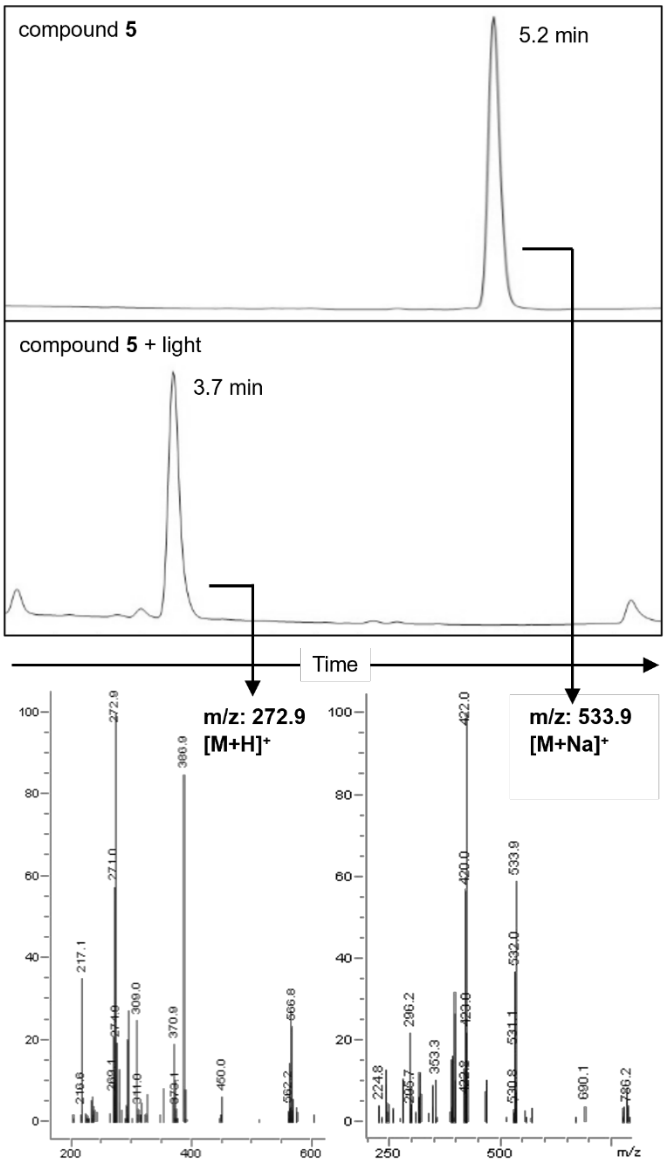

Supplementary Figure 8. HPLC analysis of uncaging of compound 5. Representative HPLC traces (UV detection: 254 nm) and MS spectra of compound **5** (50 μM) before and after UV light illumination (365 nm, 1 mW, 30 min). Calculated m/z values: 534.0 (compound **5**), 273.0 (compound **1**).

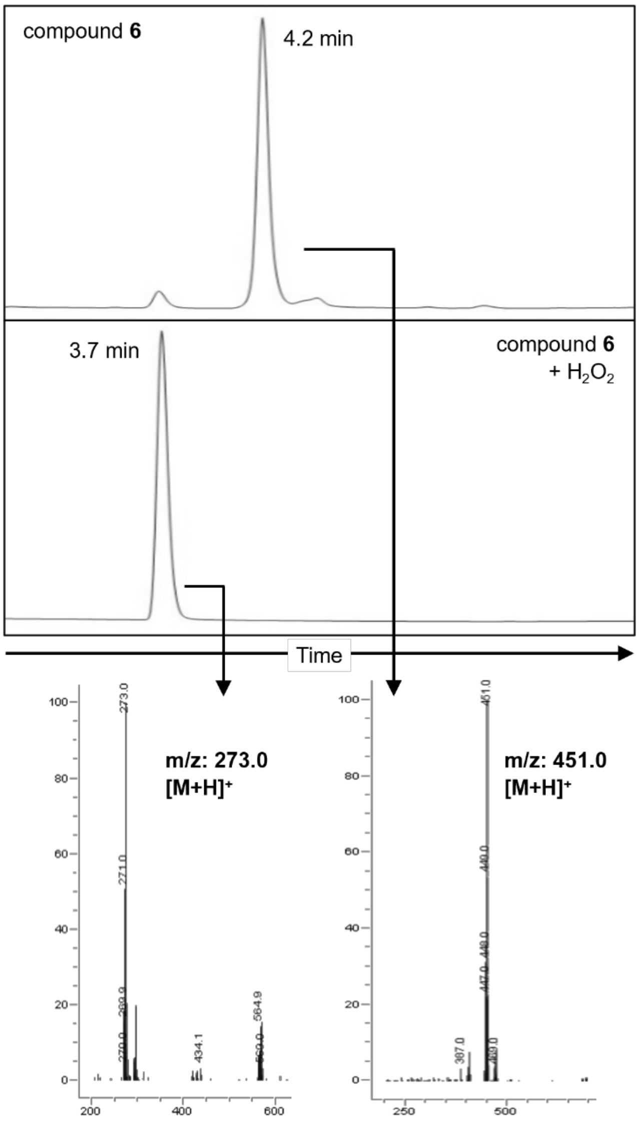

Supplementary Figure 9. HPLC analysis of uncaging of compound 6. Representative HPLC traces (UV detection: 254 nm) and MS spectra of compound **6** (50 μM) before and after reaction with H_2O_2 (12 mM, 10 min). Calculated m/z values: 451.0 (compound **6**), 273.0 (compound **1**).

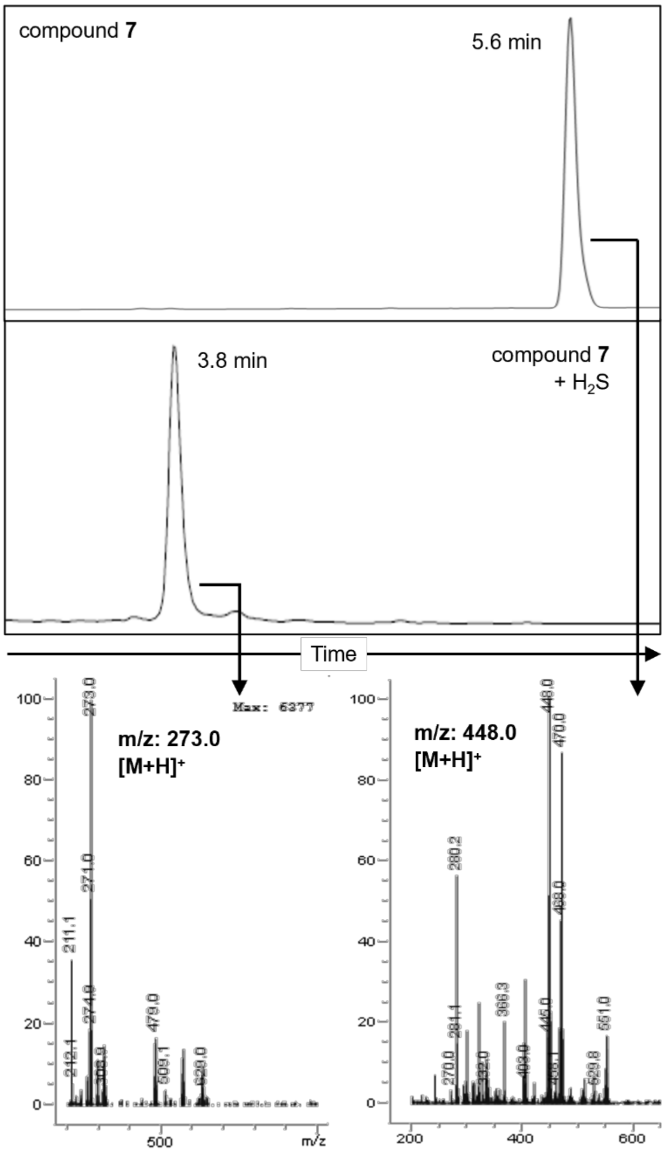

Supplementary Figure 10. HPLC analysis of uncaging of compound 7. Representative HPLC traces (UV detection: 254 nm) and MS spectra of compound **7** (250 μM) before and after reaction with H_2S (generated in situ by reaction between 50 mM Na_2S and 500 mM HCl in H_2O, 5 min).

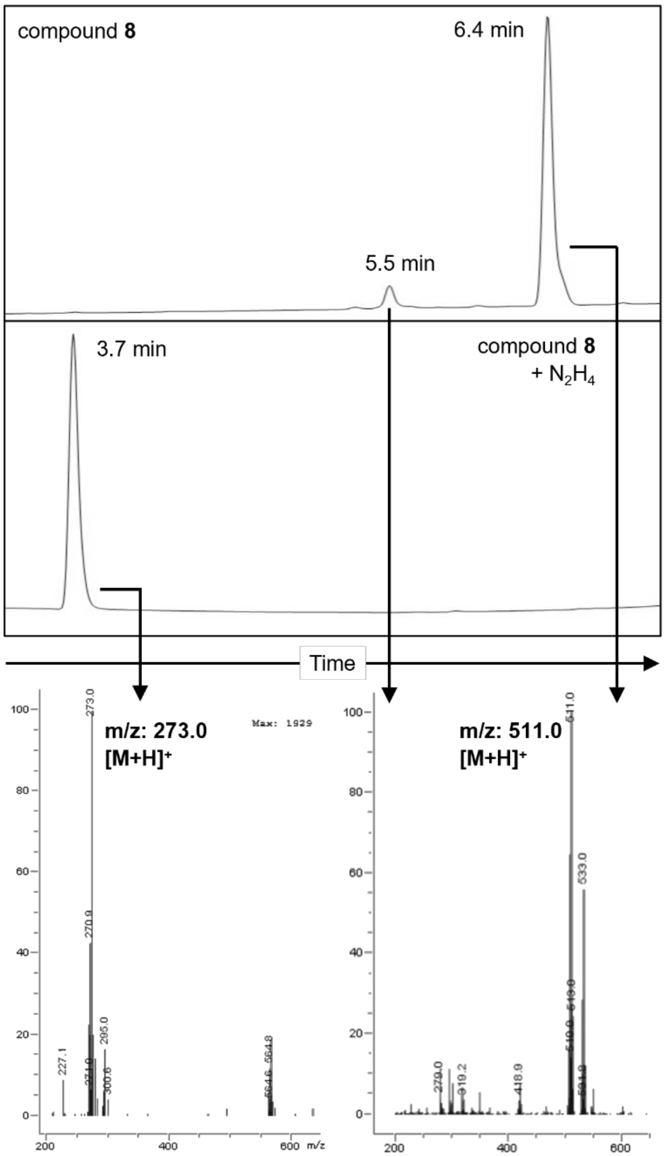

Supplementary Figure 11. HPLC analysis of uncaging of compound 8. Representative HPLC traces (UV detection: 254 nm) and MS spectra of compound **8** (50 μM) before and after reaction with N_2H_4 (10 mM, 4 h). Calculated m/z values: 511.0 (compound **8**), 273.0 (compound **1**).

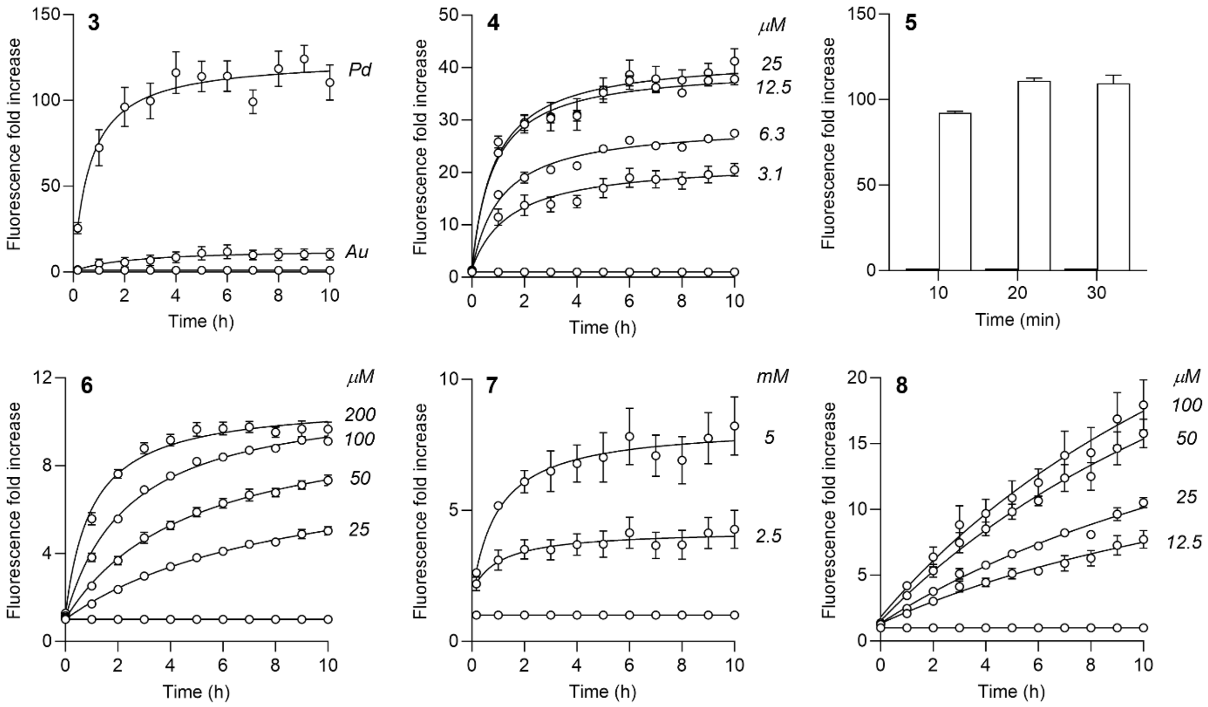

Supplementary Figure 12. Time-course fluorescence analysis of compounds 3-8. Fluorescence fold increase (exc/em: 490/620 nm) of compounds **3**-**8** (25 μM) in the presence of biorthogonal and bioresponsive triggers: compound **3**: no resin, Au-resin 1 mg mL^{-1} and Pd-resin 1 mg mL^{-1}; compound **4**: 0, 3.125. 6.25, 12.5 and 25 μM $Pd(PPh_3)_4$; compound **5**: 365 nm illumination at 1 mW cm^{-2}; compound **6**: 0, 25, 50, 100 and 200 μM H_2O_2; compound **7**: 0. 2.5 and 5 mM H_2S; compound **8**: 0, 12.5, 25, 50 and 100 μM N_2H_4. Values presented as means and error bars as SEM (n=5, independent experiments). Source data are provided as a Source Data file.

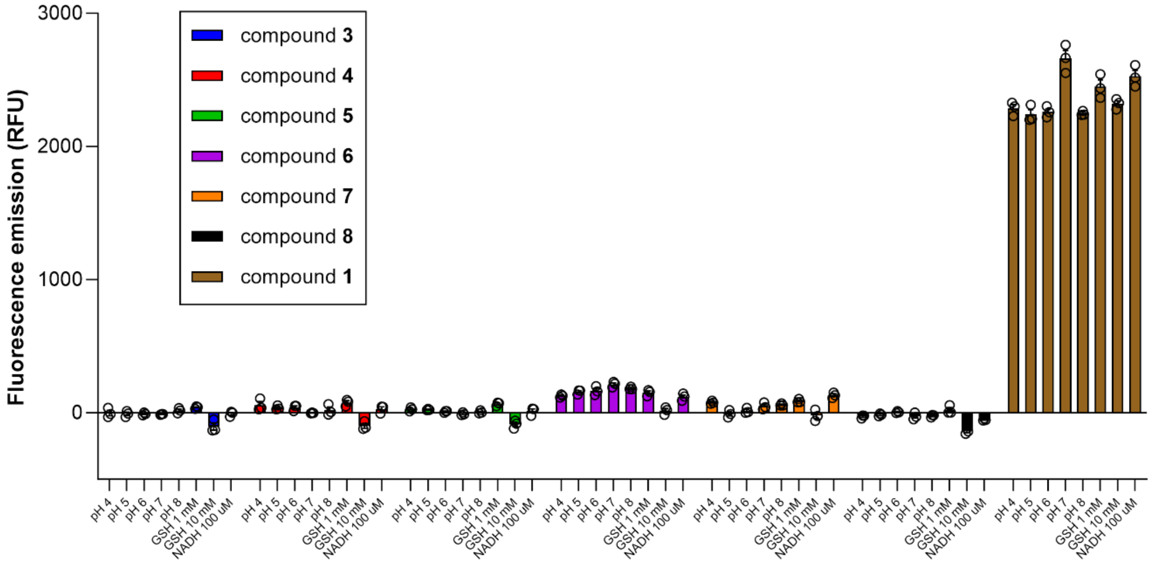

Supplementary Figure 13. Stability study of compounds 3-8. Fluorescence emission of activatable PS (compounds **3**-**8**) after incubation with different pHs and bioanalytes (exc: 490 nm/em: 580 nm). Compounds **3**-**8** (200 µM) were incubated in aqueous solutions of different pH and with glutathione (1 mM and 10 mM) and NADH (100 µM) for 2 h at r.t. Compound **1** (200 µM) was used as a reference of uncaged PS. Values presented as means±SEM (n=3, independent experiments). Source data are provided as a Source Data file.

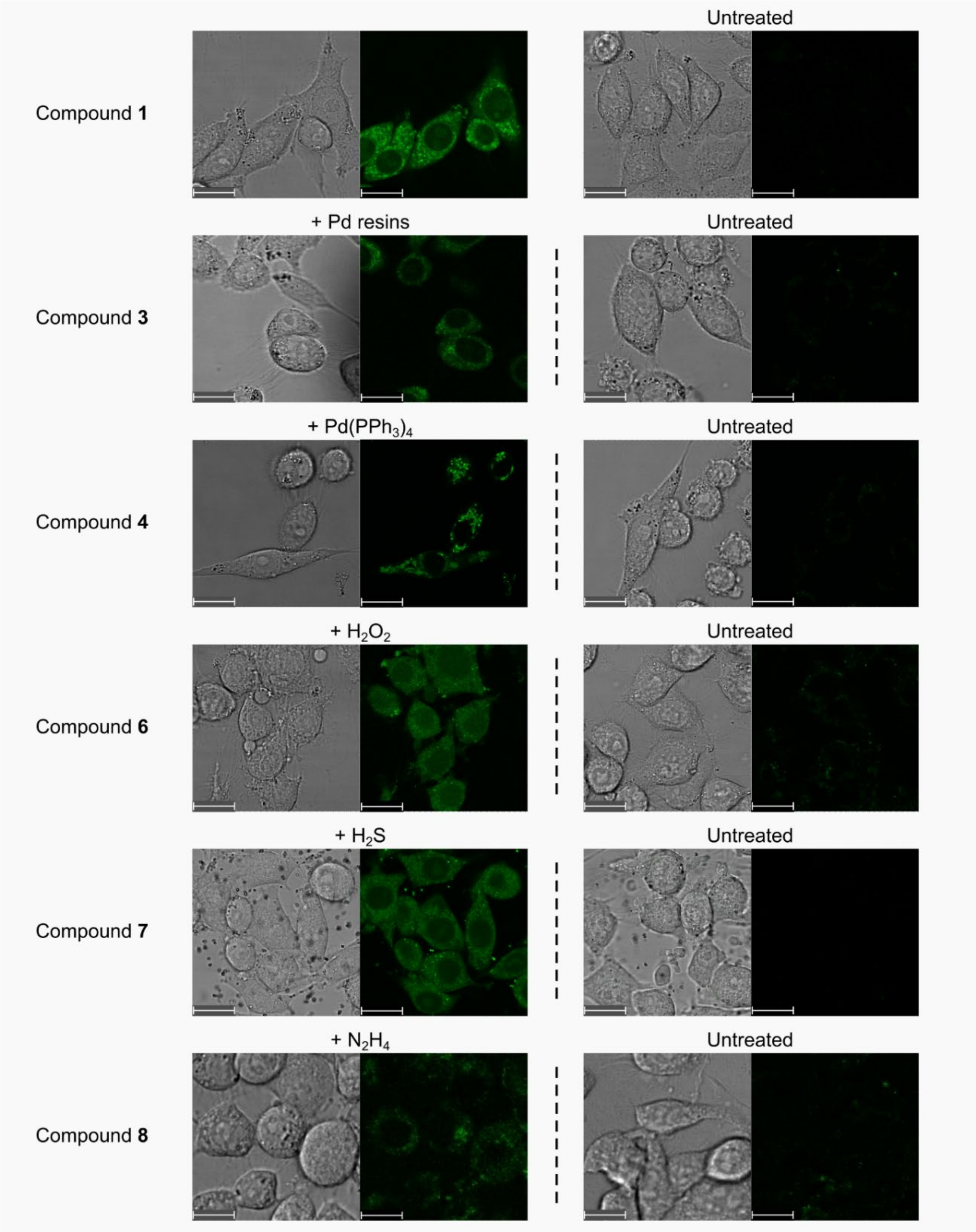

Supplementary Figure 14. Representative confocal microscopy images of MCF-7 cells after incubation with nitrobenzoselenadiazoles. Caged compounds **3-8** (50 μM) were uncaged with their respective triggers -as indicated in the figure- and the resulting mixtures were incubated with MCF-7 cells for 30 min and imaged under a confocal microscope (exc/em: 488/620 nm). Cells were also incubated with caged compounds **3-8** (50 μM) without any triggers as negative controls (images labeled as untreated). Compound **1** (50 μM) was used as a positive control. Scale bars: 18 μm.

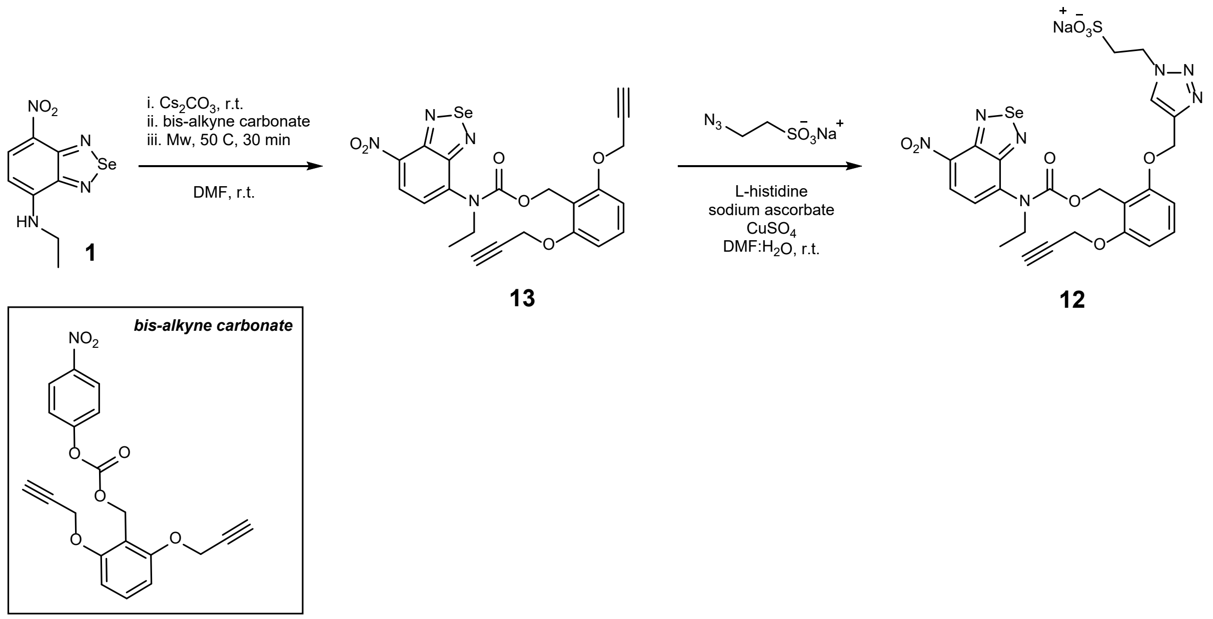

Supplementary Figure 15. Synthetic scheme for the preparation of the cell-impermeable caged PS **12**.

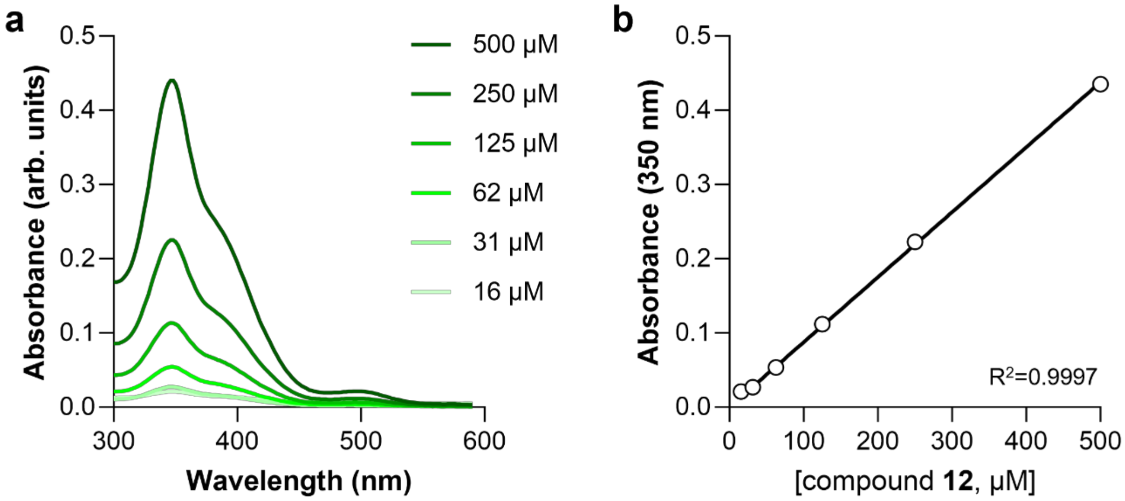

Supplementary Figure 16. Solubility analysis of compound 12 in water. a) Absorbance spectra in H_2O (blank subtracted) at increasing concentrations of compound **12**. b) Linear regression analysis of the absorbance (350 nm) at different concentrations of compound **12**.

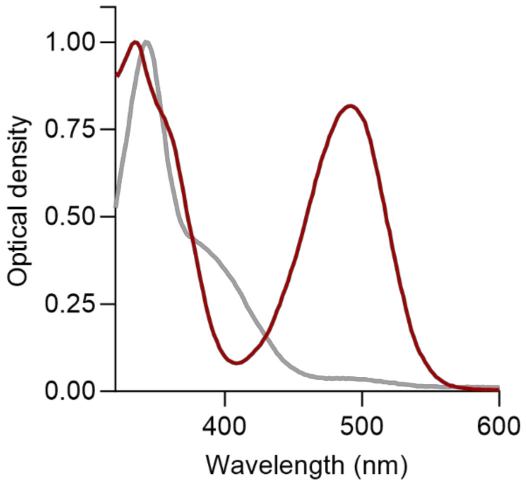

Supplementary Figure 17. Normalized absorbance spectra of compound 12. Compound **1** (red line) and compound **12** (grey line) were dissolved at 200 µM in EtOH and their absorbance spectra were recorded.

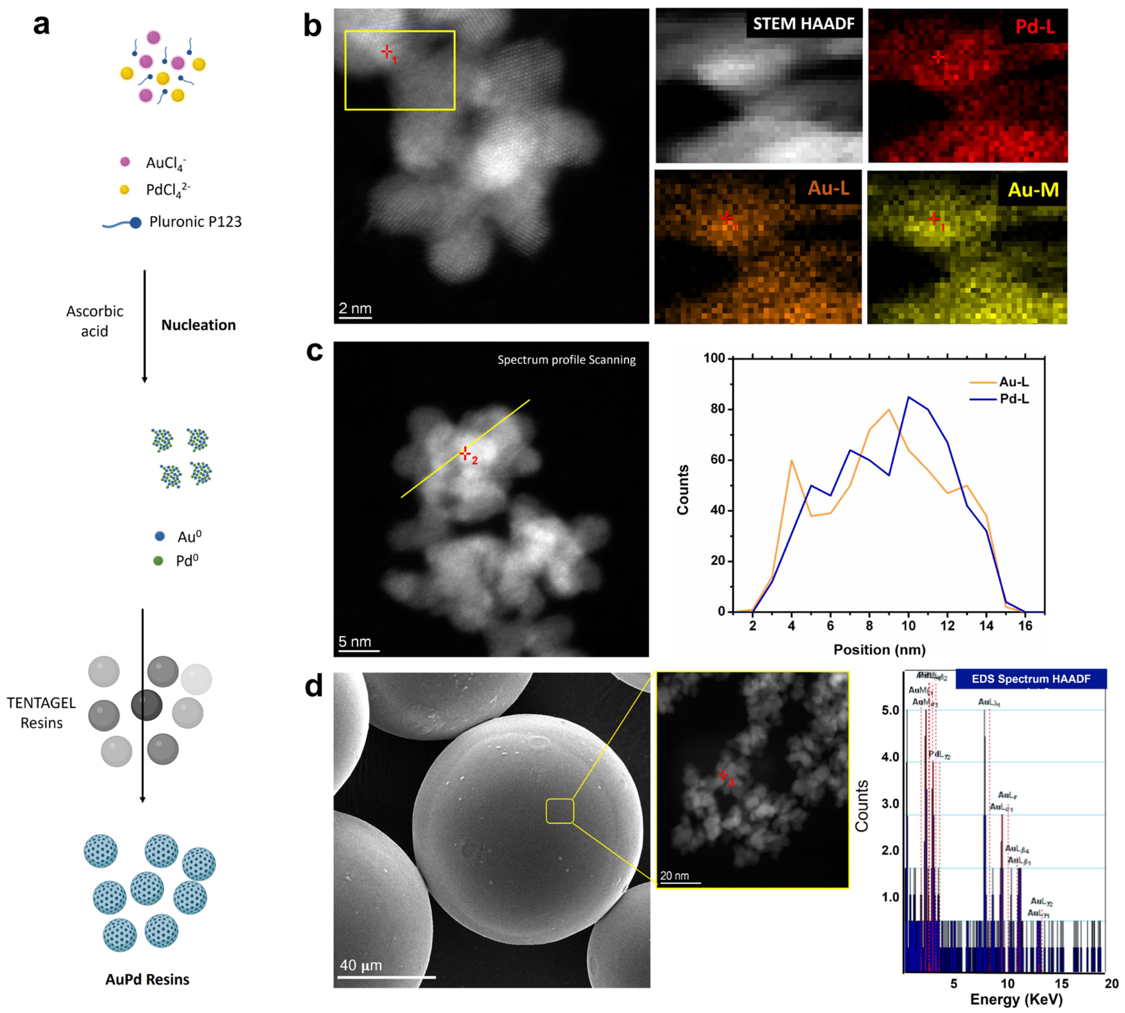

Supplementary Figure 18. Synthesis and characterization of AuPd resins. a) Preparation of dendritic AuPd NPs and AuPd resins. b) Representative HAADF-STEM images of AuPd NPs and elemental maps of Pd (L) and Au (L and M electron energy levels) of the highlighted (yellow) area. Merged image shows alloy formation. c) Line scanning elemental profiles recorded along the yellow line as marked in the AuPd NPs. d) SEM (left) and STEM-EDS (middle) images of ultramicrotome cross-sections of AuPd resins and EDS spectra (right) of the highlighted area.

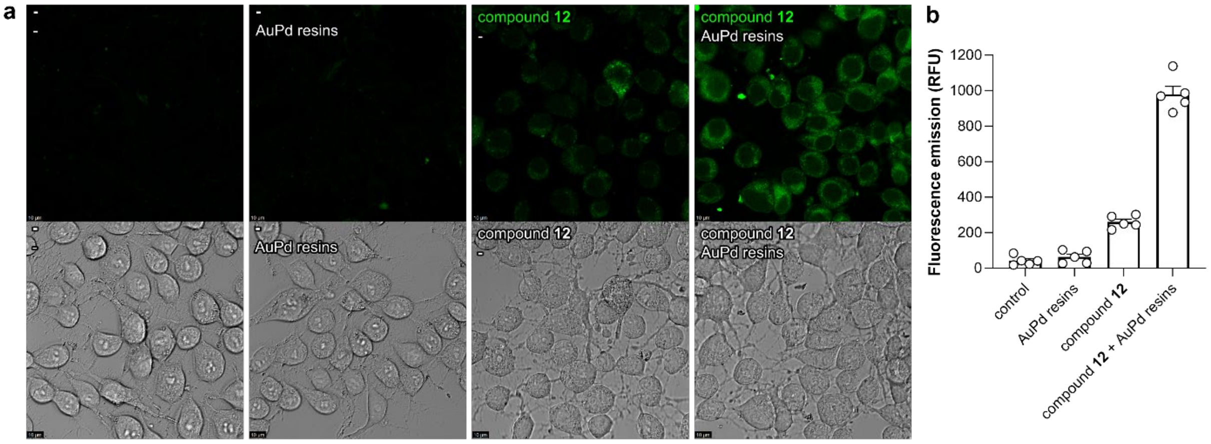

Supplementary Figure 19. Representative confocal microscopy images of MCF-7 cells after incubation with compound 12. a) Cells were incubated with compound **12** (400 μM) in the absence or presence of AuPd resins (1 mg mL^{-1}) in supplemented media and imaged under a confocal microscope (exc/em: 488/620 nm). Representative fluorescence (top panels) and brightfield (bottom panels) are shown for each of the experimental conditions. Scale bars: 10 mm. b) Quantification of mean cell fluorescence was performed using with FIJI. Values presented as individual replicates and bars as means±SEM (n=5, independent experiments). Source data (b) are provided as a Source Data file.

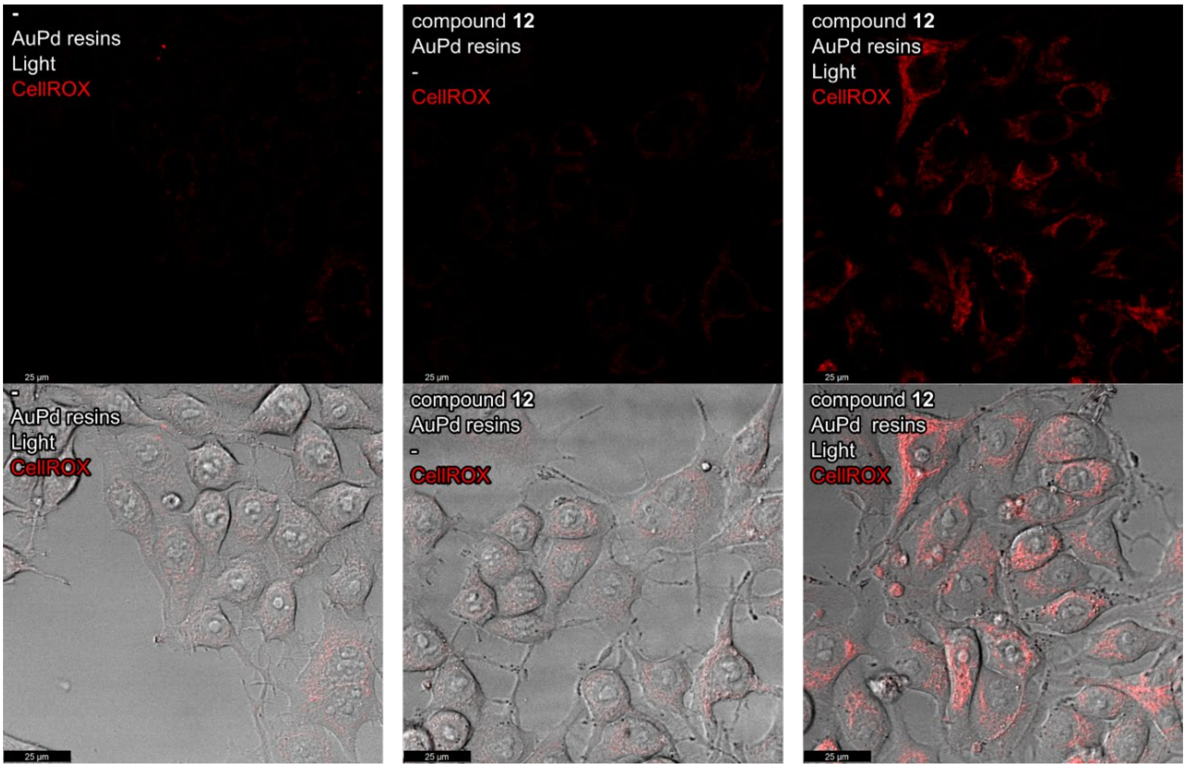

Supplementary Figure 20. ROS production in MCF-7 cells after incubation with compound 12 and light illumination. Cells were incubated in different treatments combining compound **12** (400 µM) and AuPd resins (1 mg mL^{-1}) followed by white light illumination under a UV filter (10 mW cm^{-2}, 60 min). CellROX^{TM} Deep Red (5 µM) was added, and ROS production was imaged under a confocal microscope (exc/em: 644/665 nm). Representative fluorescence (top panels) and merged fluorescence and brightfield images (bottom panels) are shown for each of the experimental conditions. Scale bars: 25 µm.

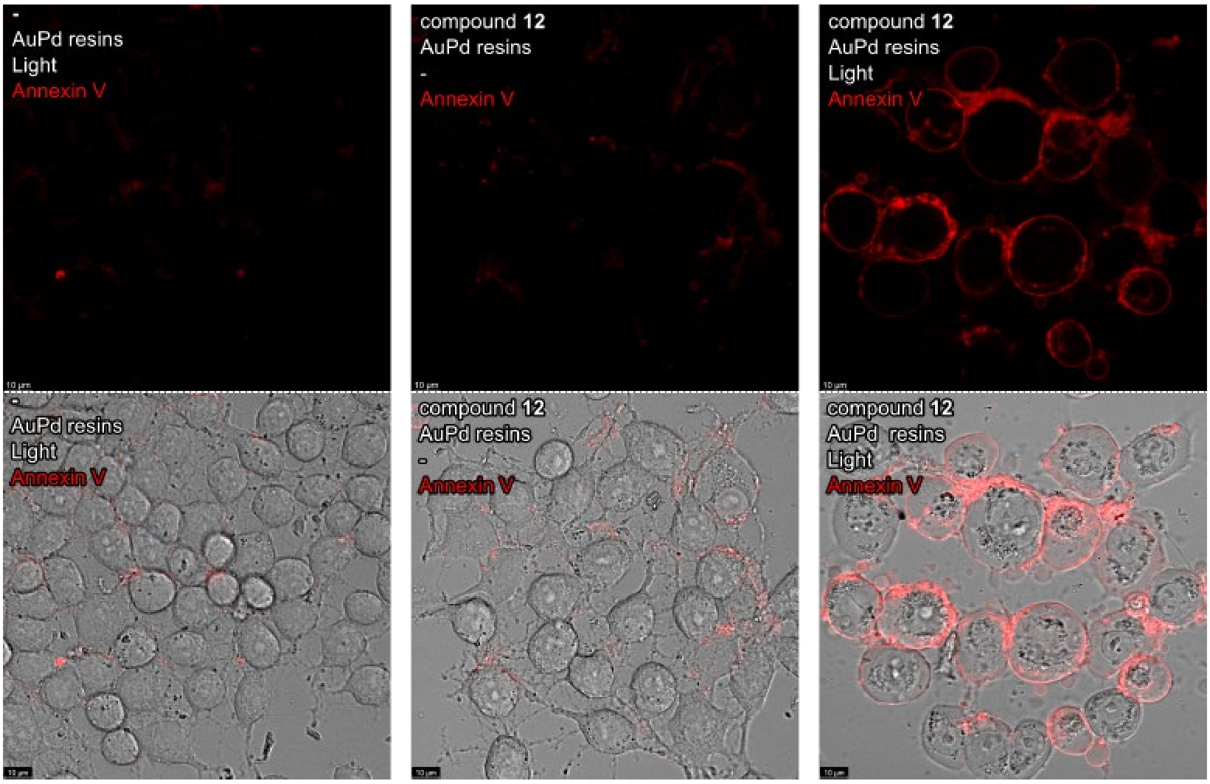

Supplementary Figure 21. Representative confocal microscopy images of MCF-7 cells after incubation with compound 12 and light illumination. Cells were incubated in different treatments combining compound **12** (400 μM) and AuPd resins (1 mg mL^{-1}) followed by white light illumination under a UV filter (10 mW cm^{-2}, 60 min). Annexin V-AF647 (1 μg mL^{-1}) was added as an apoptosis marker and cells were imaged under a confocal microscope (exc/em: 633/671 nm). Representative fluorescence (top panels) and merged fluorescence plus brightfield (bottom panels) are shown for each of the experimental conditions. Scale bars: 10 μm.

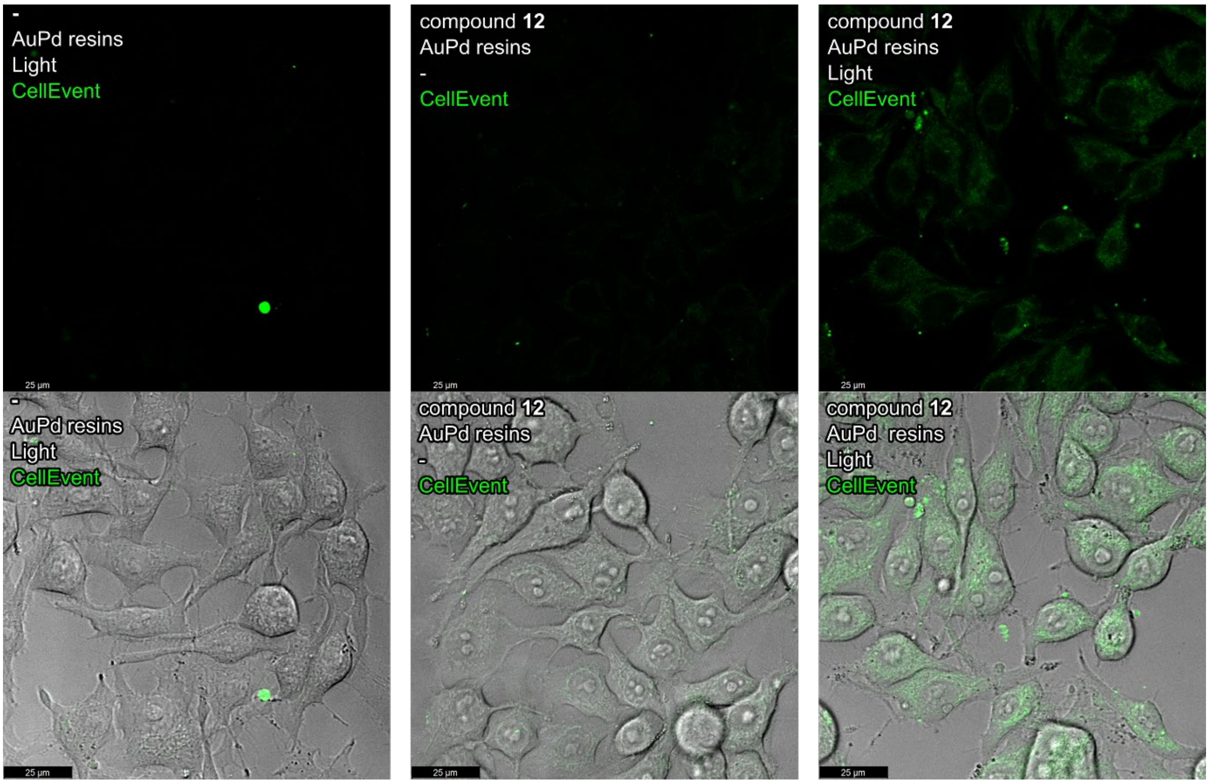

Supplementary Figure 22. Caspase activity in MCF-7 cells after incubation with compound 12 and light illumination. Cells were incubated in different treatments combining compound **12** (400 µM) and AuPd resins (1 mg mL^{-1}) followed by white light illumination under a UV filter (10 mW cm^{-2}, 60 min). CellEvent™ Caspase-3/7 (5 µM) was added, and cells were imaged under a confocal microscope (exc/em: 502/530 nm). Representative fluorescence (top panels) and merged fluorescence and brightfield (bottom panels) are shown for each of the experimental conditions. Scale bars: 25 µm.

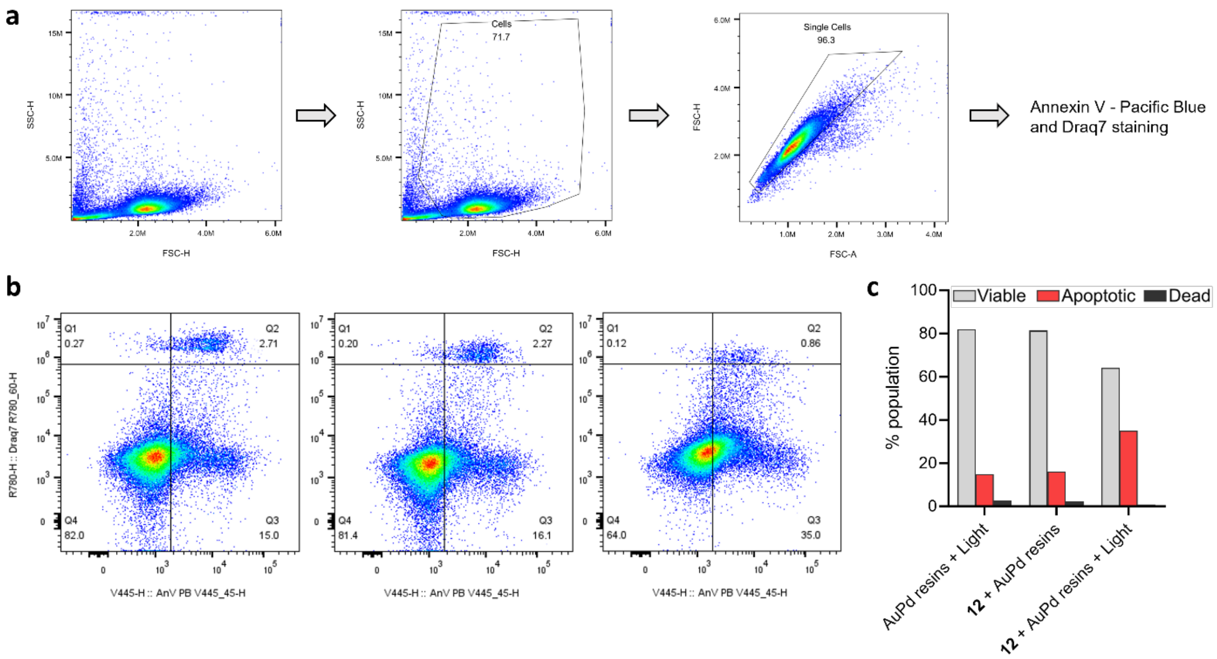

Supplementary Figure 23. Analysis of cell viability and apoptosis using flow cytometry. a) Flow gating strategy. b) Representative density plots from flow cytometry analysis showing the distribution of cell populations based on Annexin V – Pacific Blue (x-axis) and DRAQ7 (y-axis) staining. The four quadrants (Q1, Q2, Q3, Q4) indicate different cell populations: Q1 (Annexin V^{-}/DRAQ7^{+}, necrotic cells), Q2 (Annexin V^{+}/ DRAQ7^{+}, late apoptotic cells), Q3 (Annexin V^{+}/DRAQ7^{-}, early apoptotic cells), and Q4 (Annexin V^{-}/ DRAQ7^{-}, viable cells). The plots correspond to three different treatments: AuPd resins + light (left), **12** + AuPd resins (middle), and **12** + AuPd resins + light (right). c) Percentages of viable (gray), apoptotic (red), and necrotic (black) cell populations across the three treatment conditions.

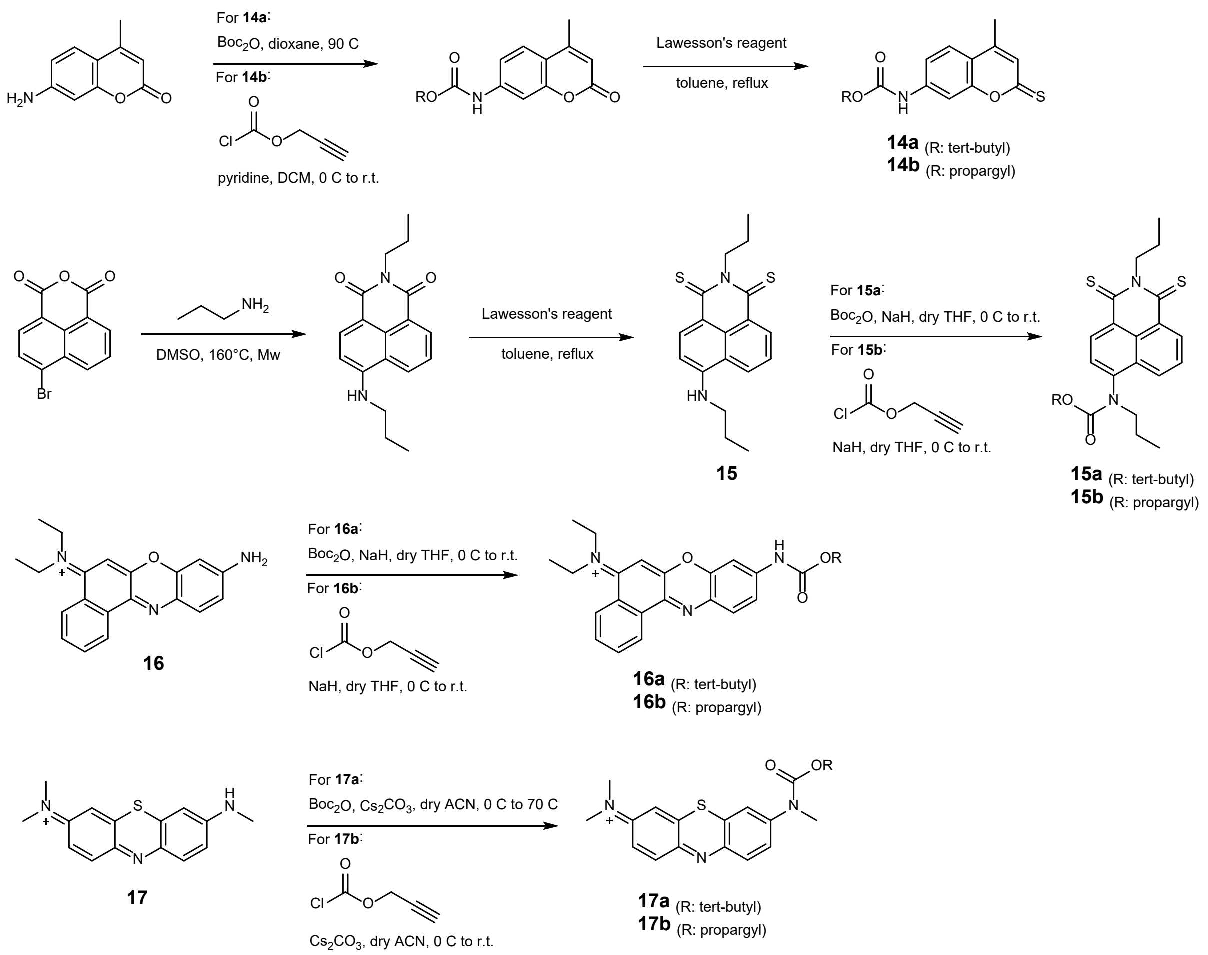

Supplementary Figure 24. Synthetic scheme for the preparation of UV-to-NIR caged PS. Synthesis of caged 2-thioxocoumarins (**14a** and **14b**), caged thionaphthalimides (**15a** and **15b**), caged benzophenoxazines (**16a** and **16b**) and caged phenothiazines (**17a** and **17b**).

	**14-17** (uncaged)	**14a-17a** (Boc protected)	**14b-17b** (Poc protected)	Stokes' shift (Boc)	Stokes' shift (Poc)
2-thioxocoumarin (**14**)	425	400	402	25	23
thionaphthalimide (**15**)	580	440	436	140	144
benzophenoxazine (**16**)	630	540	554	90	76
phenothiazine (**17**)	640	585	580	55	60

Supplementary Figure 25. Absorbance maxima wavelengths and Stokes' shifts for compounds 14-17, 14a-17a and 14b-17b. Compounds were dissolved in EtOH (200 μM) and absorbance spectra were recorded. All values are shown in nm.

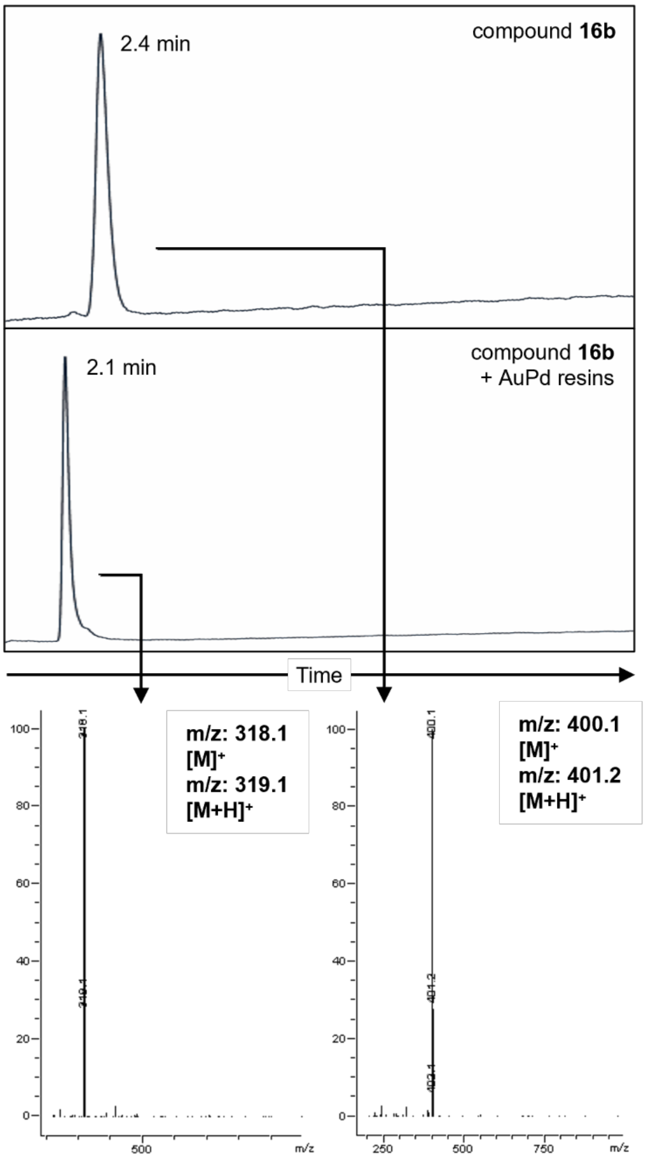

Supplementary Figure 26. HPLC analysis of uncaging of compound 16b. Representative HPLC traces (UV detection: 640 nm top panel, 560 nm bottom panel) and MS spectra of compound **16b** (1 μM) before and after reaction with AuPd resins (10 mg mL^{-1}, 17 h). Calculated m/z values: 400.2 (compound **16b**), 318.2 (compound **16**).

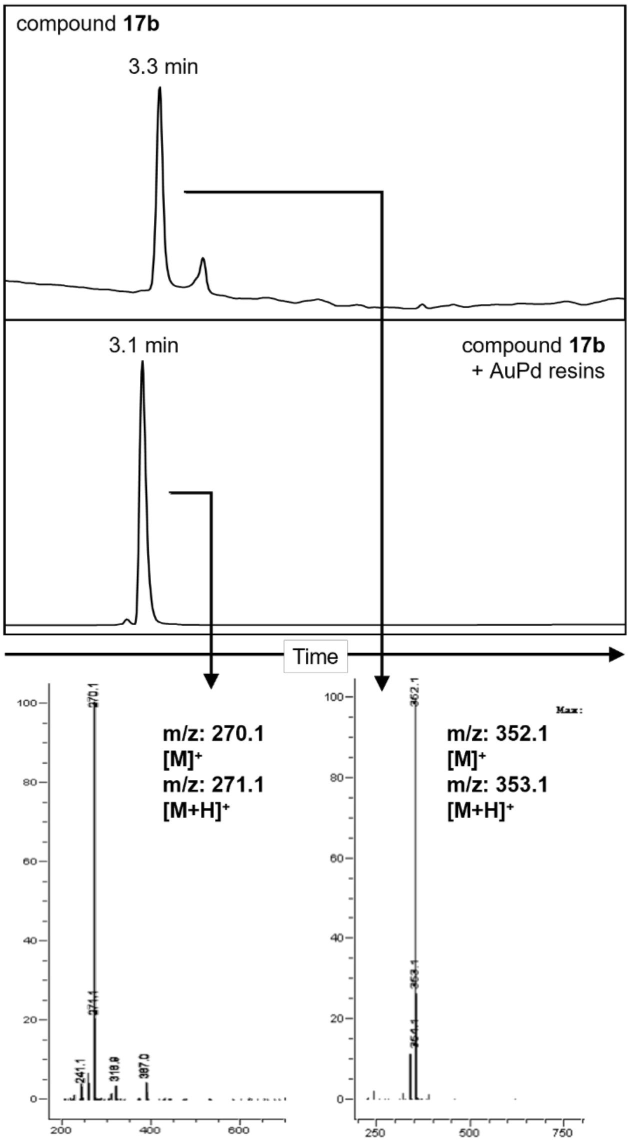

Supplementary Figure 27. HPLC analysis of uncaging of compound 17b. Representative HPLC traces (UV detection: 650 nm) and MS spectra of compound **17b** (300 μM) before and after reaction with AuPd resins (1 mg mL^{-1}, 17 h. Calculated m/z values: 352.1 (compound **17b**), 270.1 (compound **17**).

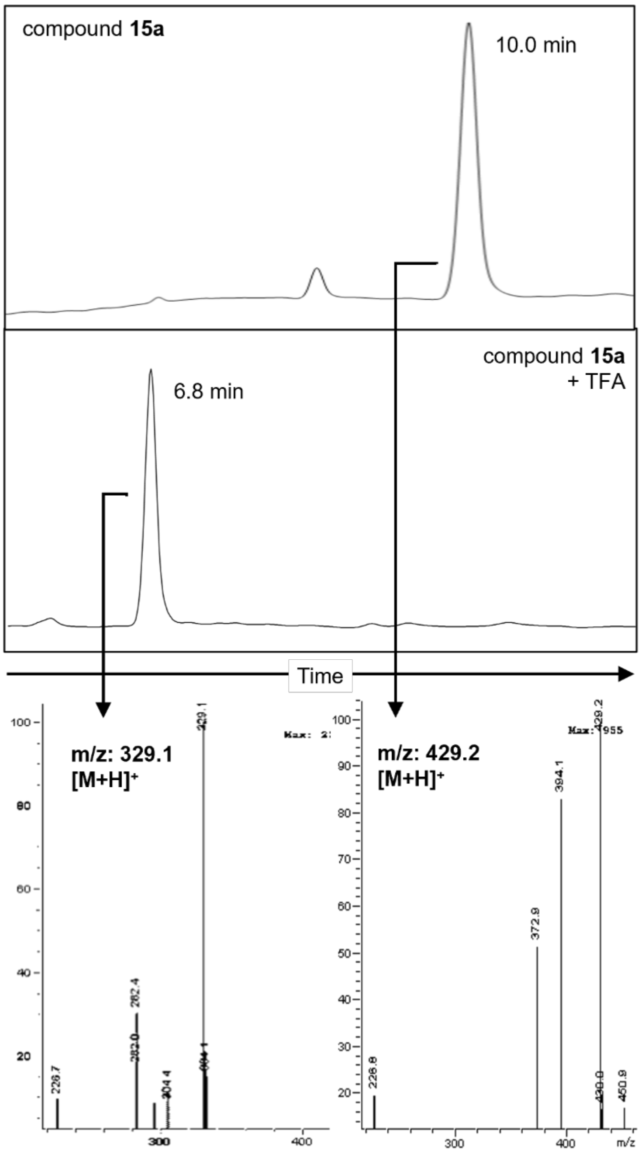

Supplementary Figure 28. HPLC analysis of uncaging of compound 15a. Representative HPLC traces (UV detection: 254 nm) and MS spectra of compound **15a** before and after reaction with TFA:DCM (1:1, 30 min). Calculated m/z values: 429.2 (compound **15a**), 329.1 (compound **15**).

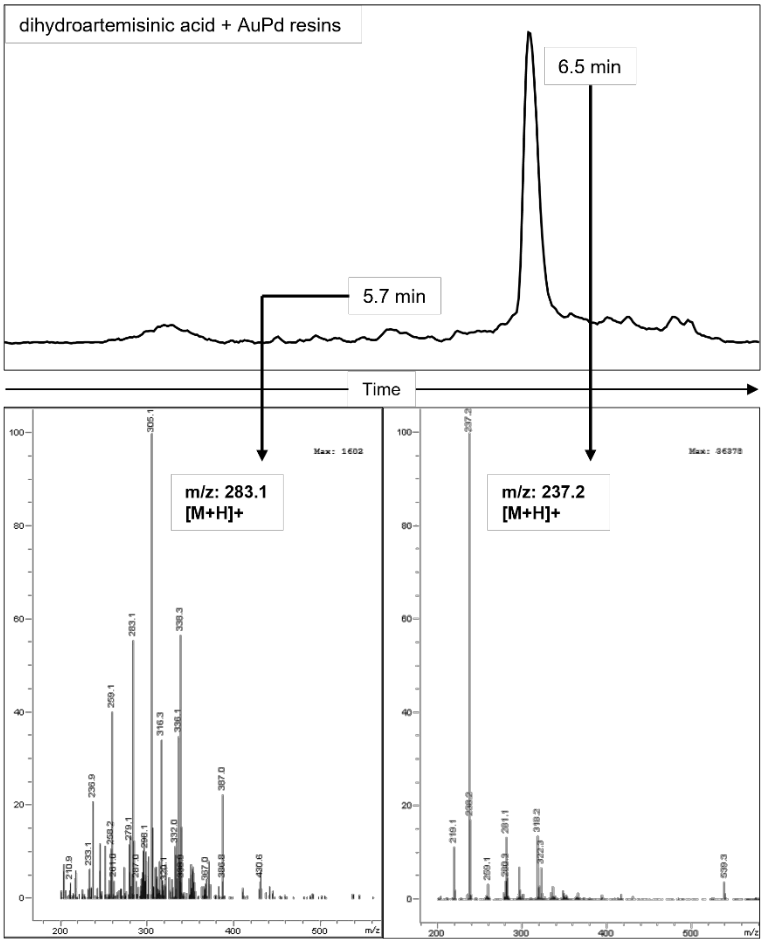

Supplementary Figure 29. HPLC-MS analysis of treatment of dihydroartemisinic acid with AuPd resins in the absence of PS. Representative TIC traces (top panel) and MS spectra (bottom panel) of reaction mixture after dihydroartemisinic acid was illuminated with 640 nm light (0.8 mW cm^{-2}) in the presence of AuPd resins alone (2 mg mL^{-1}) for 16 h. Calculated m/z values: 237.2 (dihydroartemisinic acid), 283.1 (artemisinin).

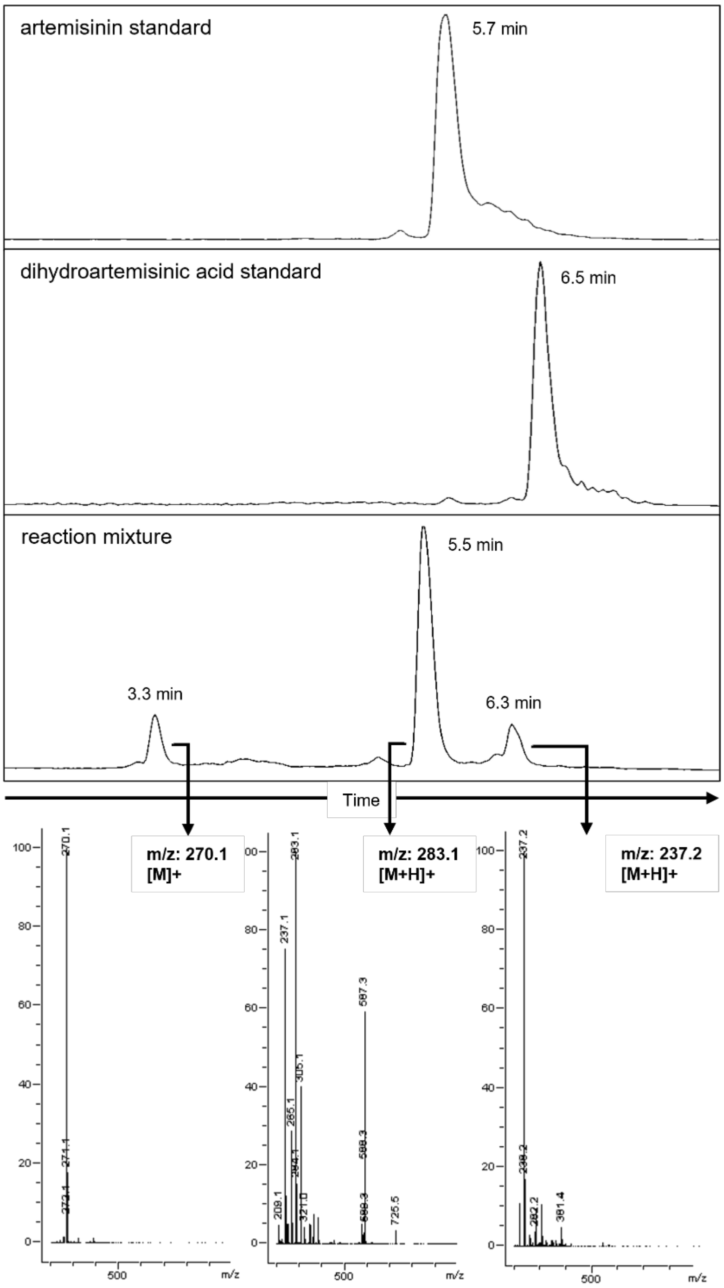

Supplementary Figure 30. HPLC-MS analysis of artemisinin from its precursor dihydroartemisinic acid. Representative TIC traces and MS spectra of pure artemisinin (12 mM, top panel), pure dihydroartemisinic acid (12 mM, middle panel) and reaction mixture after dihydroartemisinic acid was illuminated with 640 nm light (0.8 mW cm^{-2}) in the presence of compound **17b** (1.2 mM) and AuPd resins (2 mg mL^{-1}) (bottom panel) for 10 h. Calculated m/z values: 237.2 (dihydroartemisinic acid), 283.1 (artemisinin), 270.1 (uncaged PS **17**).

Time (h)	compound 17b (caged)	compound 17 (uncaged)	dihydroartemisinic acid	artemisinin
1	78%	22%	>95%	<5%
2	67%	33%	>95%	<5%
4	39%	61%	>95%	<5%
6	17%	83%	71%	29%
8	6%	94%	20%	80%
10	<5%	>95%	18%	82%

Supplementary Figure 31. Time-course HPLC-MS analysis of uncaging of the compound 17b alongside the conversion of dihydroartemisinic acid to artemisinin. Values indicated as percentages of uncaged compound **17** vs caged compound **17b** as well as dihydroartemisinic acid vs artemisinin.

Time (h)	compound 17 (uncaged)	dihydroartemisinic acid	artemisinin
1	10	>95%	<5%
2	20	>95%	<5%
3	30	>95%	<5%
4	40	>95%	<5%
5	50	38%	62%
6	60	36%	64%
7	70	34%	66%
8	80	34%	66%
9	90	31%	69%
10	100	31%	69%

Supplementary Figure 32. Time-course HPLC-MS analysis of conversion of dihydroartemisinic acid to artemisinin by portion-wise addition of compound 17 and continuous light irradiation. Values indicated as percentages of dihydroartemisinic acid vs artemisinin.

3. Supplementary Notes

Supplementary Note 1

We investigated the uncaging of the caged 2-thioxocoumarins by different stimuli. Initial attempts at uncaging compound **14b** using AuPd resins were unsuccessful. Subsequent attempts with other Pd sources (e.g., $Pd(PPh_3)_4$) did not result in any improvement, thus we hypothesized that the complexation of Pd^0 by means of the thiocarbonyl group may inhibit the catalytic activity of the abiotic metal. Similar catalyst poisoning effects have been reported by Della Pina et al. where an exponential decay in catalytic activity was observed in Au nanoparticles after exposure to sulfur-containing biomolecules.[3]

To prove our hypothesis, we synthesized Poc-caged 4-methylaminocoumarin as an analogous structure to compound **14** lacking the sulfur atom in the carbonyl group. The reaction between the Poc-caged 4-methylaminocoumarin and AuPd resins proceeded smoothly to render the free aminocoumarin as the main product, therefore confirming that the thiocarbonyl group was incompatible with Pd-mediated uncaging. Given that the sulfur atom in compound **14** is essential for its photocatalytic activity, we did not analyze the singlet oxygen generation of the resulting 4-methylaminocoumarin.

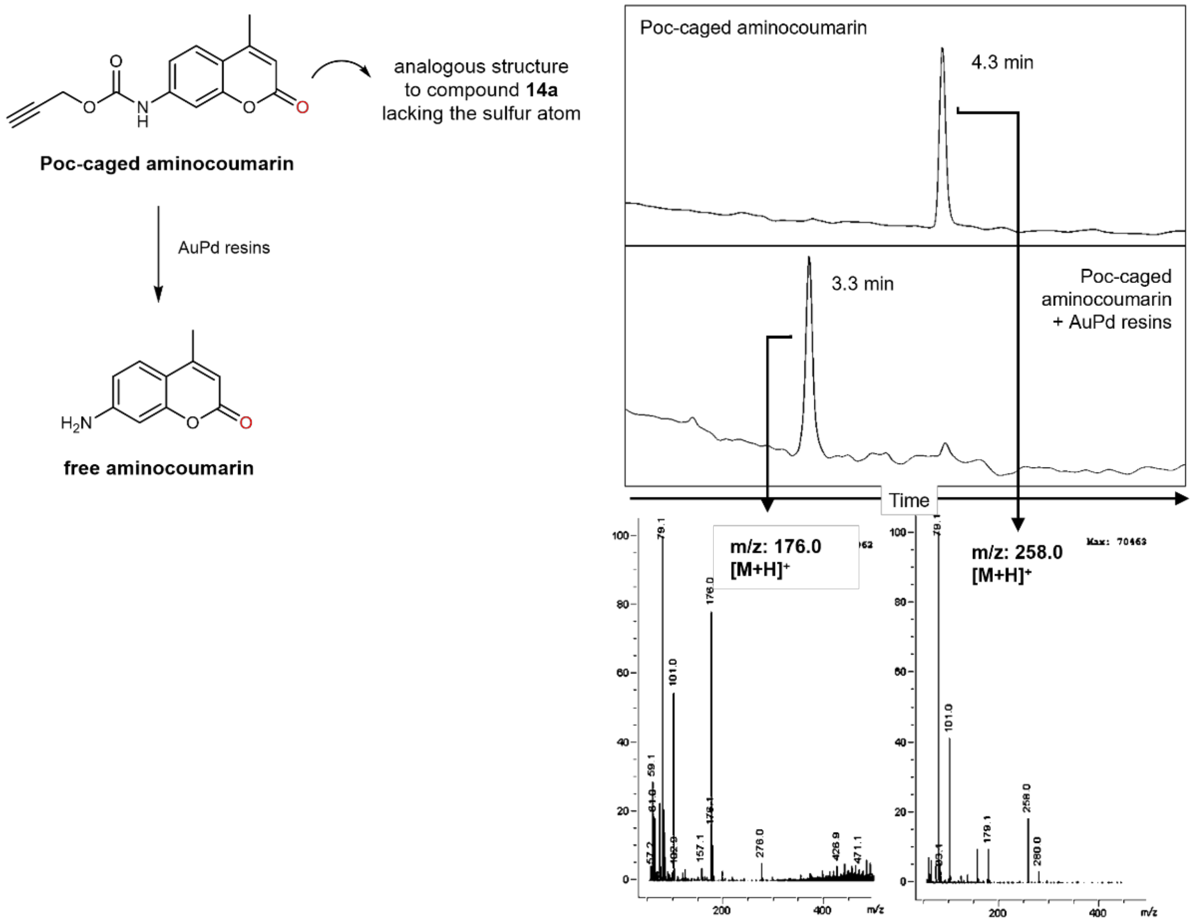

Lastly, we analyzed the uncaging of the Boc-caged 2-thioxocoumarin **14a** in acidic media. The reaction between compound **14a** and TFA led to the successful release of compound **14**, yet oxygenation also proceeded to some extent to render the non-photosensitive aminocoumarin (t_R=4.7 min). Altogether, these observations suggest that 1) PS containing thiocarbonyl groups might be incompatible with Pd-mediated uncaging, and 2) effective caged 2-thioxocoumarins might require the use of protecting groups that are cleaved without affecting the integrity of the thiocarbonyl group.

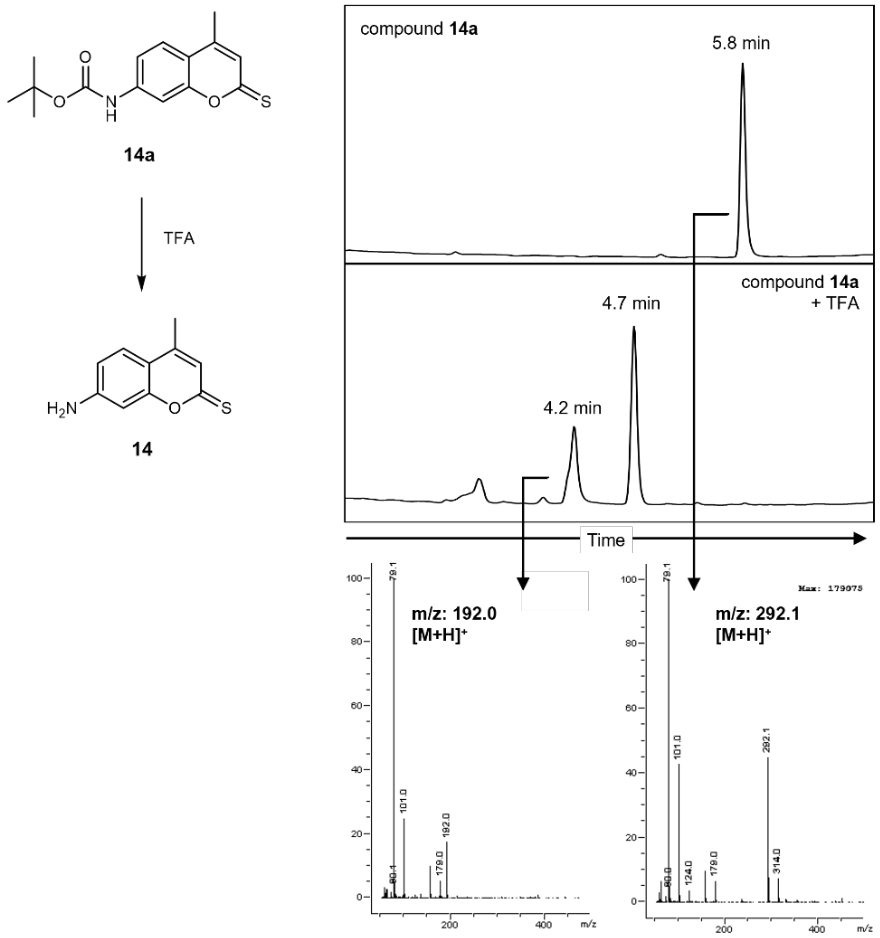
TFA
compound 14a
5.8 min
compound 14a
+ TFA
4.7 min
4.2 min
Time
m/z: 192.0
[M+H]+
m/z: 292.1
[M+H]+

Supplementary Note 2

In order to optimize the singlet oxygen-mediated conversion of dihydroartemisinic acid to artemisinin, we prepared solutions of dihydroartemisinic acid (1 mM) in the indicated solvent systems including 4% (v/v) TFA in glass vials equipped with stirring bars. Next, a solution of the different PS (i.e., compounds **17** or **17b**) in the same solvent system were added, followed by the decaging agent as indicated below. The reaction mixtures were stirred overnight under irradiation at 640 nm (0.7-0.8 mW), then analyzed by HPLC-MS.

First, we attempted the conversion of dihydroartemisinic acid into artemisinin using the uncaged PS 17 as the photocatalyst (entries 1-6). Irrespective of the polarity of the solvent system, which ranged from MeOH to MeOH: toluene (1:1) (Note: higher amounts of toluene resulted in poor solubility) and the amount of PS -from 1% (mol/mol) to stoichiometric- only traces of artemisinin were detected. Next, we investigated the caged PS 17b. As expected, compound **17b** alone in the absence of trigger (entries 7 and 8) did not improve the conversion, consistently with the inability of compound **17b** to generate singlet oxygen (data shown in Figure 5c). Subsequently, we screened different conditions to uncage the PS for controlled release of singlet oxygen using $Pd(PPh_3)_4$ as the trigger (entries 9-16). In this case, the solvent system proved crucial, and artemisinin was formed with limited conversion (20%) only when DCM was employed (entry 14). This is likely due to the very good solubility of $Pd(Ph_3)_4$ in this solvent. Following the positive outcome by metal-catalyzed uncaging of the compound **17b**, next we combined the use of compound 17b with AuPd resins (2 mg mL^{-1}) in MeOH:H_2O (3:7). Under these conditions, we observed 50% conversion of dihydroartemisinic acid into artemisinin (entry 15). Finally, we slightly scaled up the reaction (from 1 mM to 12 mM dihydroartemisinic acid) while keeping the same experimental conditions and observed 80% conversion by HPLC-MS (entry 16), confirming the suitability of compound **17b** and AuPd resins for controlled singlet oxygen generation in the synthesis of artemisinin. Of note, only traces of artemisinin were observed when a solution of dihydroartemisinic acid was irradiated for 16 h only in the presence of AuPd resins (Supplementary Figure 29).

entry	PS	amount PS	trigger	amount trigger	solvent	% conversion to artemisinin
1	**17**	1 eq	-	N/A	MeOH	no reaction
2	**17**	10%	-	N/A	MeOH	traces (<5%)
3	**17**	1%	-	N/A	MeOH	no reaction
4	**17**	1 eq	-	N/A	MeOH:toluene (1:1)	no reaction
5	**17**	10%	-	N/A	MeOH:toluene (1:1)	traces (<5%)
6	**17**	1%	-	N/A	MeOH:toluene (1:1)	no reaction
7	**17b**	10%	-	N/A	MeOH	no reaction
8	**17b**	10%	-	N/A	MeOH:toluene (1:1)	no reaction
9	**17b**	10%	$Pd(PPh_3)_4$	10%	MeOH	no reaction
10	**17b**	10%	$Pd(PPh_3)_4$	10%	MeOH:toluene (1:1)	no reaction
11	**17b**	10%	$Pd(PPh_3)_4$	10%	MeCN	no reaction
12	**17b**	10%	$Pd(PPh_3)_4$	10%	THF	no reaction
13	**17b**	10%	$Pd(PPh_3)_4$	10%	Dioxane	no reaction
14	**17b**	10%	$Pd(PPh_3)_4$	10%	DCM	20%
15	**17b**	10%	AuPd resins	2 mg mL^{-1}	H_2O:MeOH (7:3)	50%
16	**17b**	10%	AuPd resins	2 mg mL^{-1}	H_2O:MeOH (7:3)	80%

NMR Spectra

Supplementary Figure 33. 1H NMR (top) and 13C NMR (bottom) spectra of compound **1** (DMSO-d6)

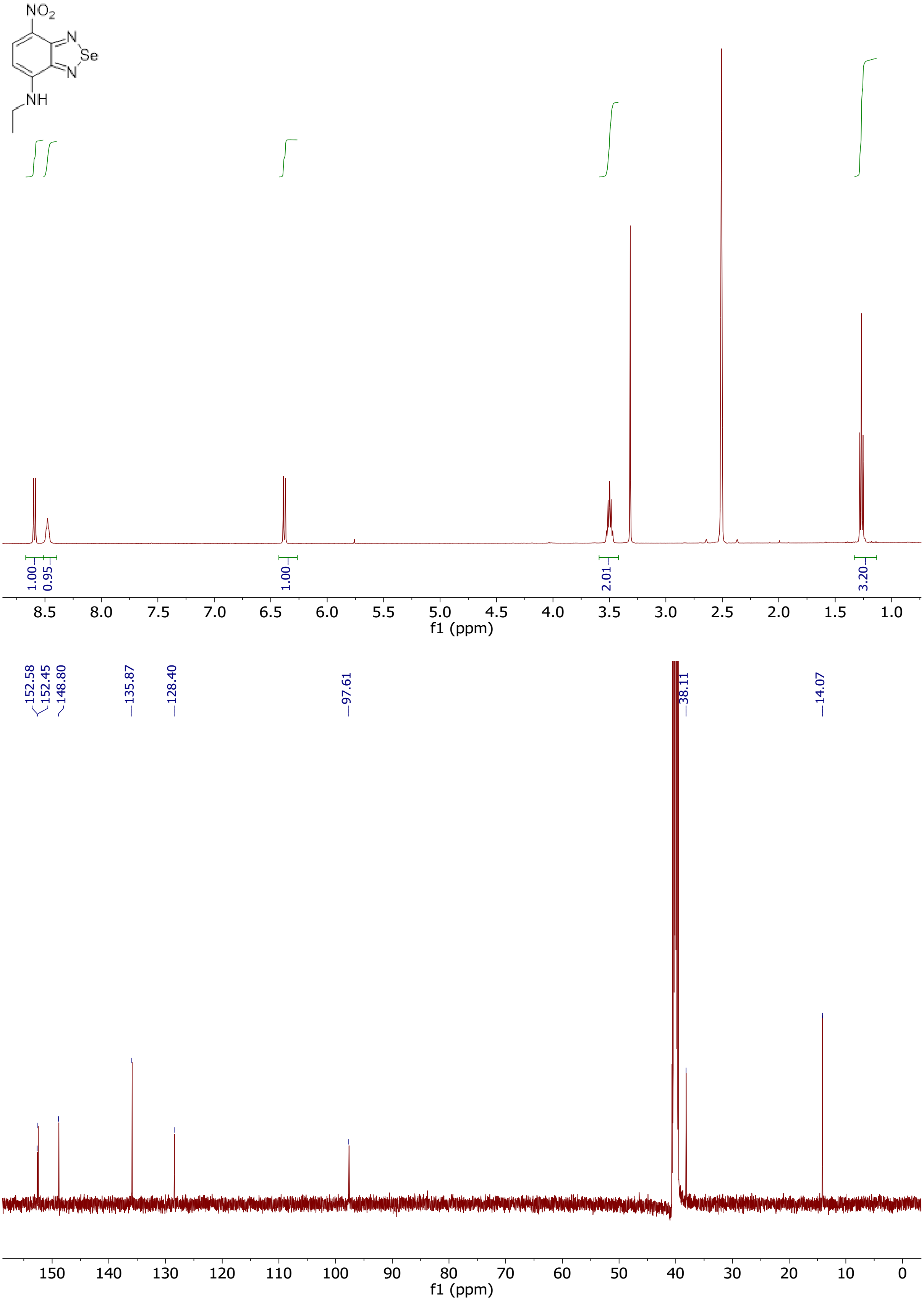

Supplementary Figure 34. 1H NMR (top) and 13C NMR (bottom) spectra of compound **2** (DMSO-d6)

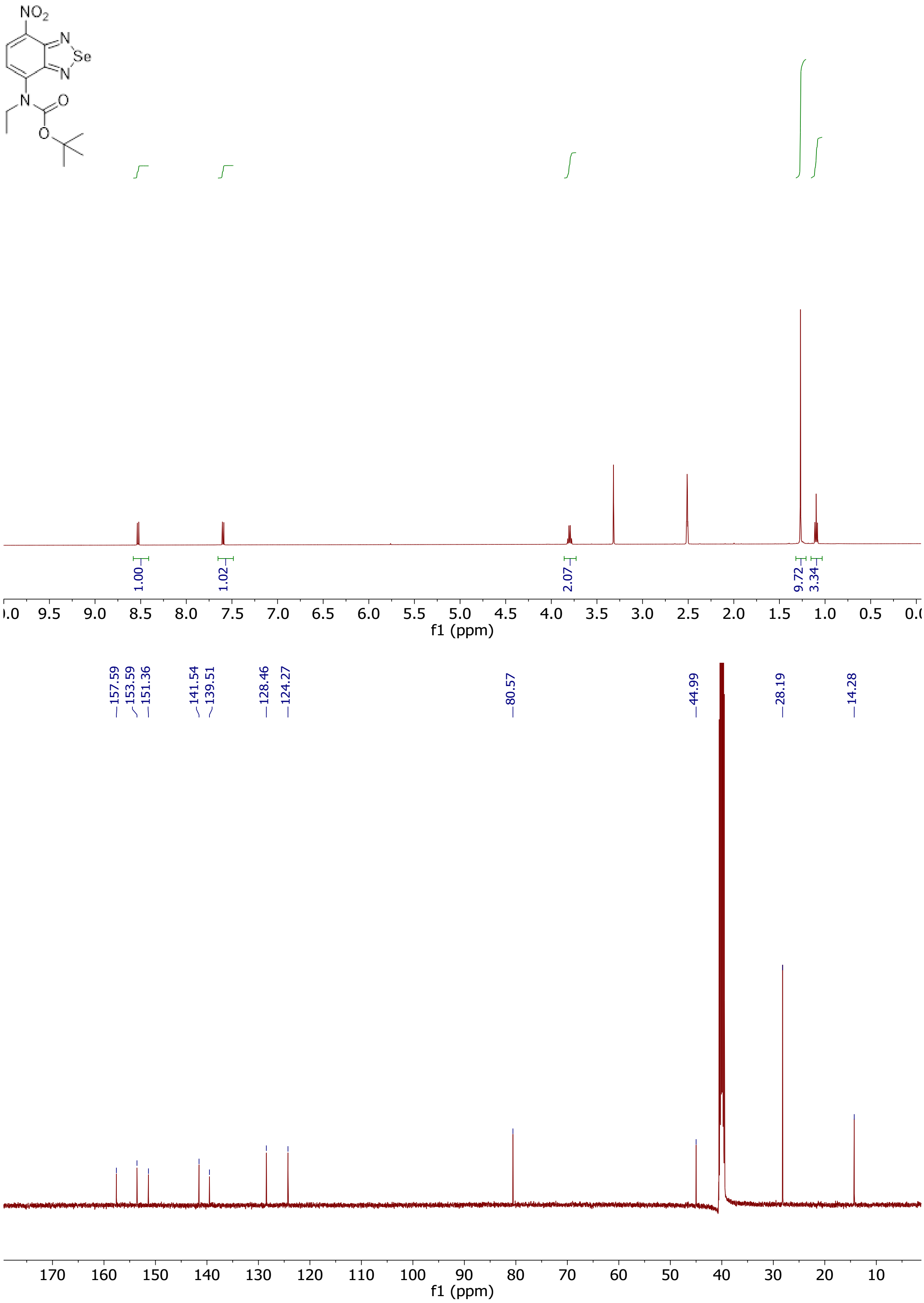

Supplementary Figure 35. 1H NMR (top) and 13C NMR (bottom) spectra of compound **3** (DMSO-d6)

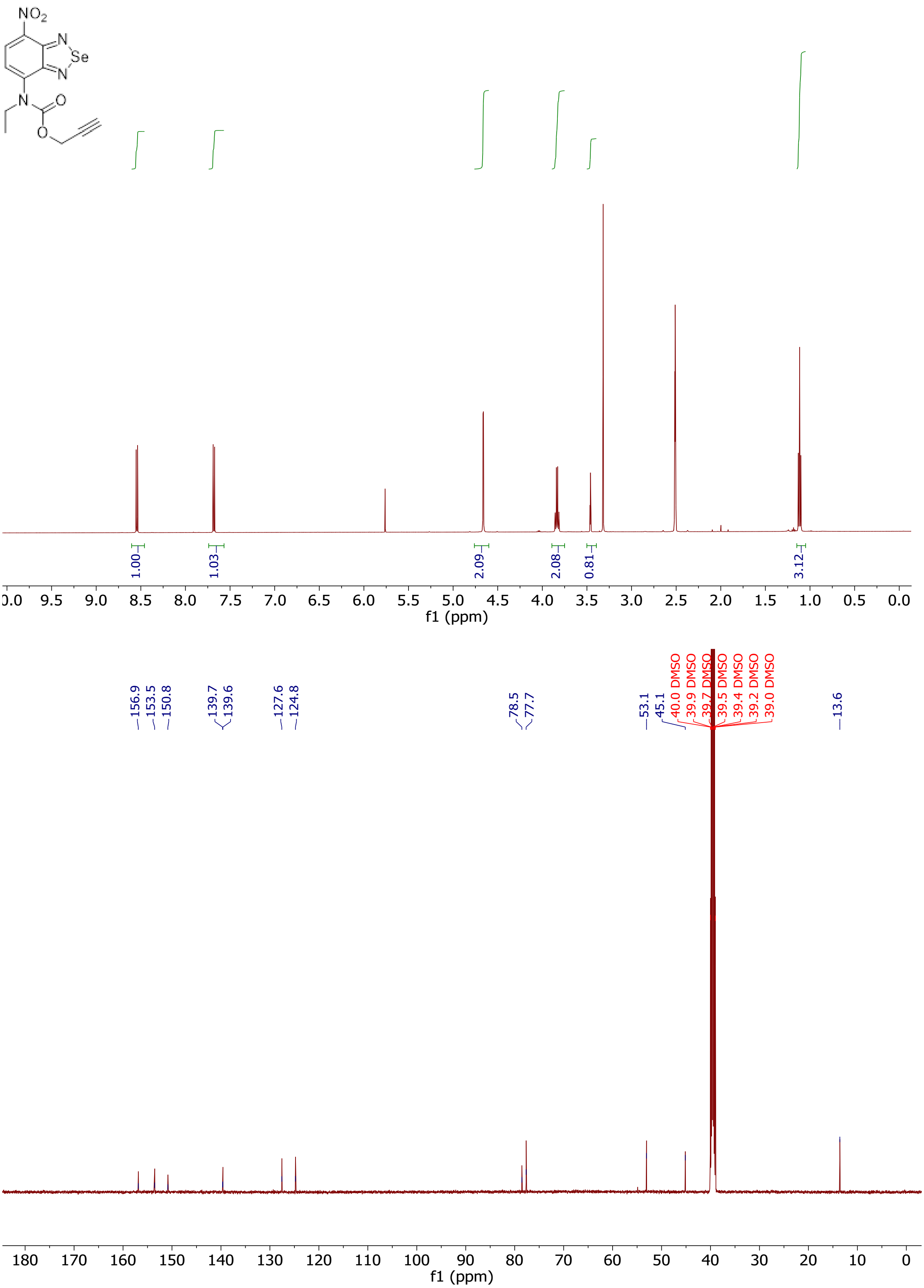

Supplementary Figure 36. 1H NMR (top) and 13C NMR (bottom) spectra of compound **4** ($CDCl_3$)

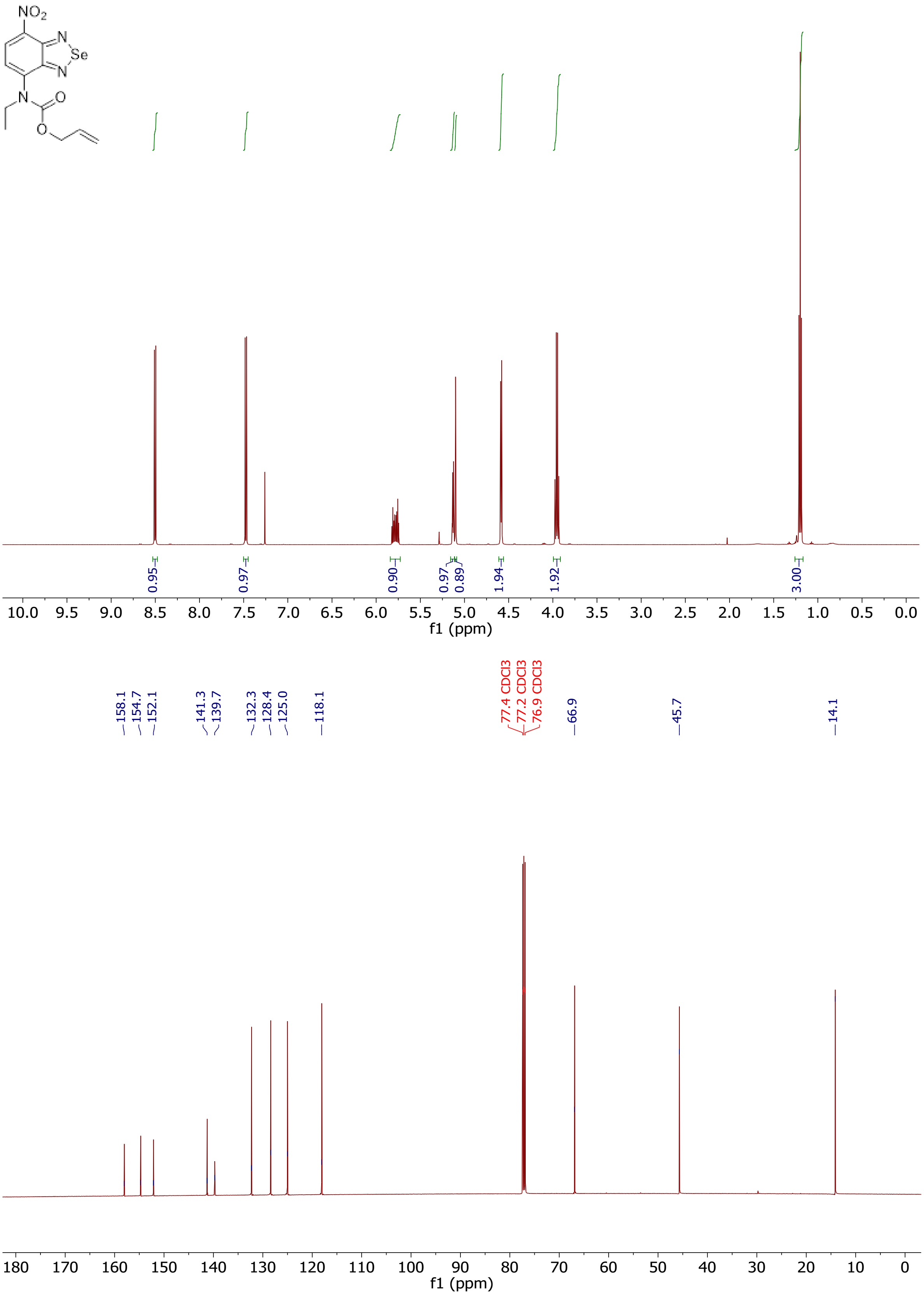

Supplementary Figure 37. 1H NMR (top) and 13C NMR (bottom) spectra of compound **5** ($CDCl_3$)

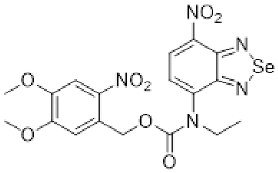

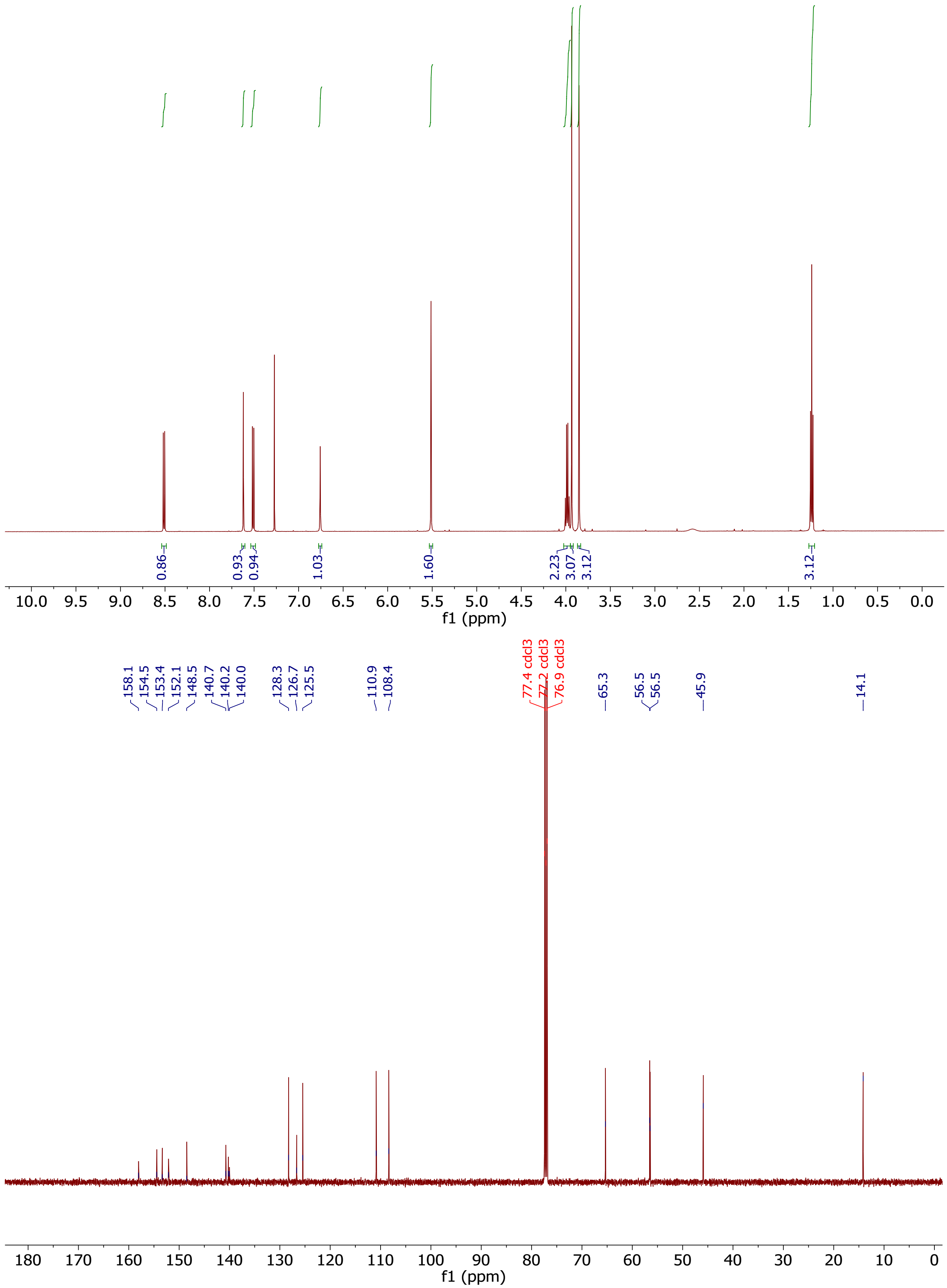

Supplementary Figure 38. 1H NMR (top) and 13C NMR (bottom) spectra of compound **6** (MeOD)

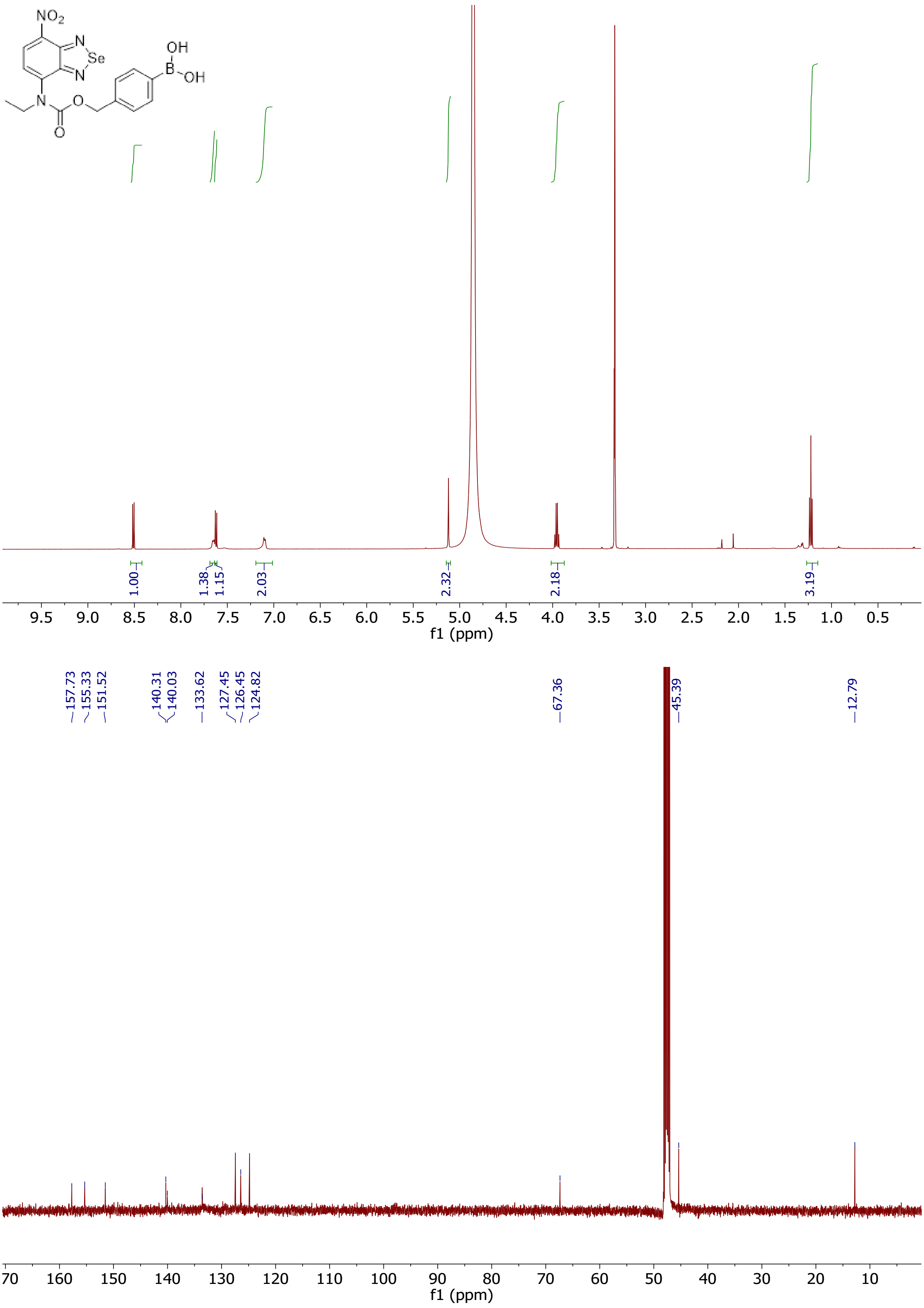

Supplementary Figure 39. 1H NMR (top) and 13C NMR (bottom) spectra of compound **7** (DMSO-d$_6$)

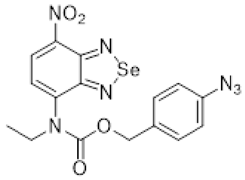

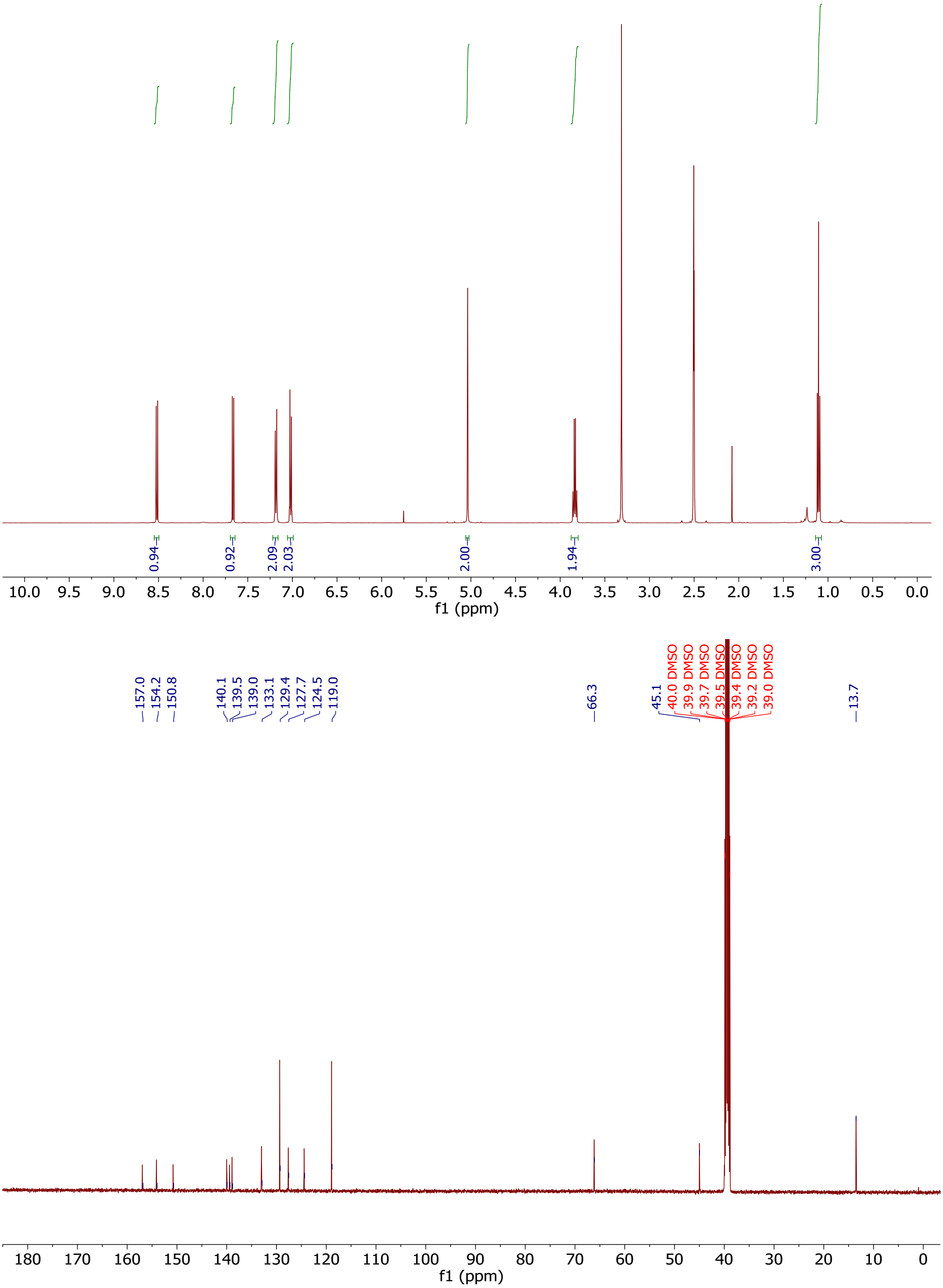

Supplementary Figure 40. 1H NMR (top) and 13C NMR (bottom) spectra of compound **8** ($CDCl_3$)

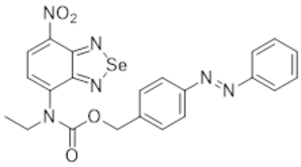

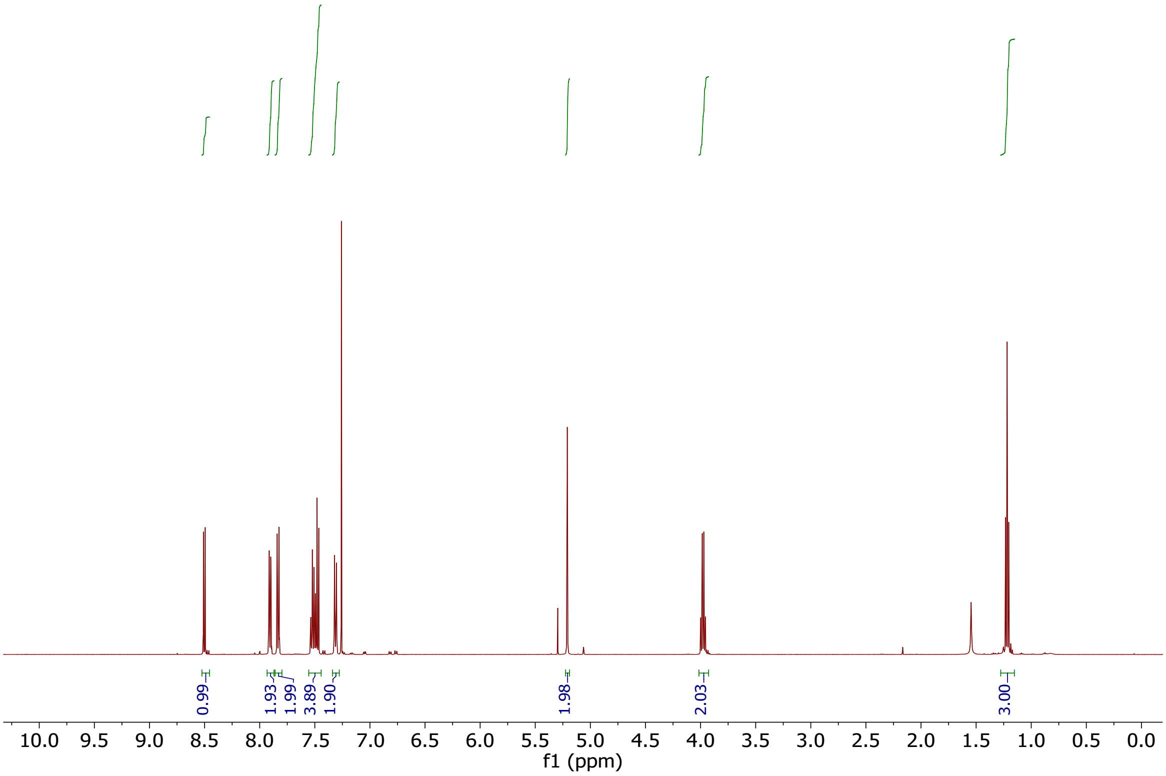

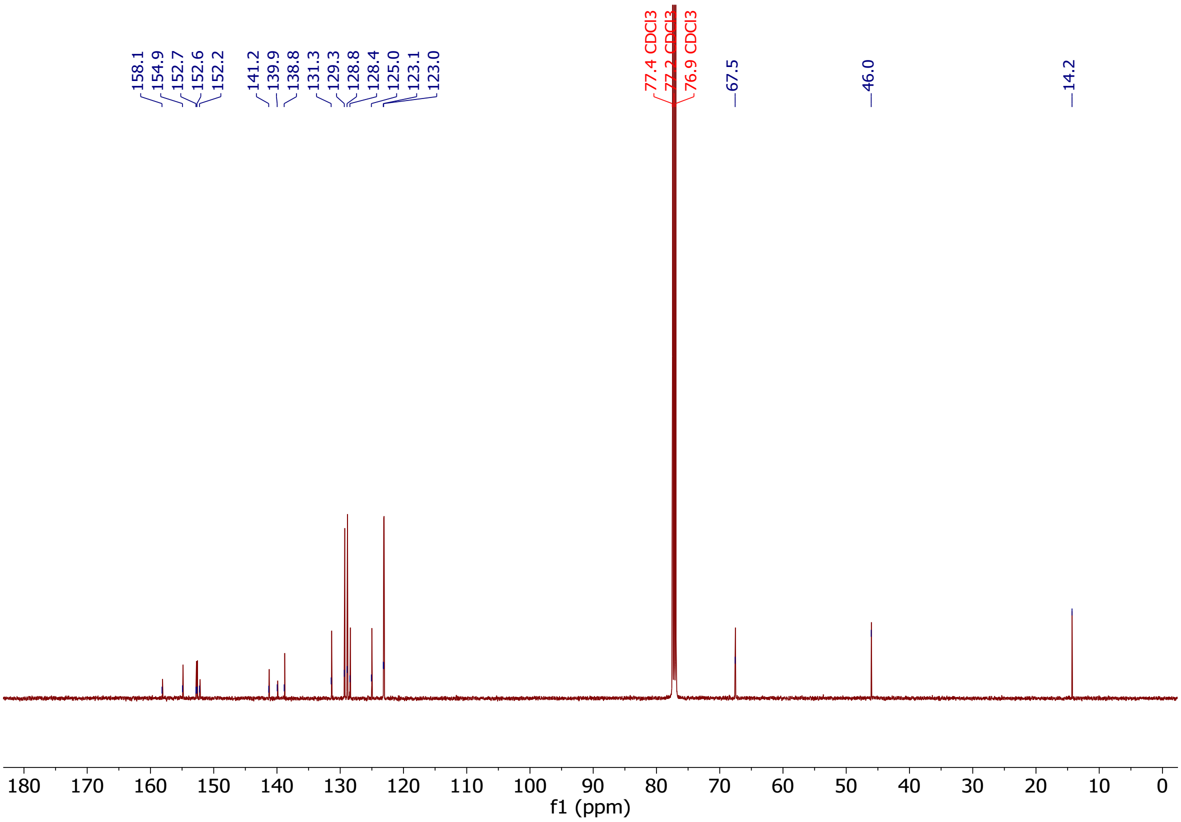

Supplementary Figure 41. 1H NMR (top) and 13C NMR (bottom) spectra of compound **9** ($CDCl_3$)

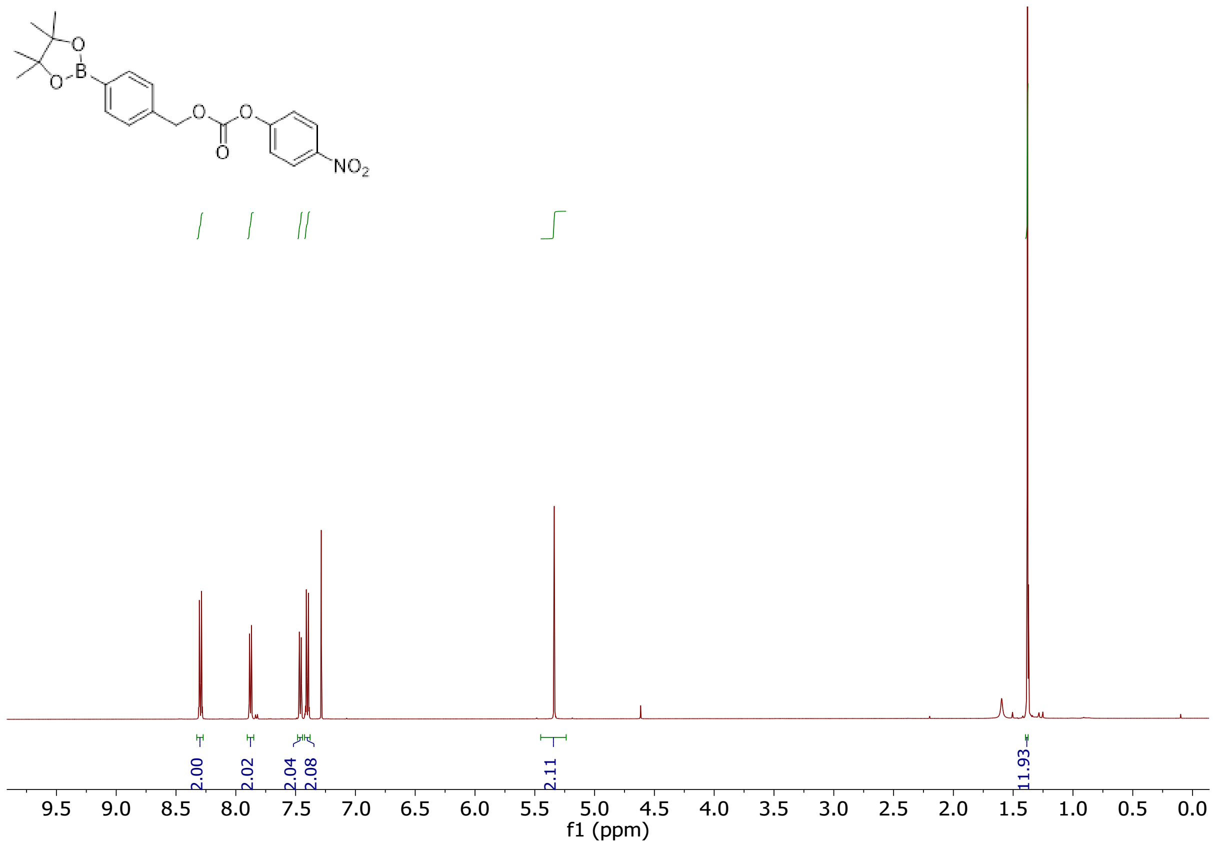
O
B
O
O
O
O
NO2
2.00
2.02
2.04
2.08
2.11
11.93
9.5 9.0 8.5 8.0 7.5 7.0 6.5 6.0 5.5 5.0 4.5 4.0 3.5 3.0 2.5 2.0 1.5 1.0 0.5 0.0
f1 (ppm)

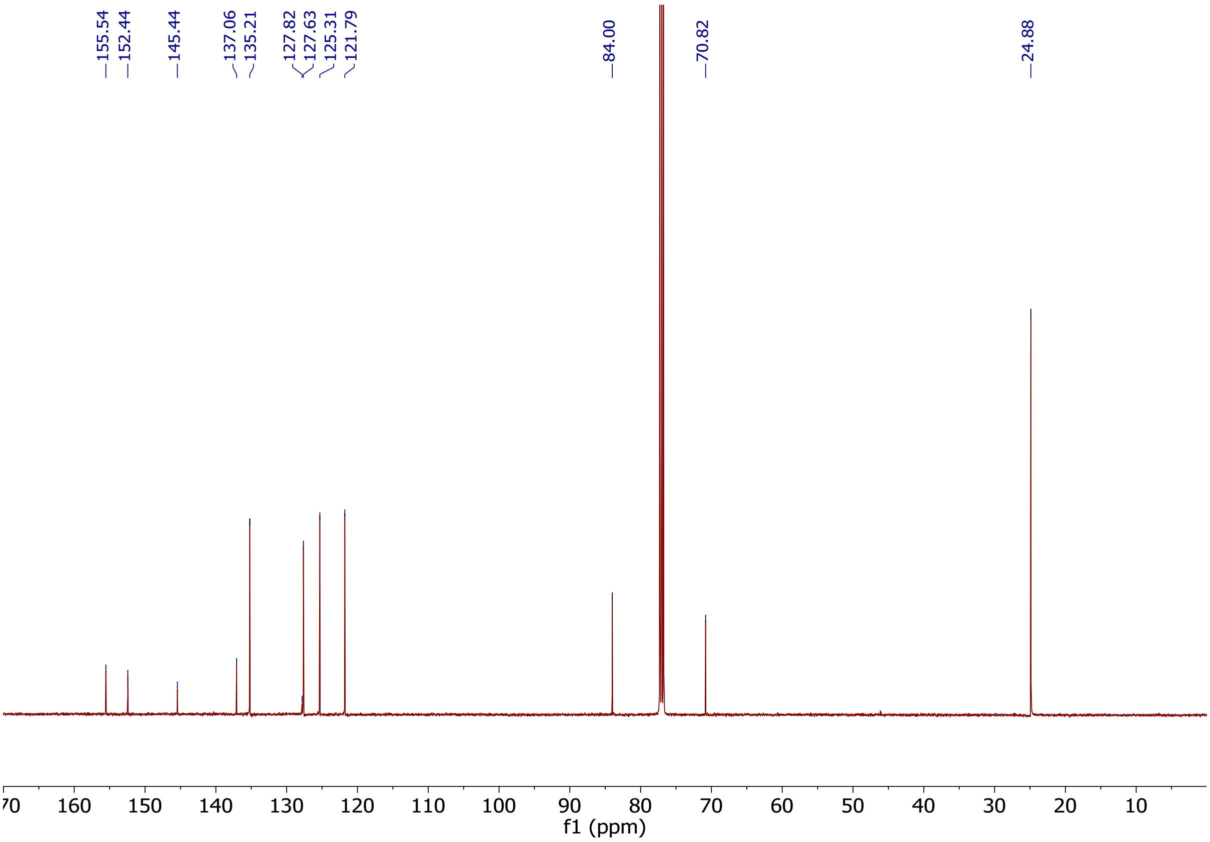
155.54
152.44
145.44
137.06
135.21
127.82
127.63
125.31
121.79
84.00
70.82
24.88
160 150 140 130 120 110 100 90 80 70 60 50 40 30 20 10
f1 (ppm)

Supplementary Figure 42. 1H NMR (top) and 13C NMR (bottom) spectra of precursor compound **10** (DMSO-d6)

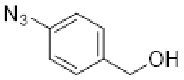

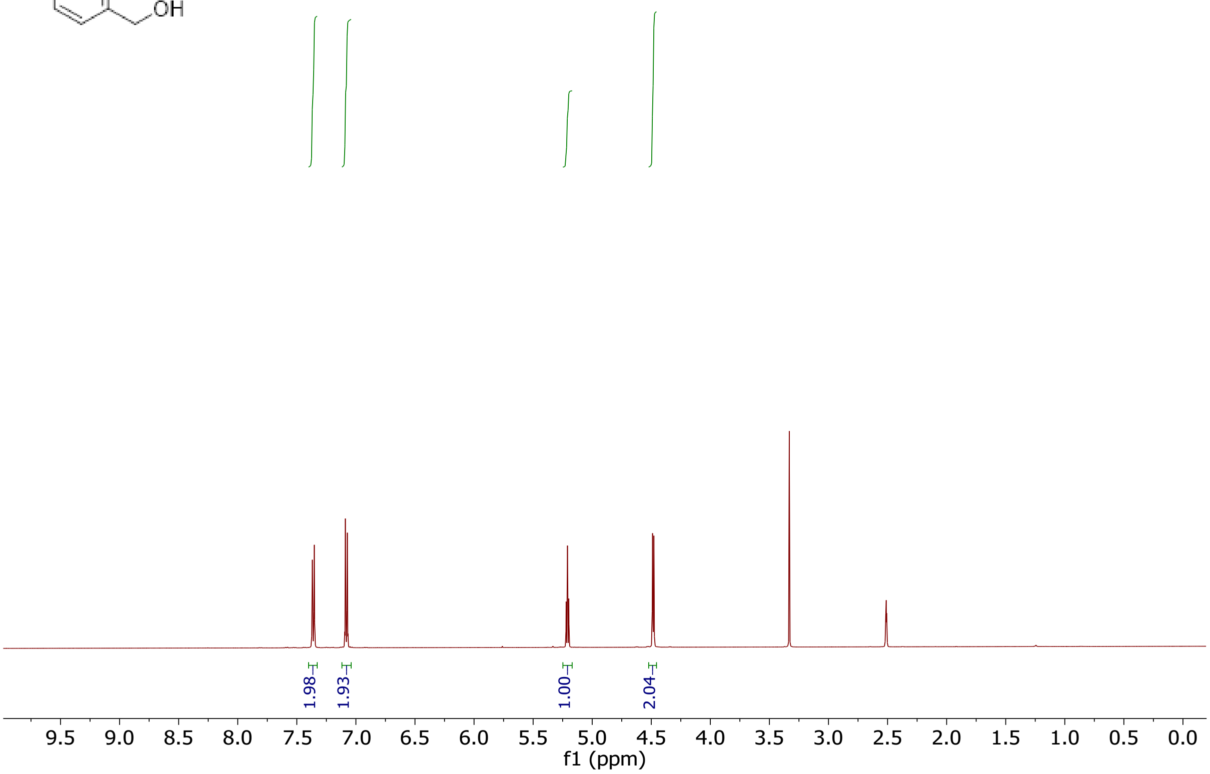

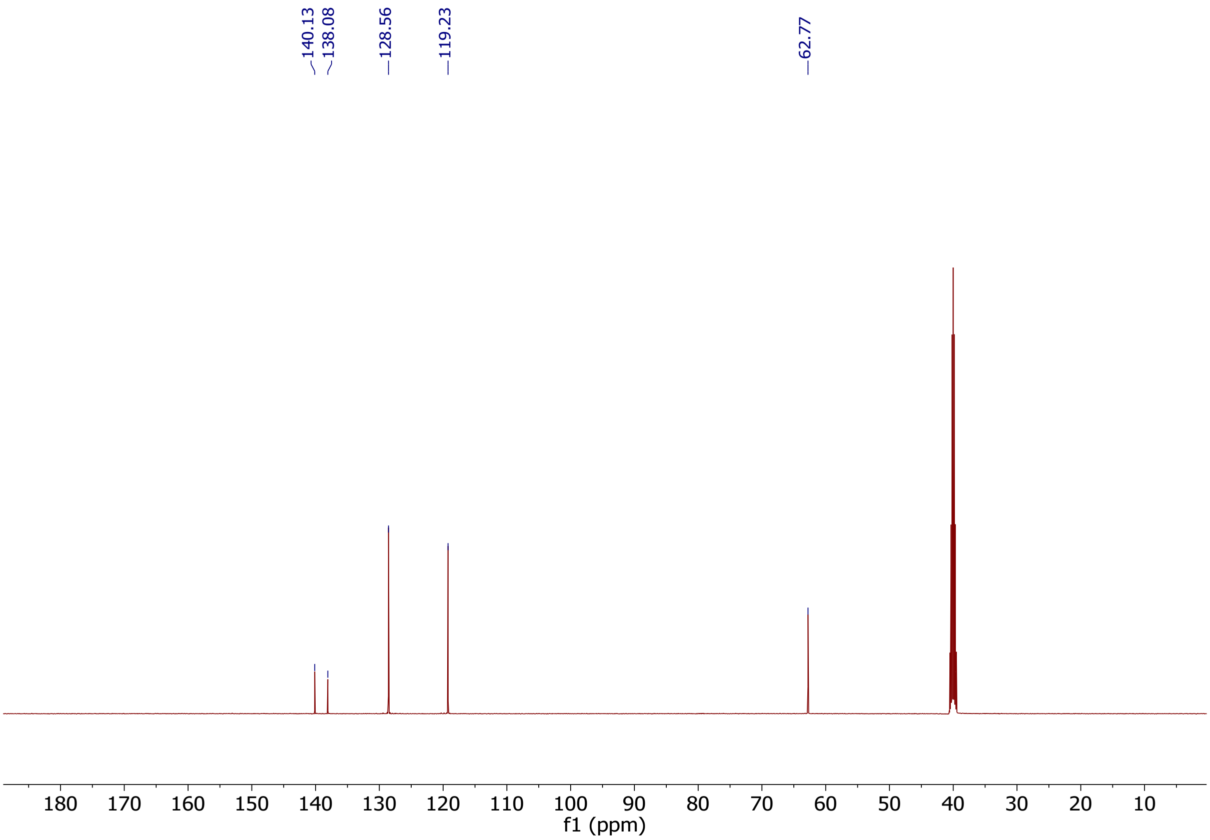

Supplementary Figure 43. 1H NMR (top) and 13C NMR (bottom) spectra of compound **10** ($CDCl_3$)

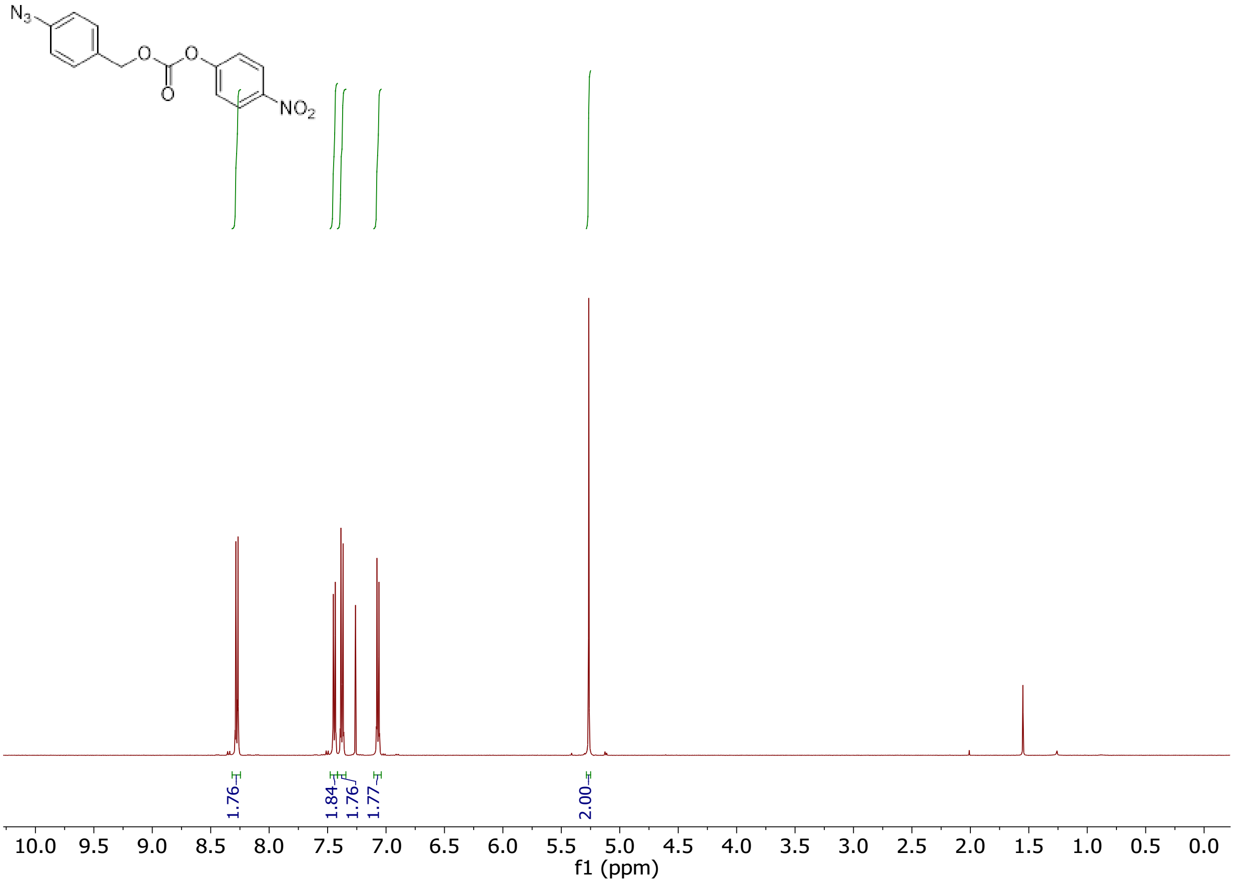

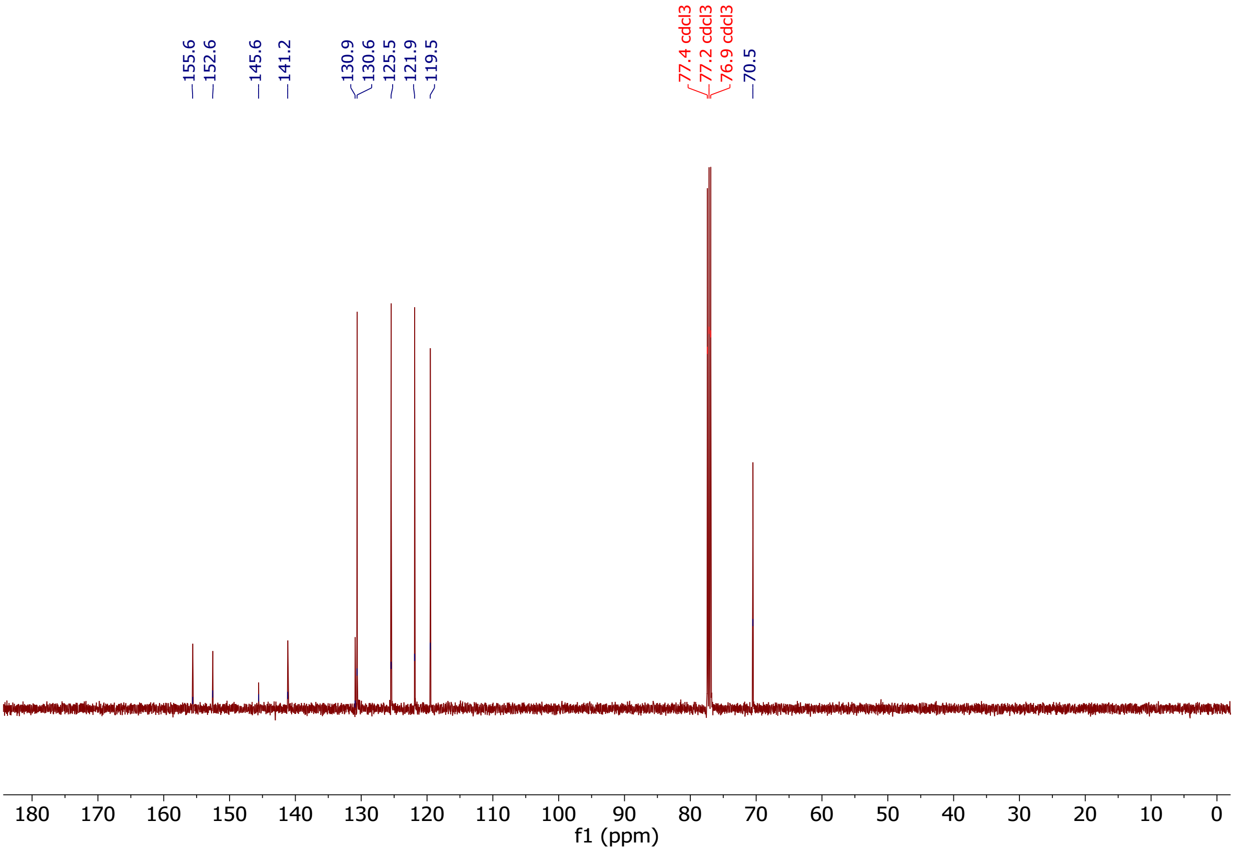

Supplementary Figure 44. 1H NMR (top) and 13C NMR (bottom) spectra of precursor compound **11** (DMSO-d6)

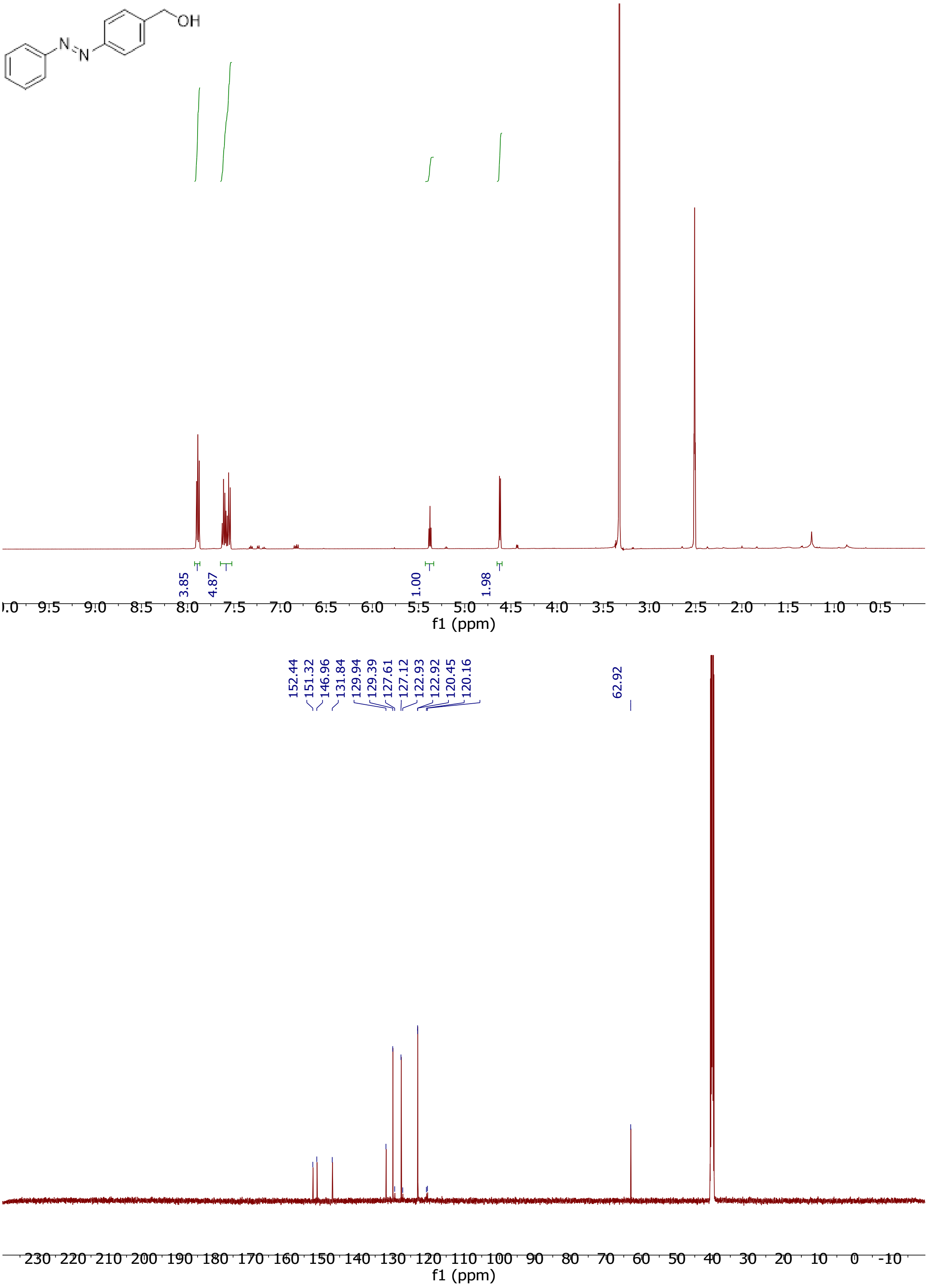

Supplementary Figure 45. 1H NMR (top) and 13C NMR (bottom) spectra of compound **11** ($CDCl_3$)

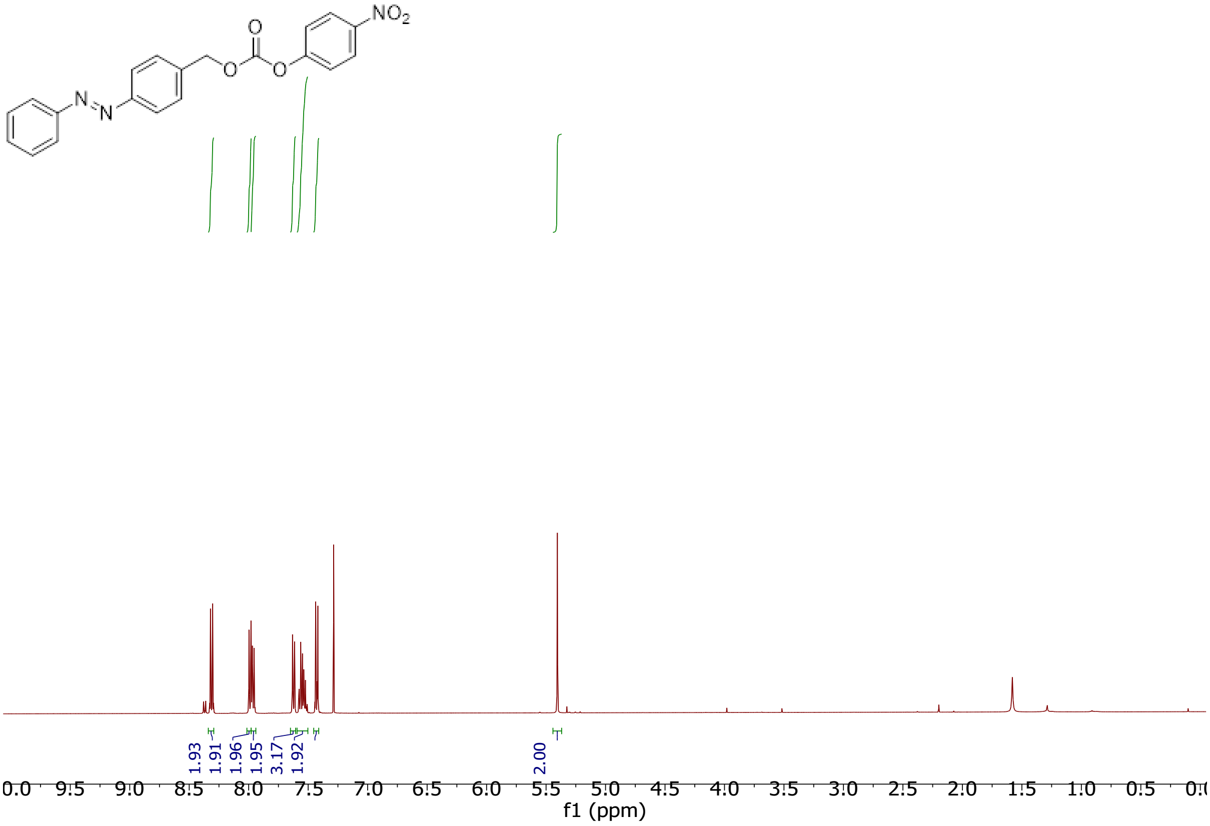

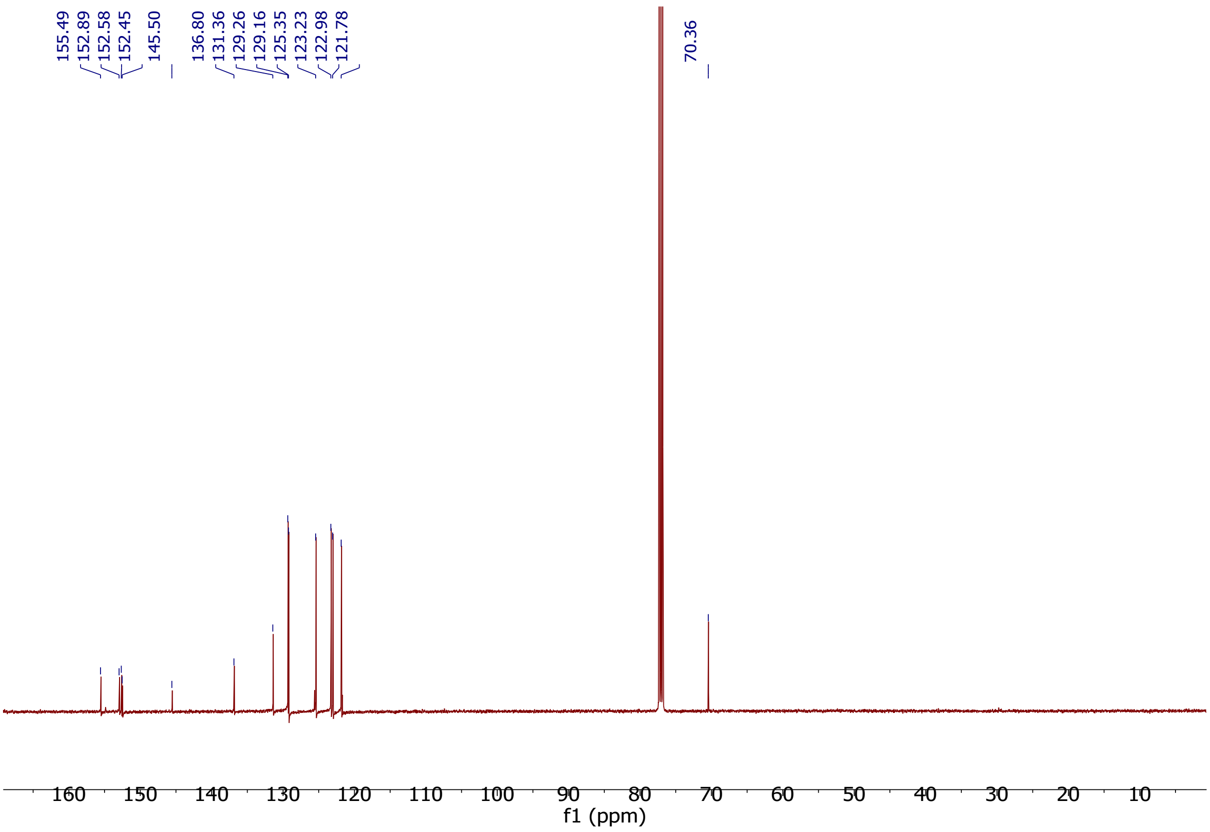

Supplementary Figure 46. 1H NMR (top) and 13C NMR (bottom) spectra of compound **12** (DMSO-d_6)

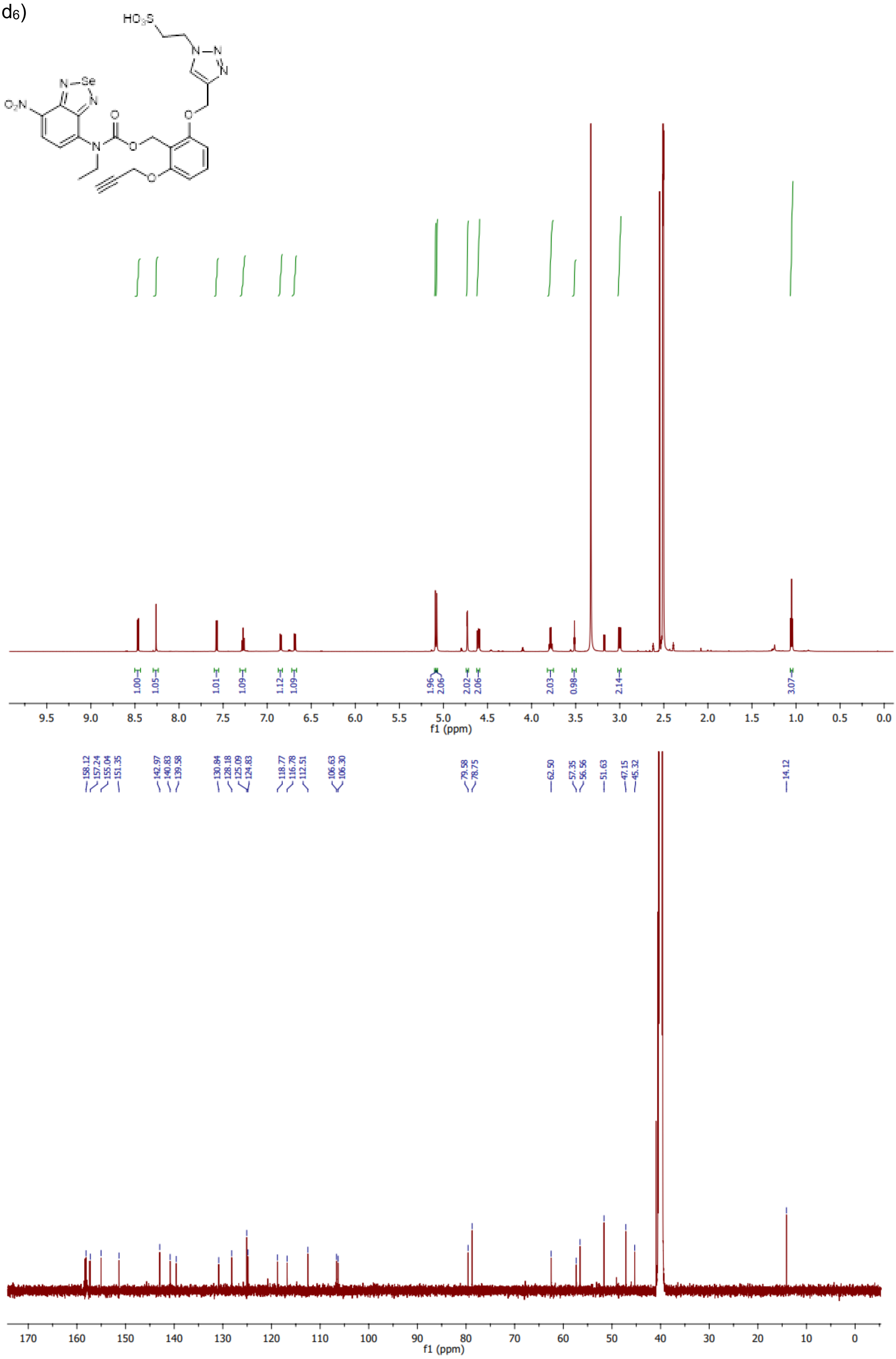

Supplementary Figure 47. 1H NMR (top) and 13C NMR (bottom) spectra of compound **13** ($CDCl_3$)

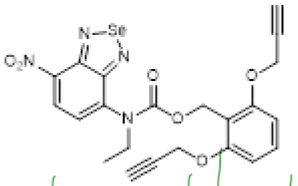

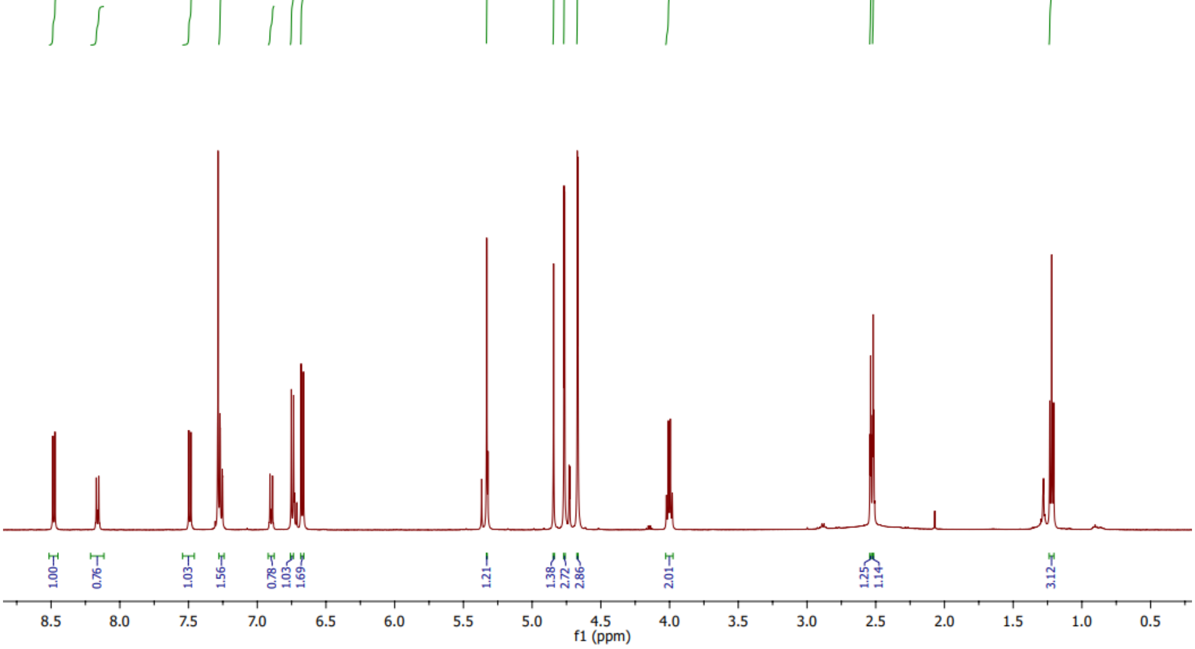

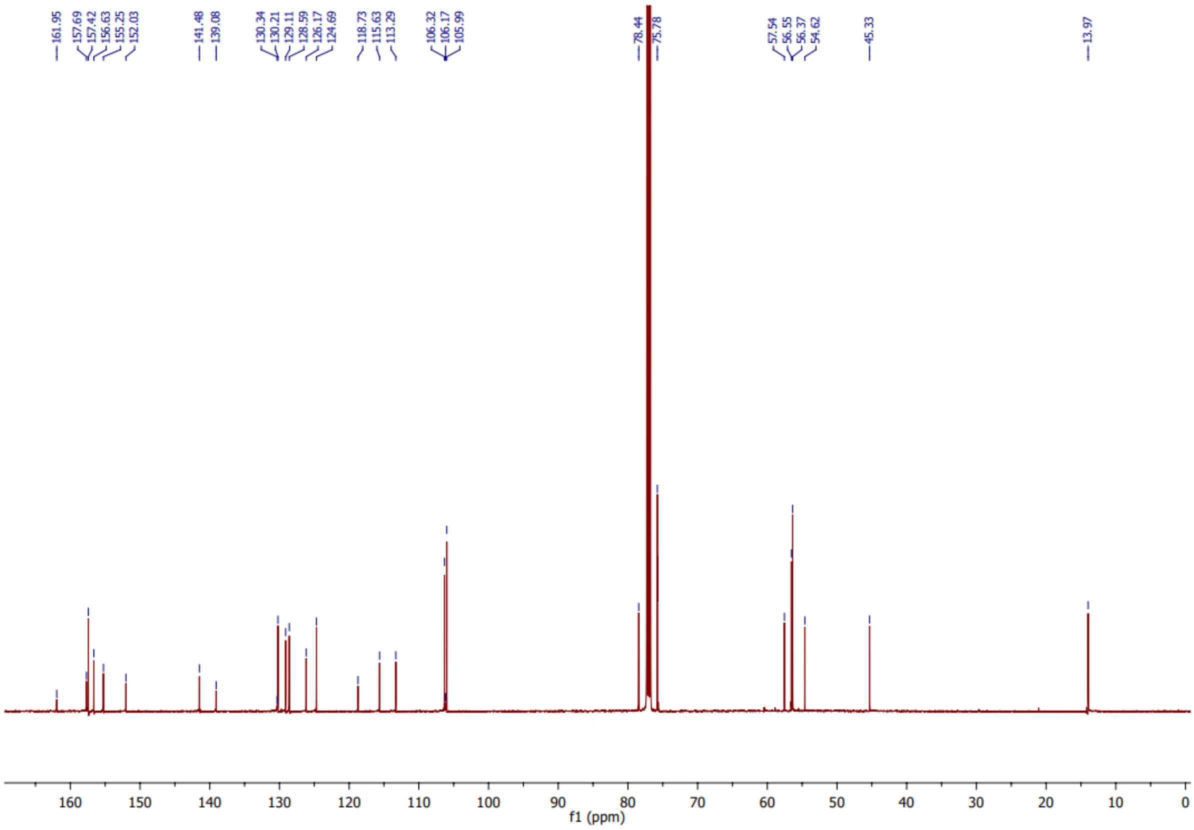

Supplementary Figure 48. 1H NMR (top) and 13C NMR (bottom) spectra of precursor compound **14a** ($CDCl_3$)

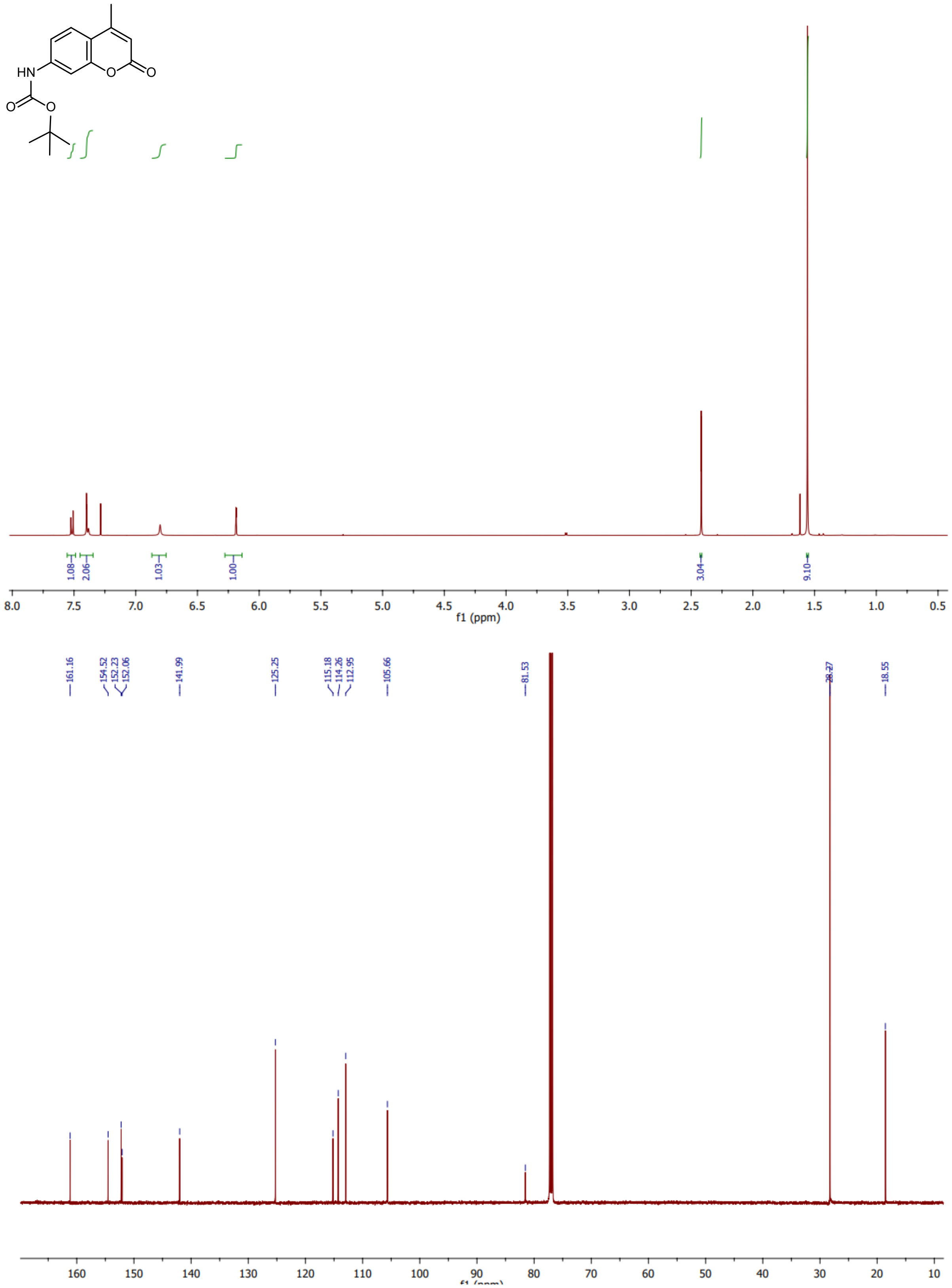

Supplementary Figure 49. 1H NMR (top) and 13C NMR (bottom) spectra of compound **14a** ($CDCl_3$)

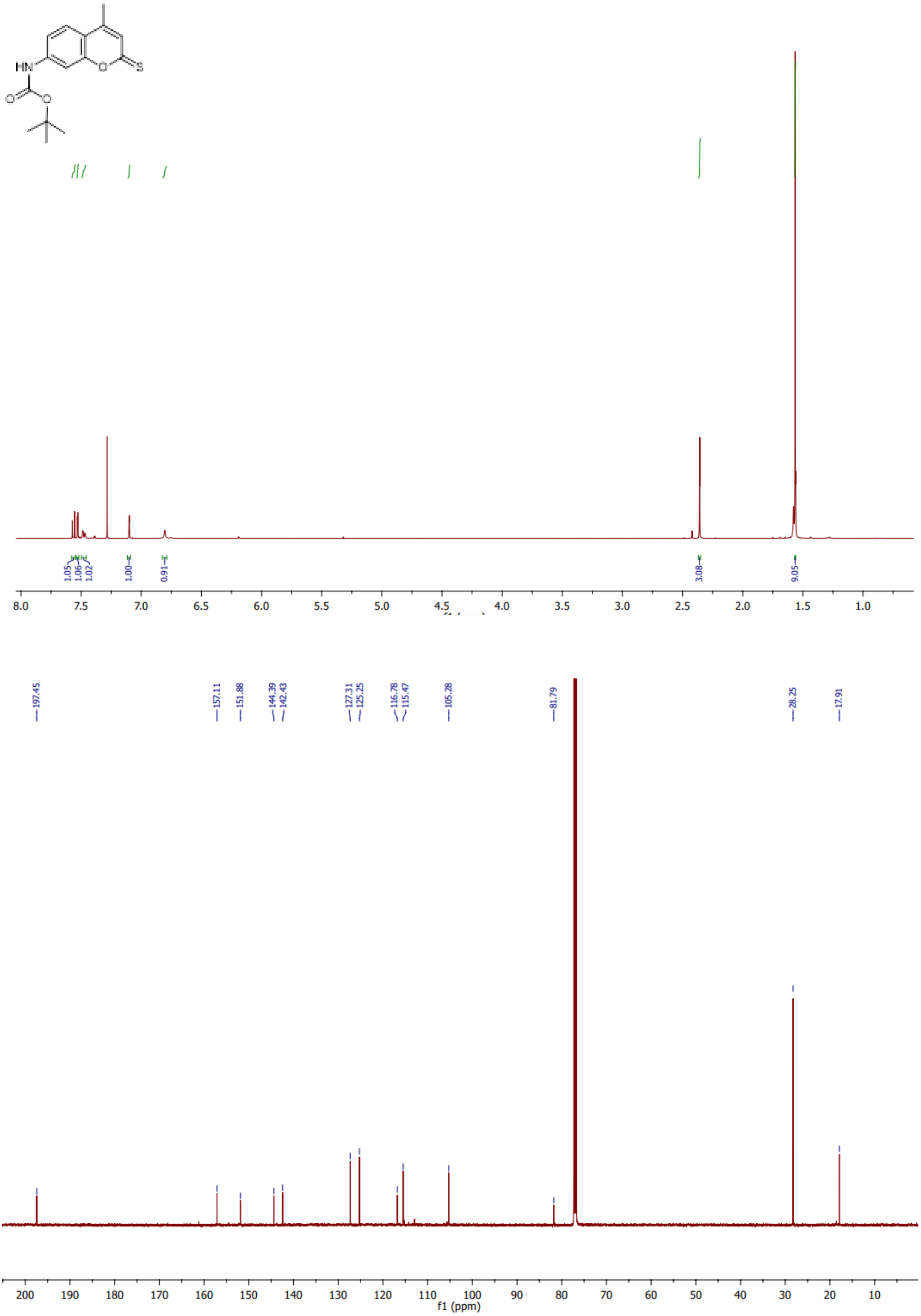

Supplementary Figure 50. 1H NMR (top) and 13C NMR (bottom) spectra of precursor compound **14b** (DMSO-d_6)

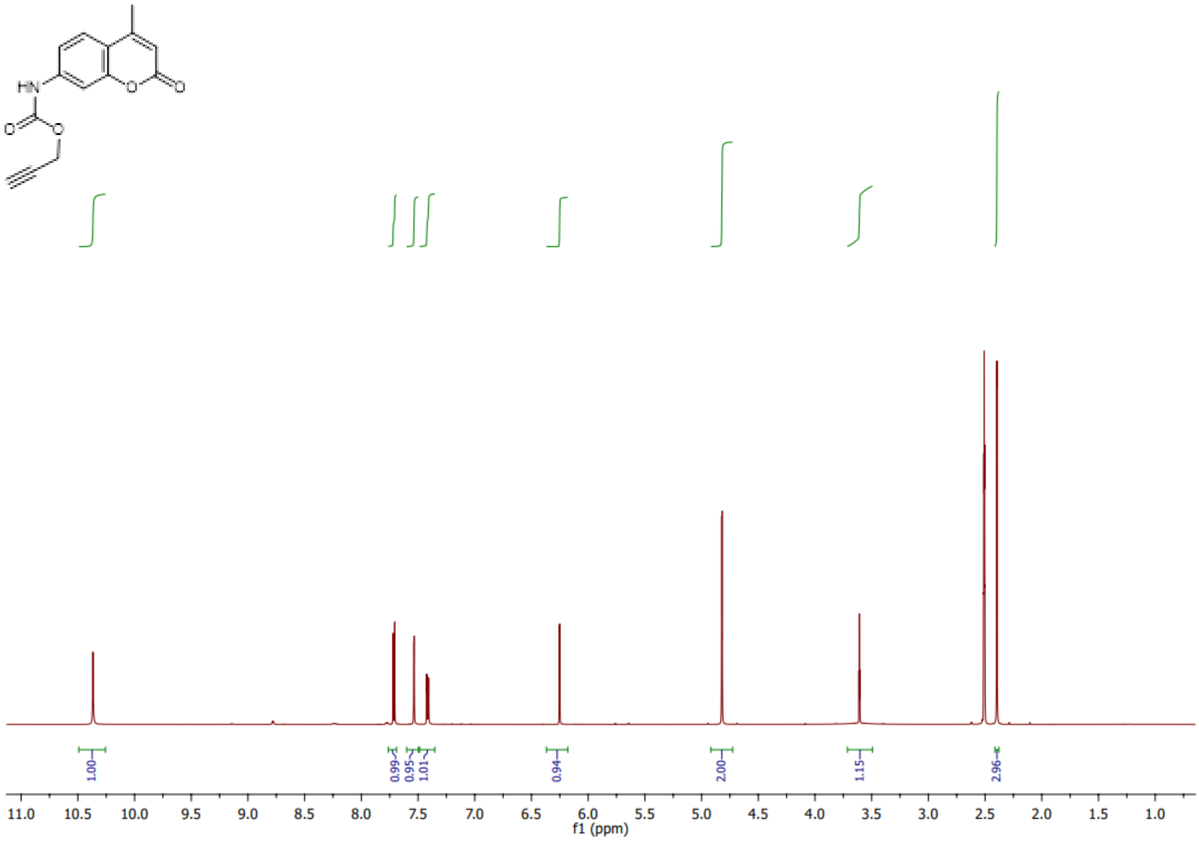

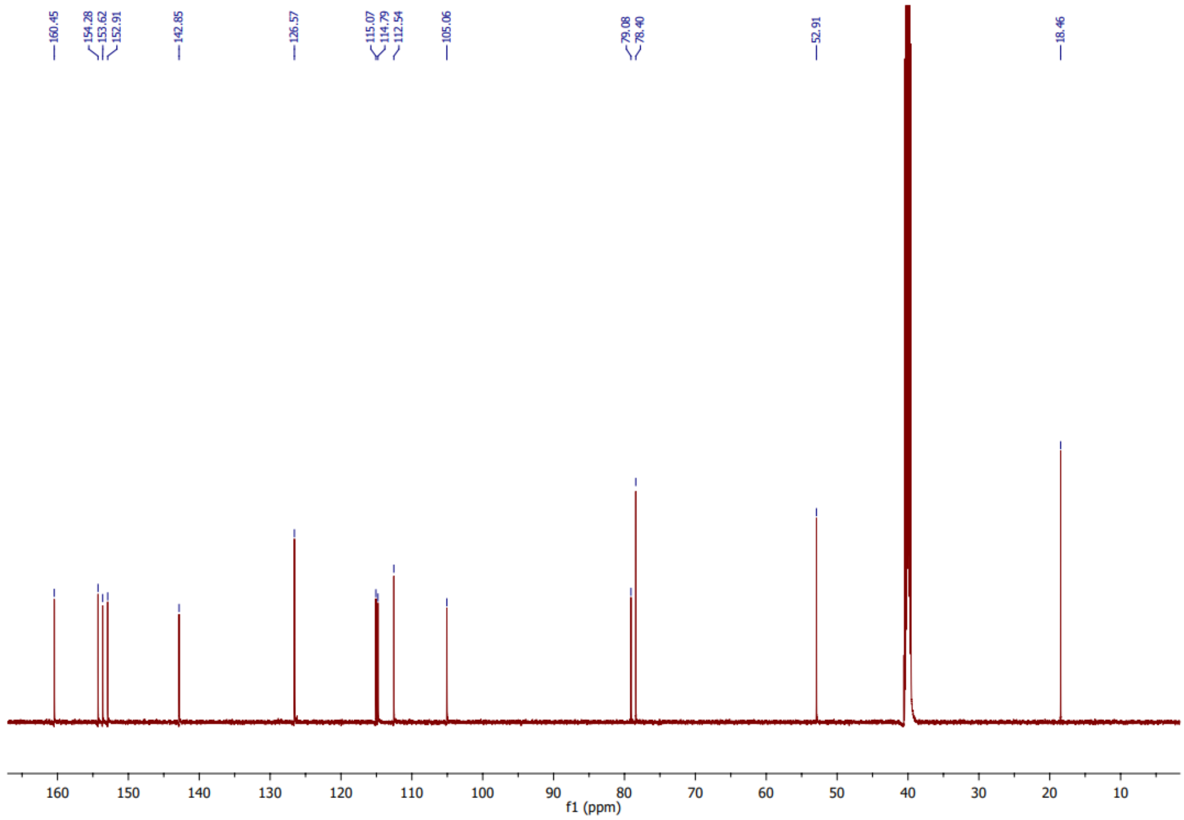

Supplementary Figure 51. 1H NMR (top) and 13C NMR (bottom) spectra of compound **14b** ($CDCl_3$)

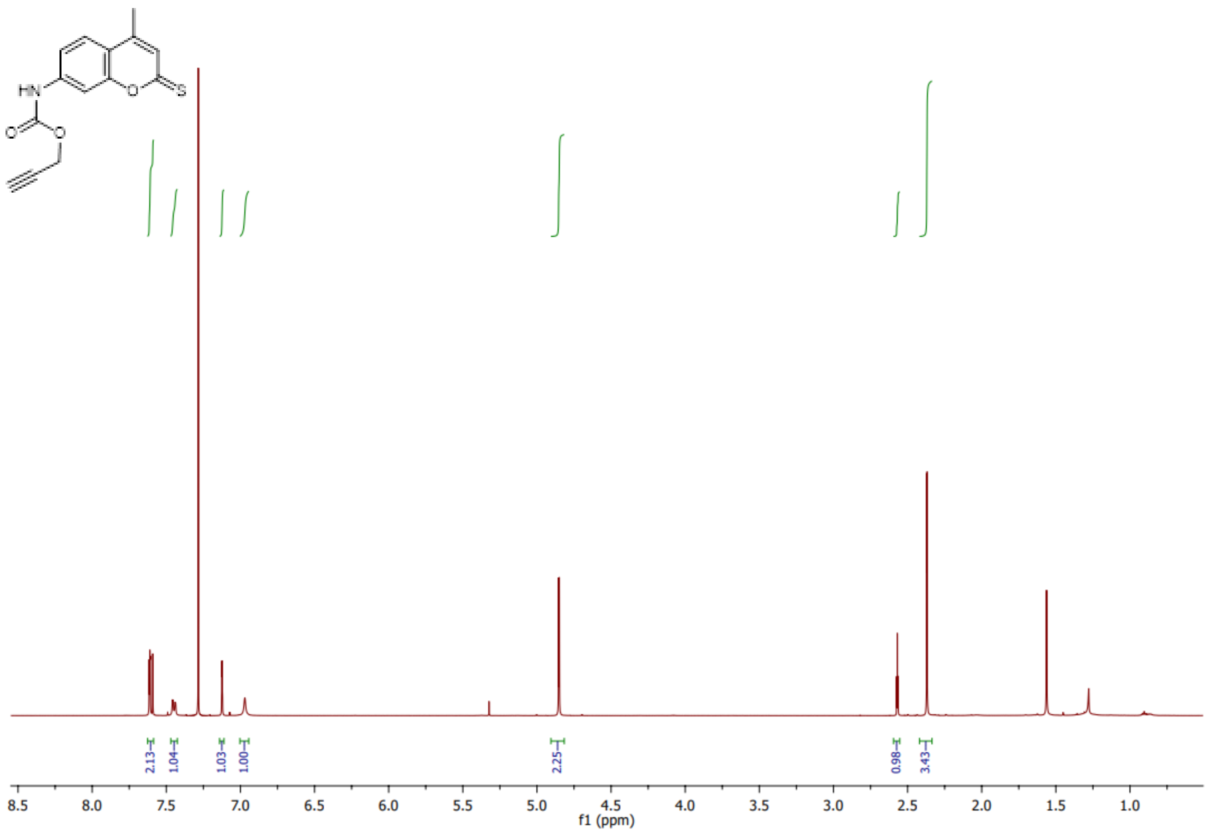

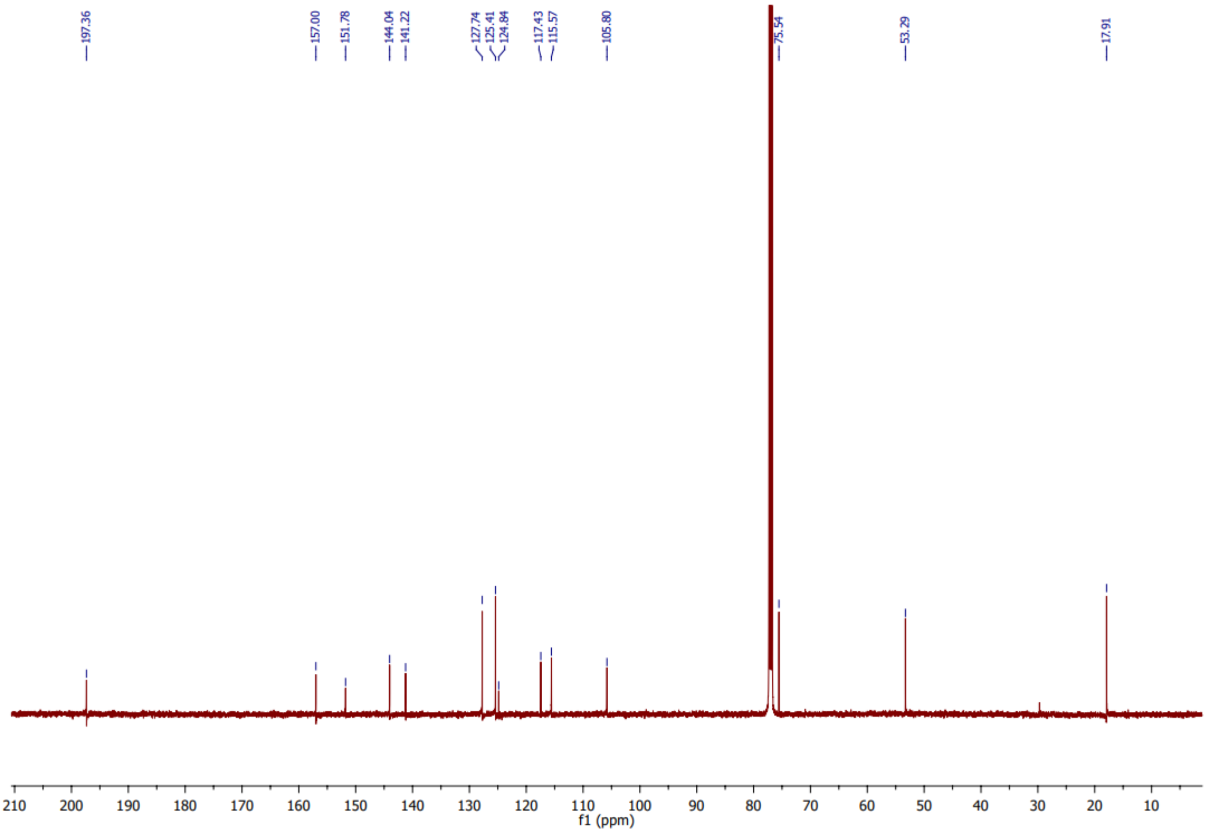

Supplementary Figure 52. 1H NMR (top) and 13C NMR (bottom) spectra of precursor compound **15** ($CDCl_3$)

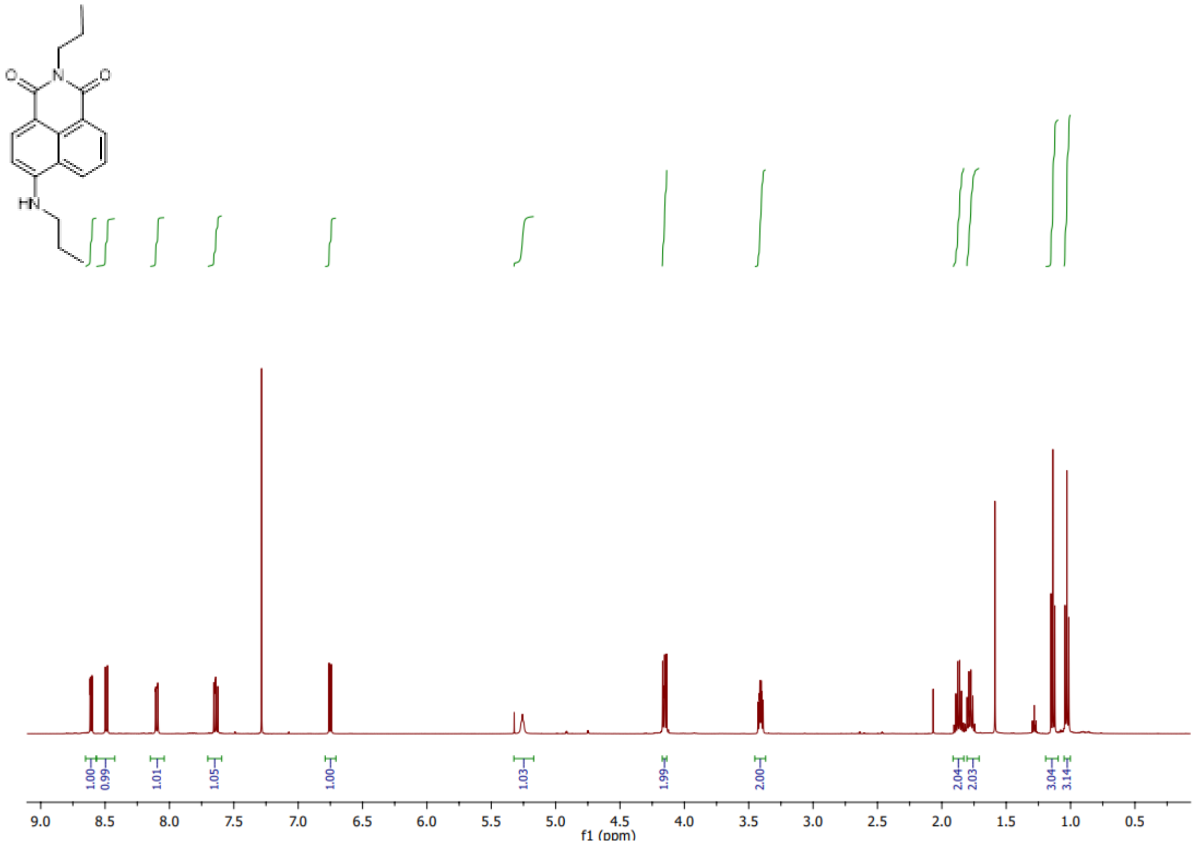

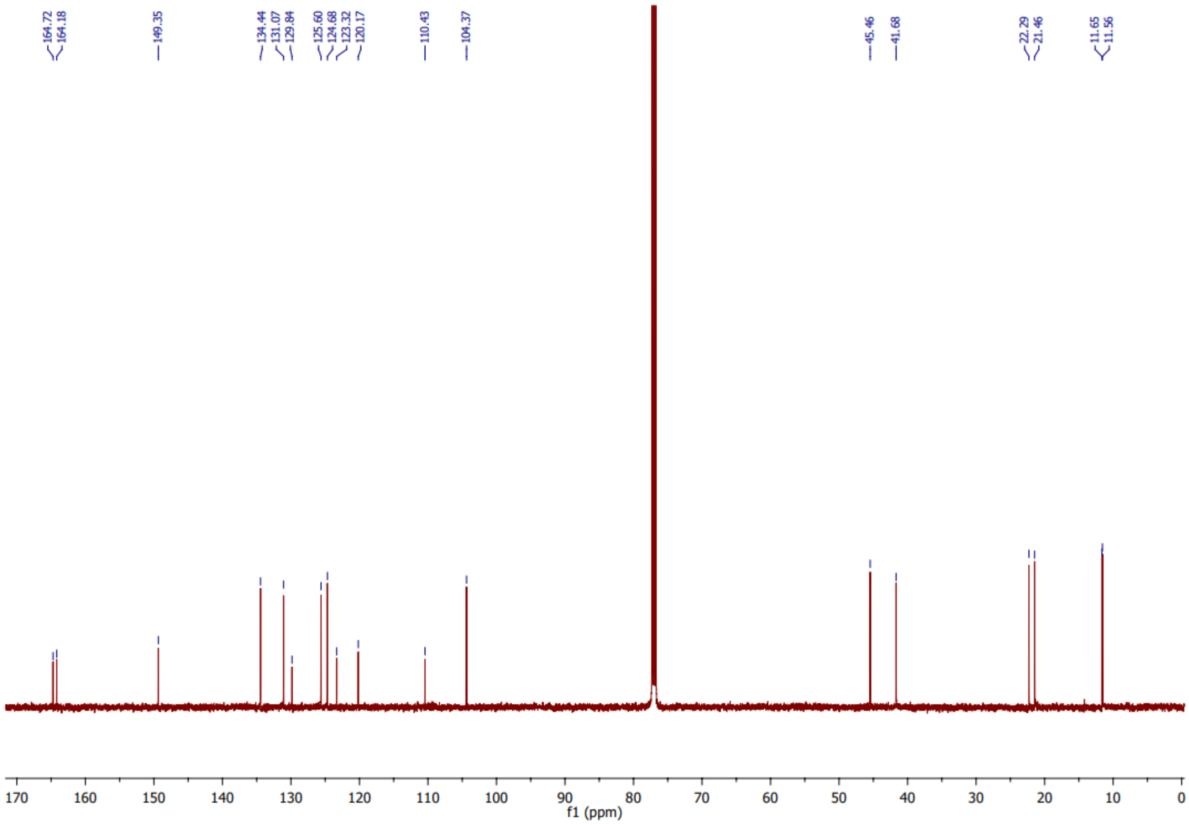

Supplementary Figure 53. 1H NMR (top) and 13C NMR (bottom) spectra of compound **15** ($CDCl_3$)

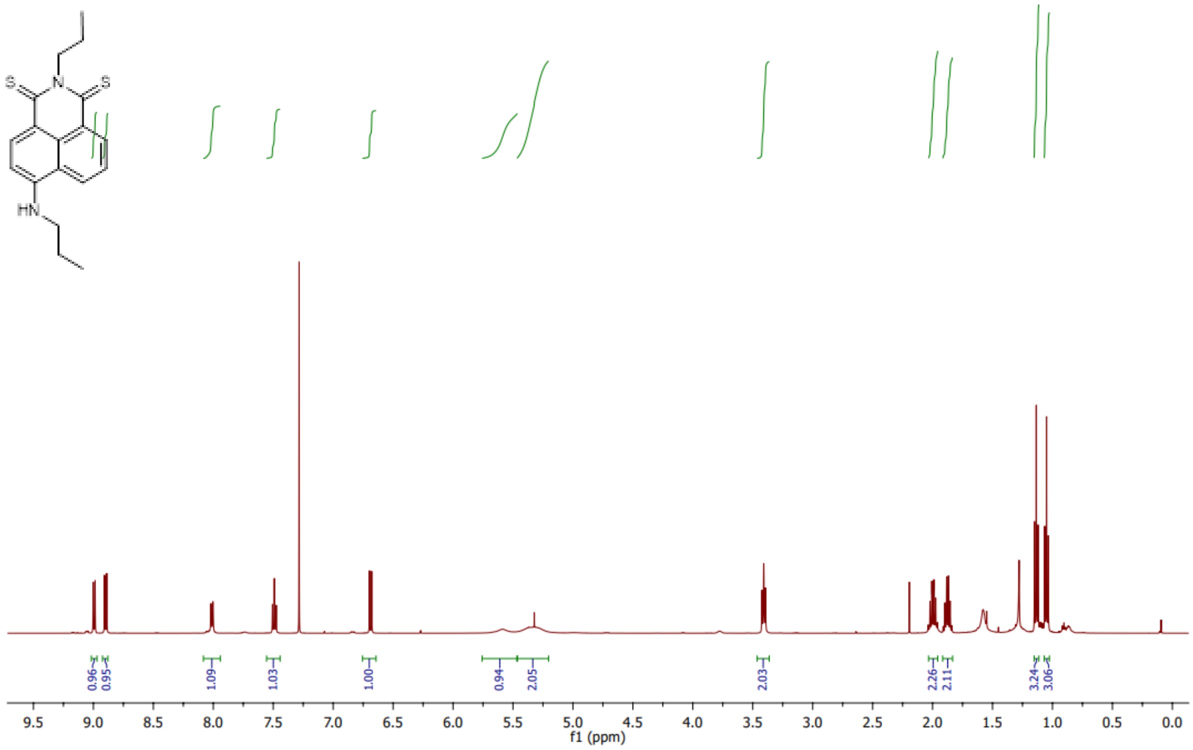

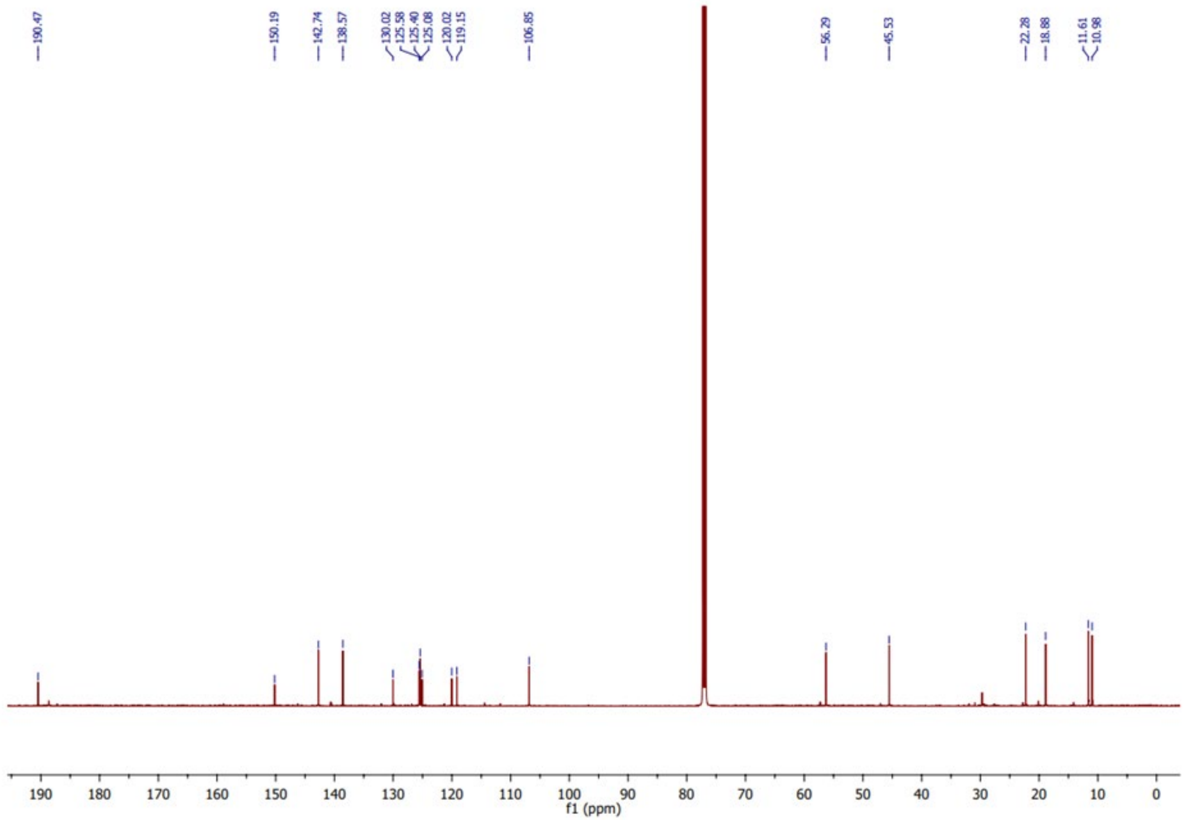

Supplementary Figure 54. 1H NMR (top) and 13C NMR (bottom) spectra of compound **15a** ($CDCl_3$)

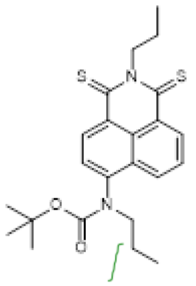

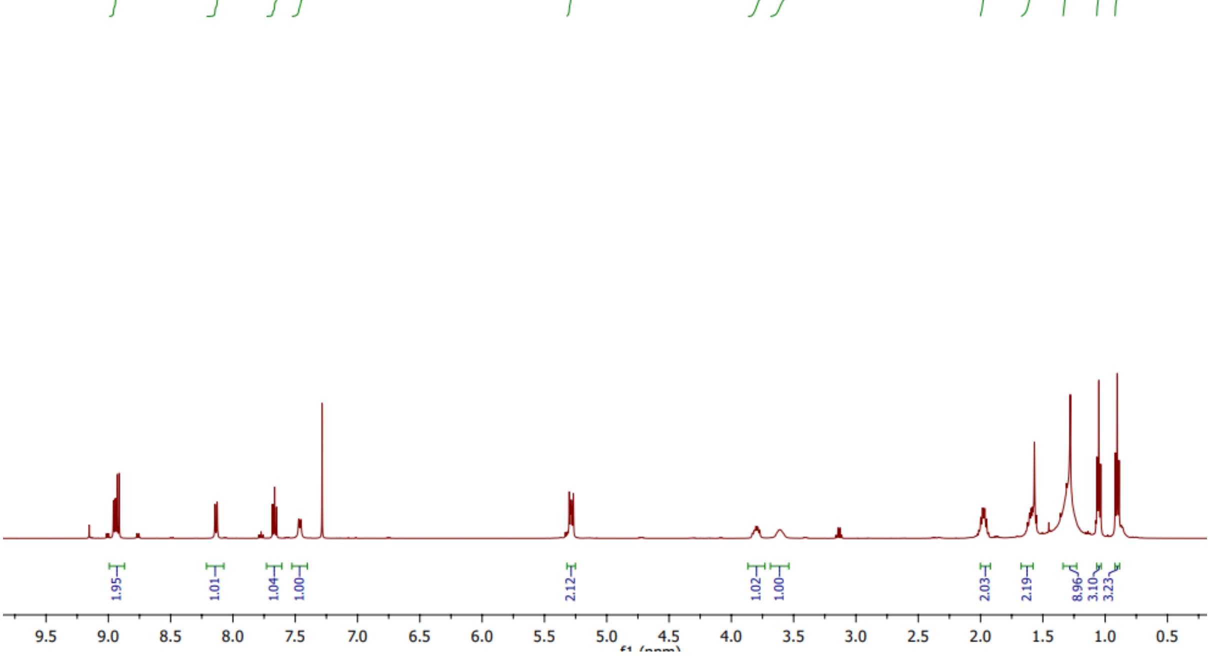

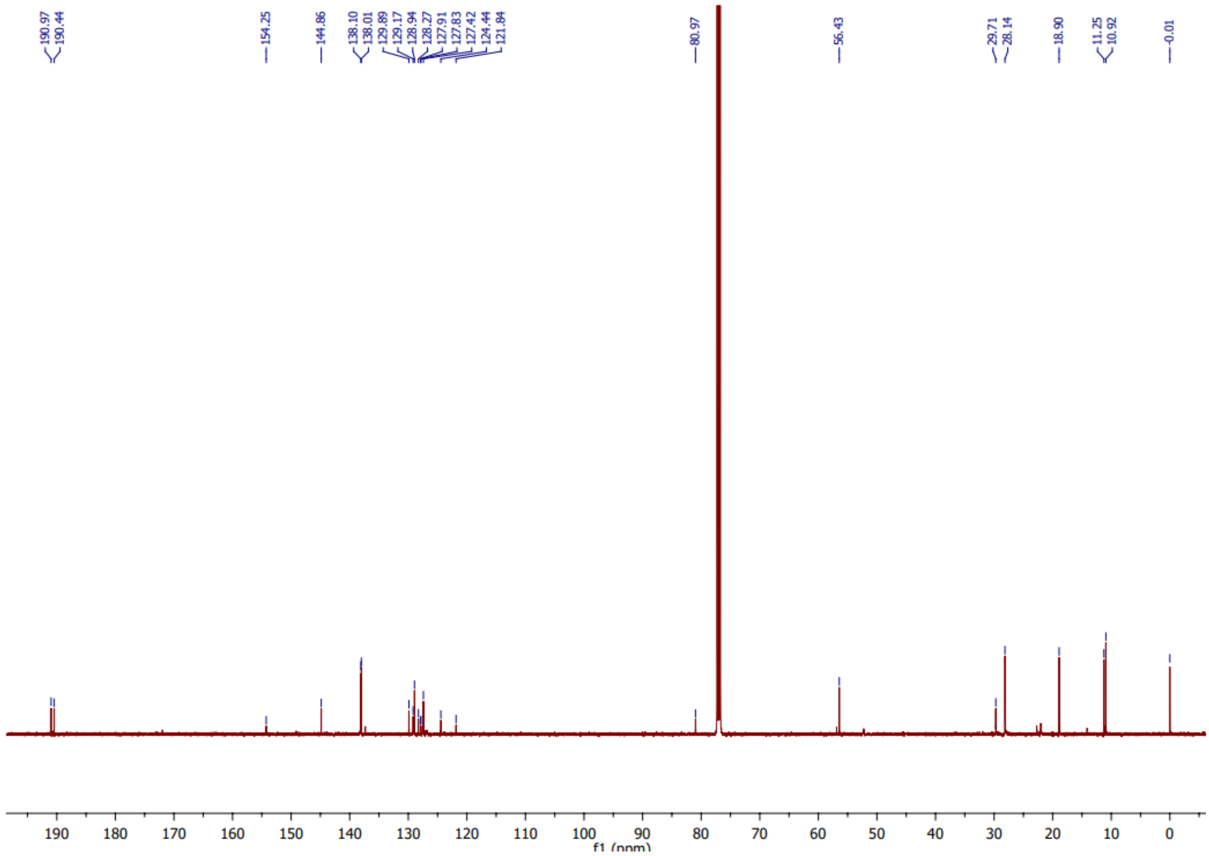

Supplementary Figure 55. 1H NMR (top) and 13C NMR (bottom) spectra of compound **15b** ($CDCl_3$)

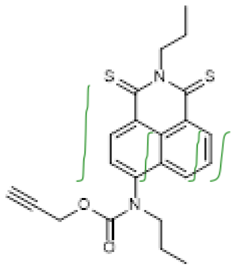
S
N
S
N
O
O

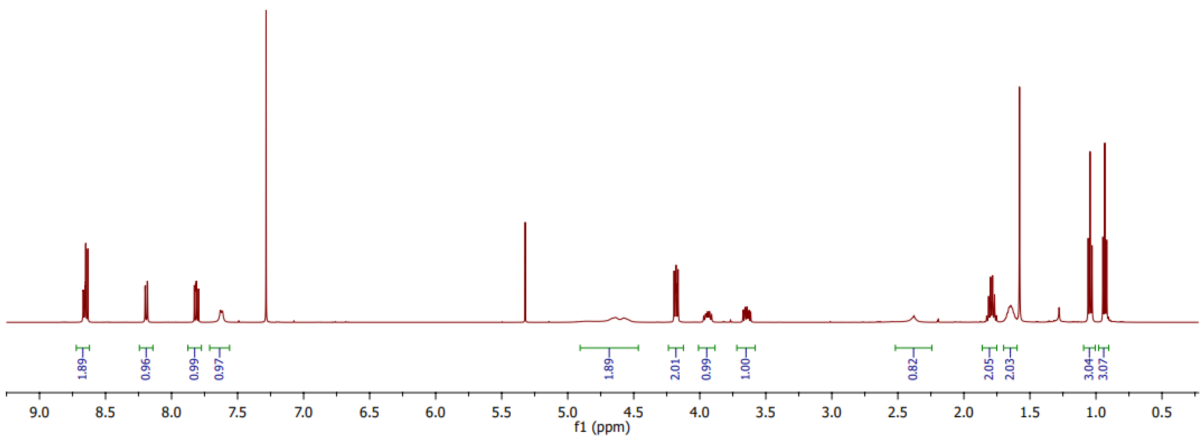
1.89
0.96
0.99
0.97
1.89
2.01
0.99
1.00
0.82
2.05
2.03
3.04
3.07
9.0 8.5 8.0 7.5 7.0 6.5 6.0 5.5 5.0 4.5 4.0 3.5 3.0 2.5 2.0 1.5 1.0 0.5
f1 (ppm)

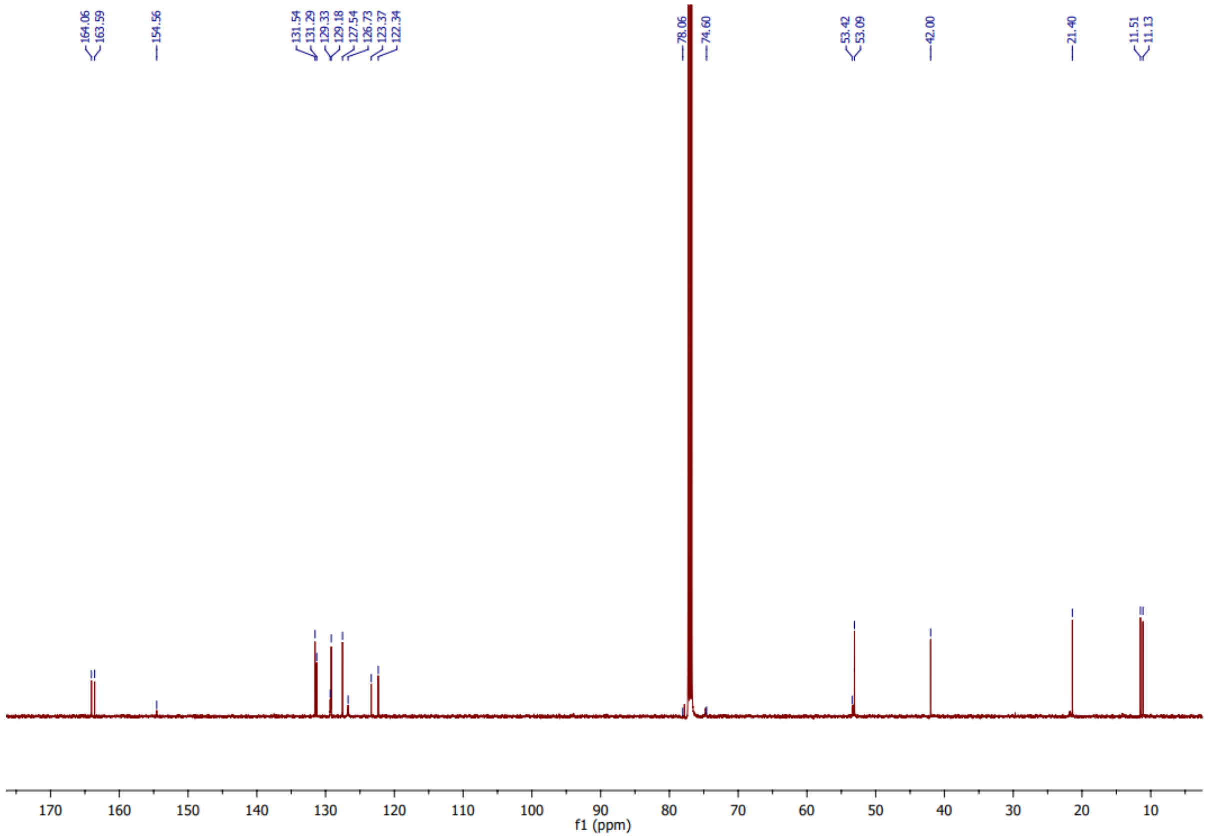
164.06
163.59
154.56
131.54
131.29
129.33
129.18
127.54
126.73
123.37
122.34
78.06
74.60
53.42
53.09
42.00
21.40
11.51
11.13
170 160 150 140 130 120 110 100 90 80 70 60 50 40 30 20 10
f1 (ppm)

Supplementary Figure 56. 1H NMR (top) and 13C NMR (bottom) spectra of compound **16a** (DMSO-d_6)

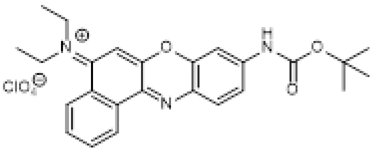

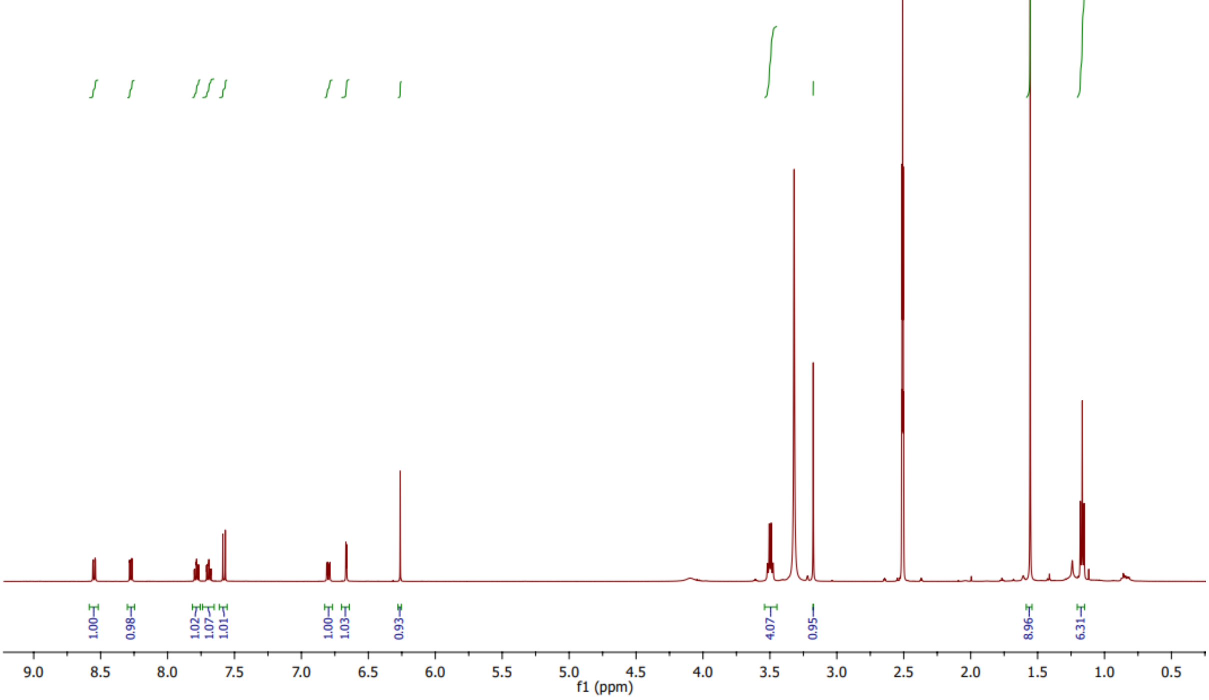

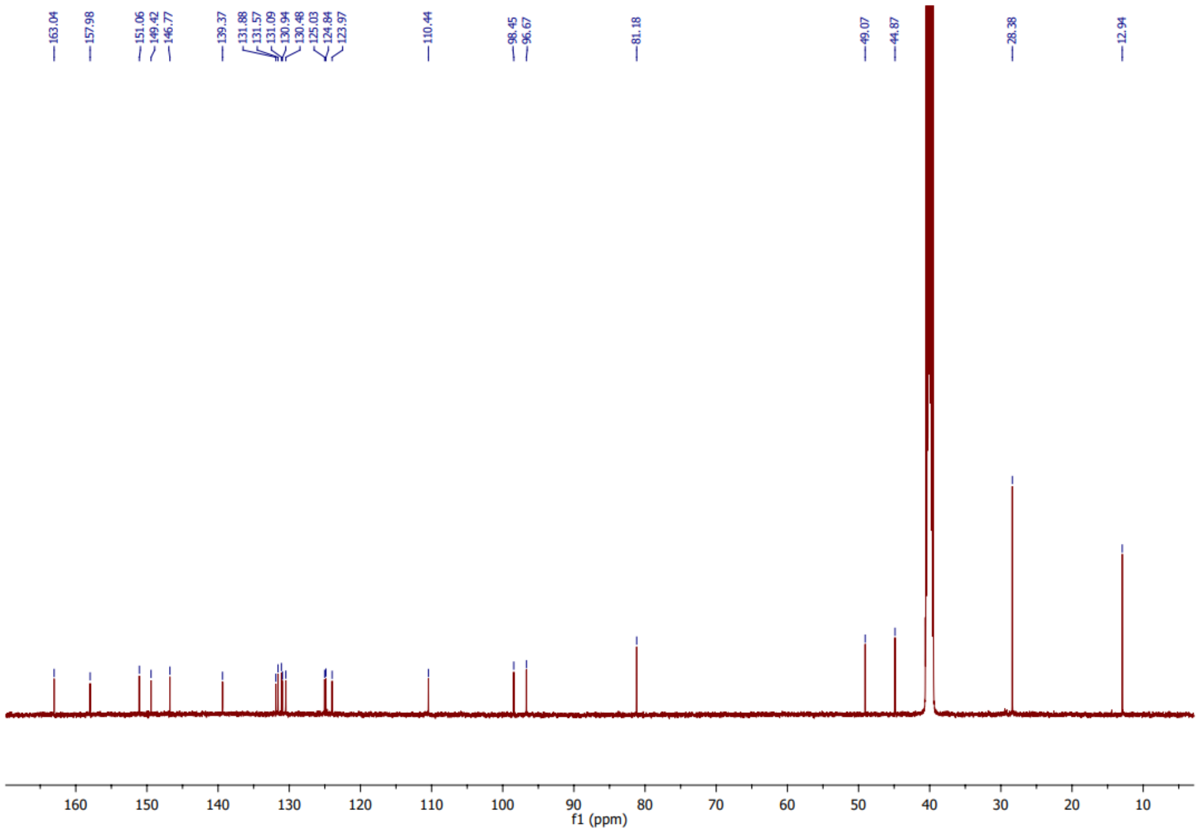

Supplementary Figure 57. 1H NMR (top) and 13C NMR (bottom) spectra of compound **16b** (DMSO-d6)

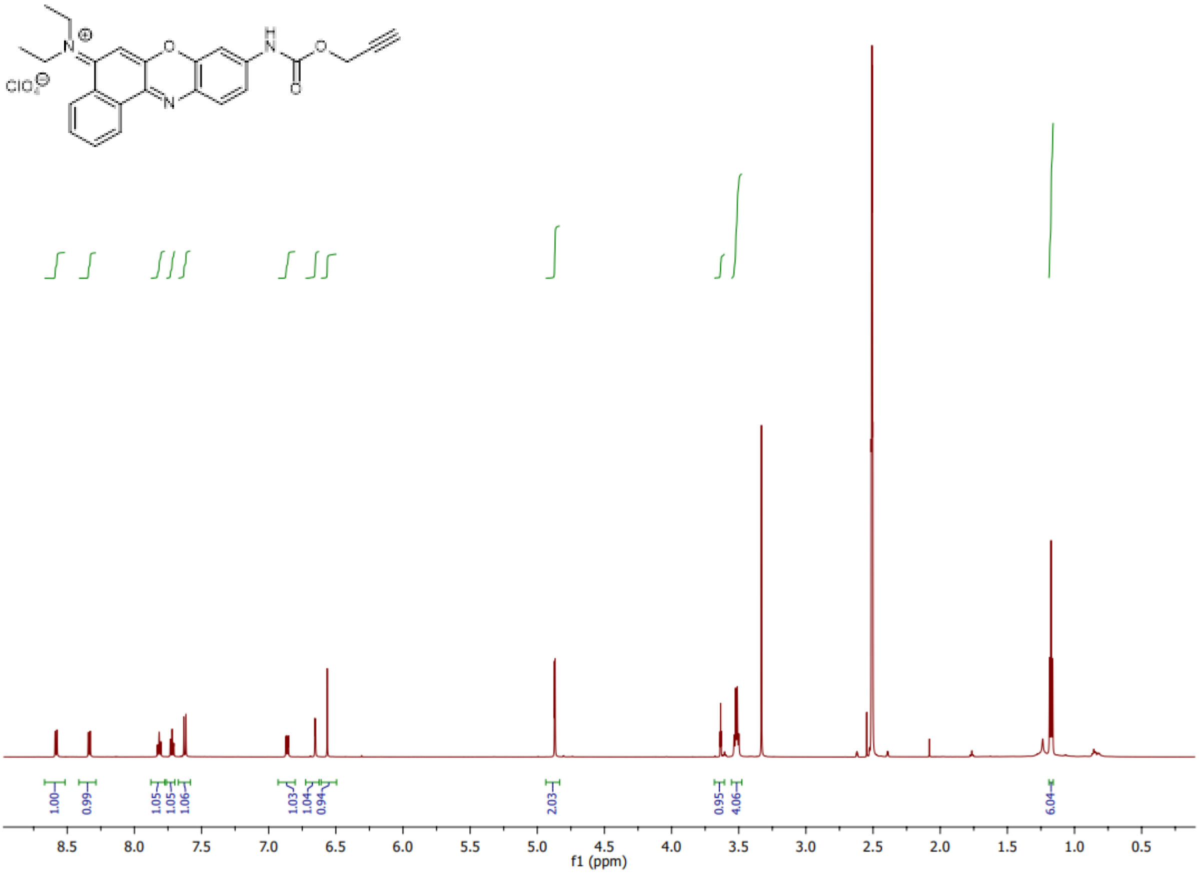

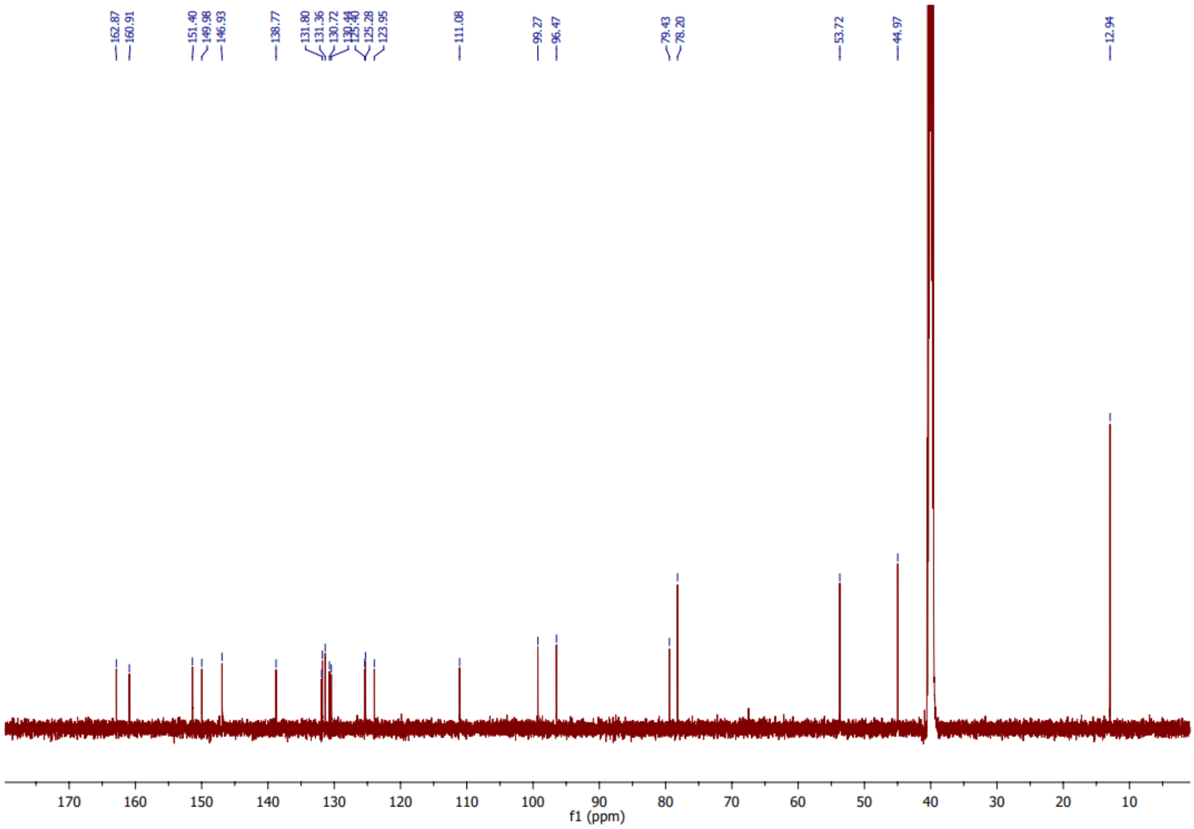

Supplementary Figure 58. 1H NMR spectrum of compound **17a** (MeOD)

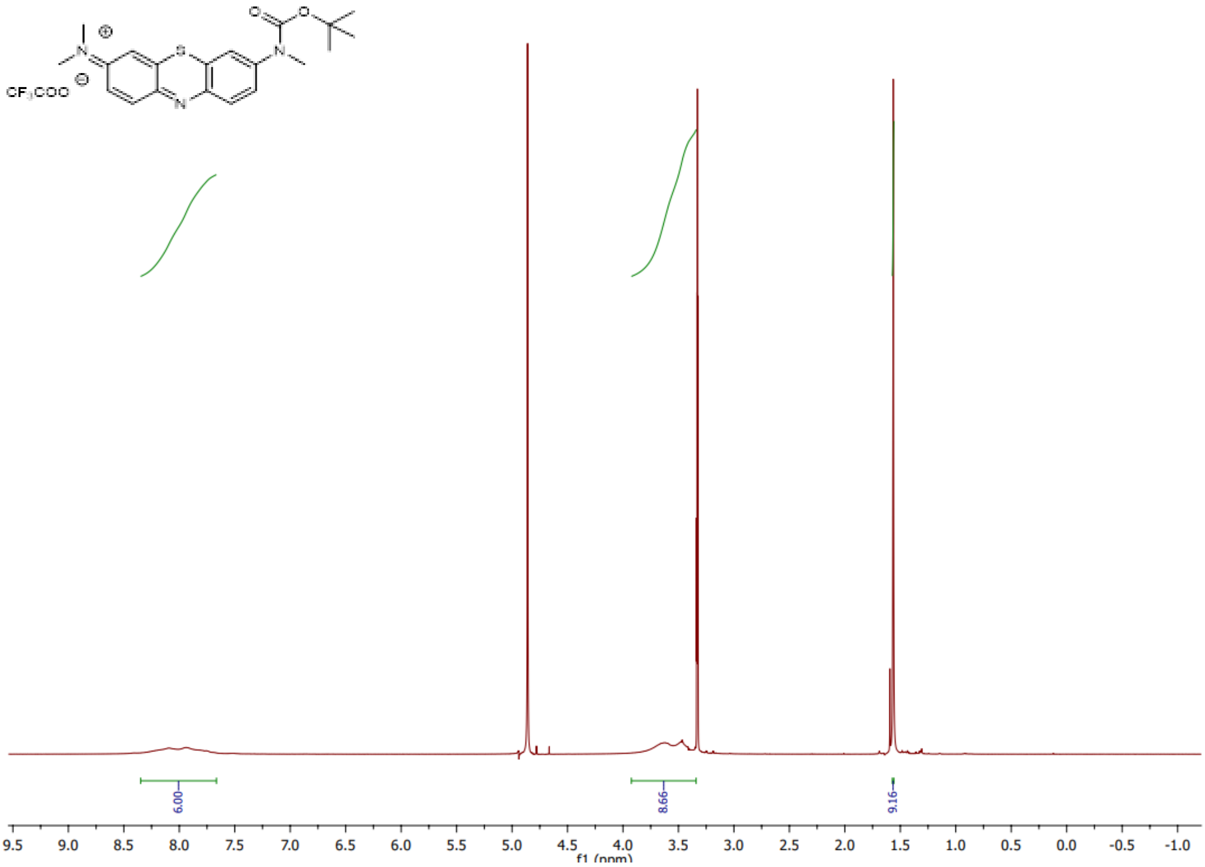

Supplementary Figure 59. 1H NMR spectrum of compound **17b** (MeOD)

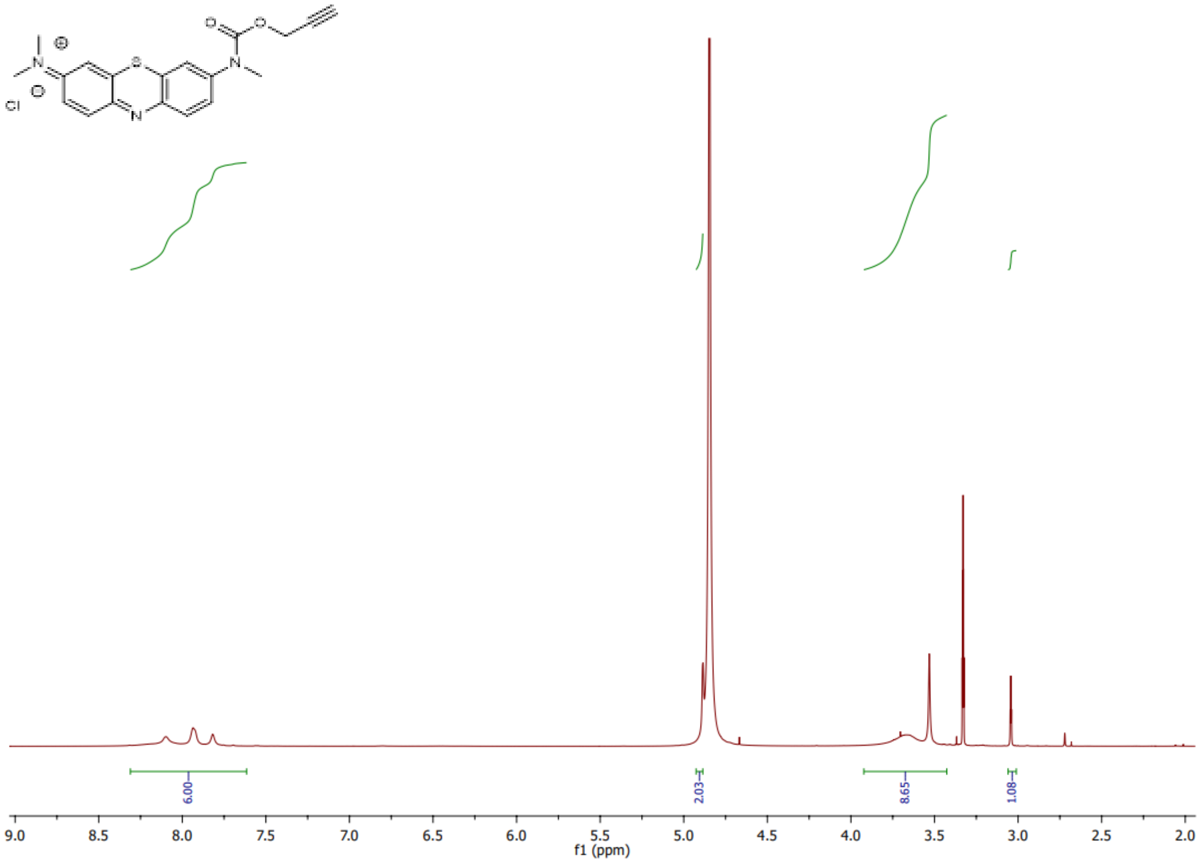

Supplementary Figure 60. HPLC and HRMS traces of compound **17a**

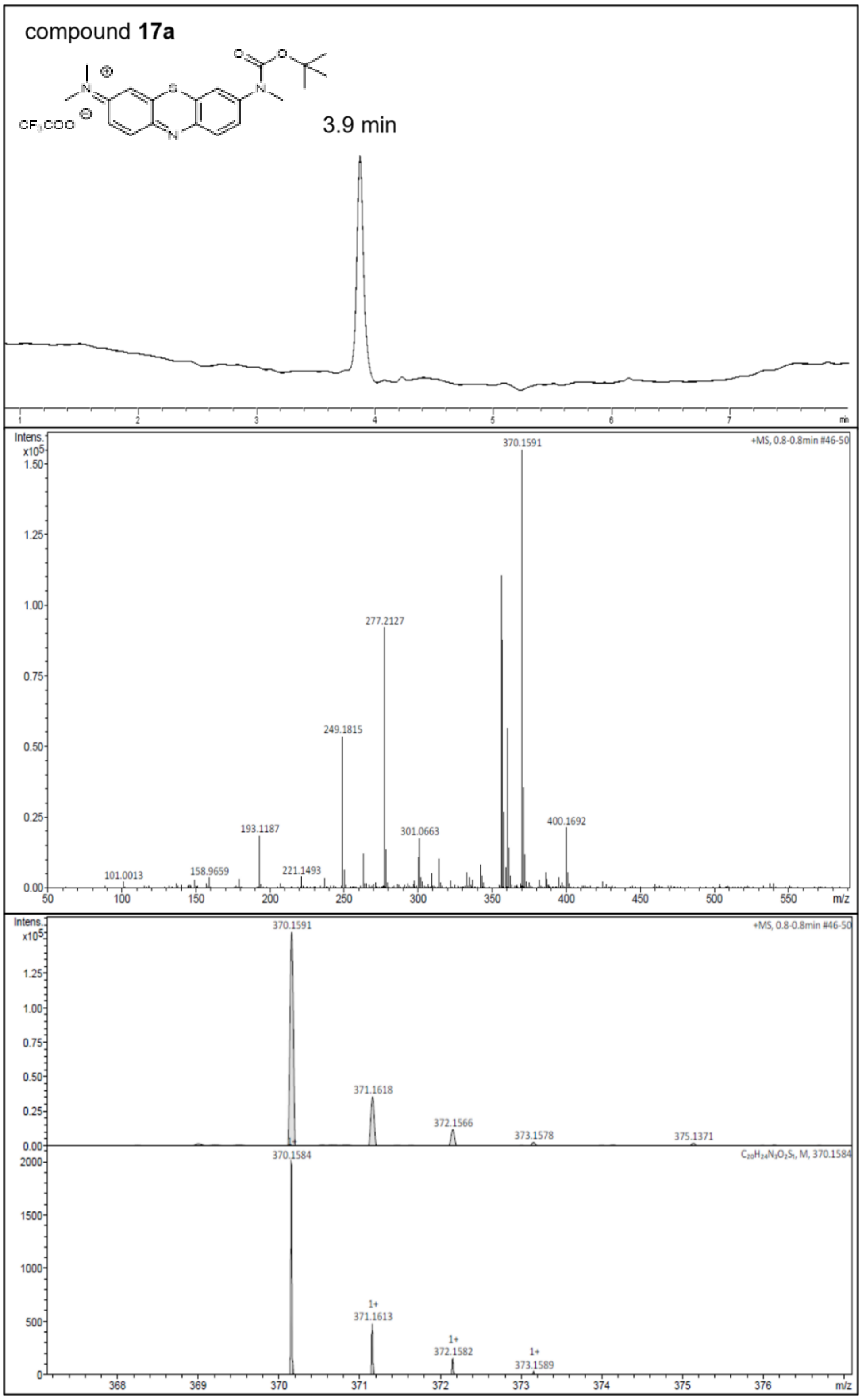

Supplementary Figure 61. HPLC and HRMS traces of compound **17b**

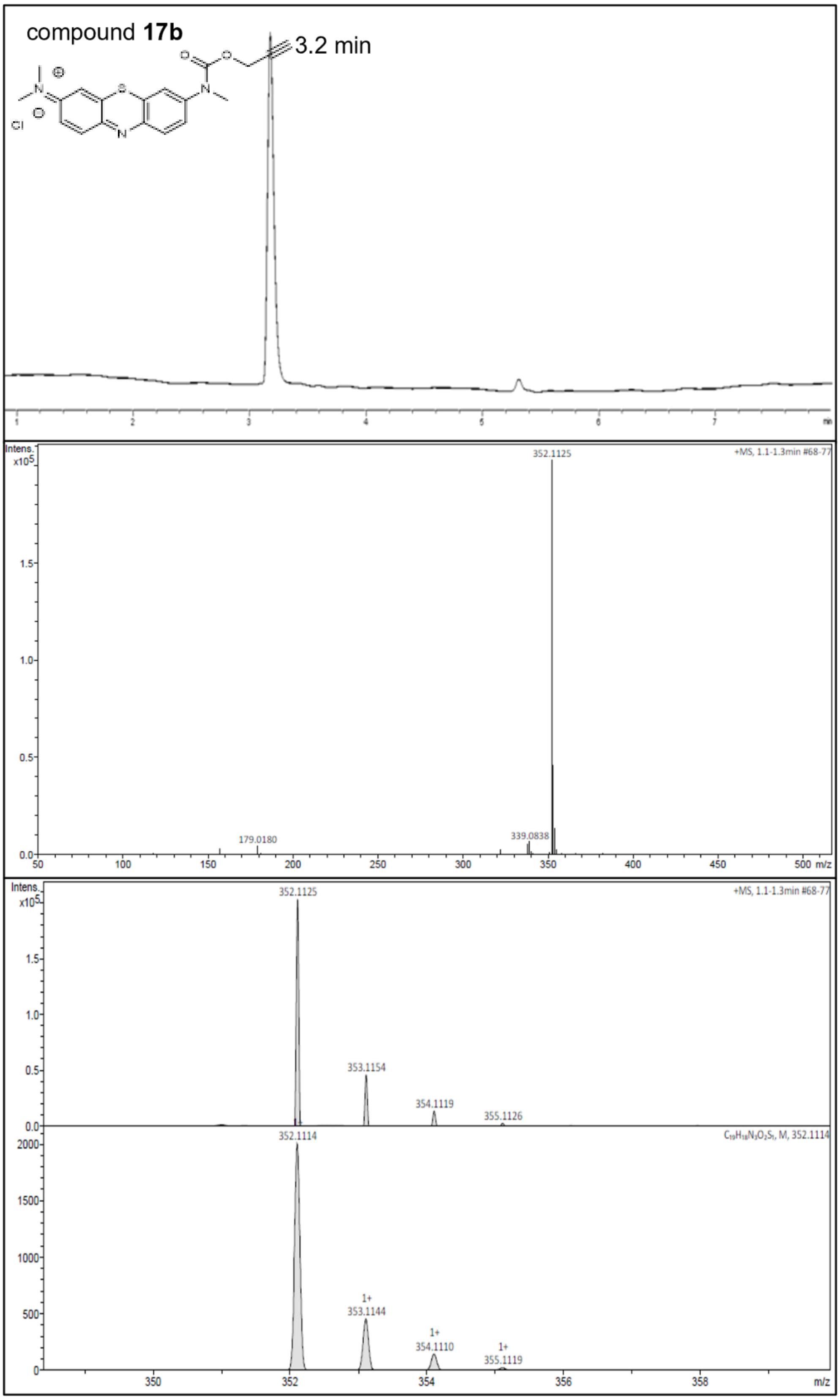

Computational Modelling

compound **1**

C	-1.11956	-2.35563	0.00007
C	0.25082	-2.48572	0.00005
C	1.10969	-1.32461	0.00002
C	0.52164	-0.02354	0.
C	-0.90884	0.13133	0.00005
C	-1.71778	-1.08514	0.00007
N	1.30619	1.10716	-0.0001
Se	0.07697	2.54764	-0.00018
N	-1.42749	1.36896	-0.00002
N	2.4367	-1.56091	0.00003
C	3.55798	-0.59648	0.00004
C	4.88507	-1.35088	0.00021
N	-3.1677	-1.06584	0.00008
O	-3.76934	-2.20189	-0.00101
O	-3.77939	0.03833	0.0013
H	-1.76612	-3.21685	0.00007
H	0.70335	-3.46841	0.00008
H	2.71369	-2.53403	0.00002
H	3.45962	0.05242	-0.86818
H	3.45947	0.05257	0.86813
H	5.71131	-0.64073	0.00021
H	4.99184	-1.97888	0.88783
H	4.99198	-1.97904	-0.88728

compound **2**

C	-2.06566	-2.40645	0.28822
C	-0.71584	-2.21001	0.5819
C	-0.19146	-0.92199	0.56924
C	-0.9703	0.20621	0.24284
C	-2.39768	0.01062	-0.08215
C	-2.90507	-1.3336	-0.03452
N	-0.46955	1.45697	0.20858
Se	-1.89773	2.57724	-0.29277
N	-3.08605	1.1141	-0.38951
N	1.20952	-0.68214	0.88582
C	1.61464	-0.40852	2.25752
C	1.79819	1.11517	2.54705
C	2.17641	-0.70369	-0.17165
O	3.38377	-0.31832	0.267
C	4.60862	-0.22031	-0.6819
C	5.68293	0.29608	0.26938
O	1.85921	-1.07924	-1.3029
C	4.915	-1.62387	-1.20224
C	4.27203	0.78219	-1.78431
N	-4.27751	-1.63321	-0.31481
O	-4.65467	-2.86387	-0.2441
O	-5.07636	-0.68692	-0.61917
H	-2.49903	-3.39185	0.30002
H	-0.0875	-3.0585	0.81194
H	0.84009	-0.8158	2.89778
H	2.57083	-0.90216	2.43154
H	2.00058	1.21599	3.61306
H	2.63799	1.50617	1.98076
H	0.89746	1.65306	2.27401
H	6.62351	0.40678	-0.27159
H	5.40446	1.26644	0.67803

H	5.83878	-0.40042	1.09234
H	5.83712	-1.5916	-1.78504
H	4.12081	-1.99639	-1.84431
H	5.0645	-2.31729	-0.3745
H	3.96386	1.73582	-1.35654
H	5.16379	0.95439	-2.38937
H	3.48424	0.41501	-2.43746

4. Supplementary References

1. Bray, T. L. *et al*. Bright insights into palladium-triggered local chemotherapy. *Chem. Sci.* **9**, 7354–7361 (2018).

2. Ortega-Liebana, M. C. *et al.* Truly-biocompatible gold catalysis enables vivo-orthogonal intra-CNS release of anxiolytics. *Angew. Chem. Int. Ed.* **61**, e202111461 (2022).

3. Della Pina, C., Falletta, E., Rossi, M. & Sacco, A. Selective deactivation of gold catalyst. *J. Catal.* **263**, 92-97 (2009).
